# Supplementary material for: Φ-Space ST: A platform-agnostic method to identify cell states in spatial transcriptomics studies
Source: Cell Rep Methods. 2026 Jun 12;6(8):101483. doi: 10.1016/j.crmeth.2026.101483 (PMC13494538; doi:10.1016/j.crmeth.2026.101483)
Supplement: Supplementary file 1 [file mmc1.pdf]

**Cell Reports Methods, Volume *6***

## Supplemental information

# Φ-Space ST: A platform-agnostic method to identify cell states in spatial transcriptomics studies

**Jiadong Mao, Jarny Choi, and Kim-Anh Lê Cao**

# S1 Supplementary figures

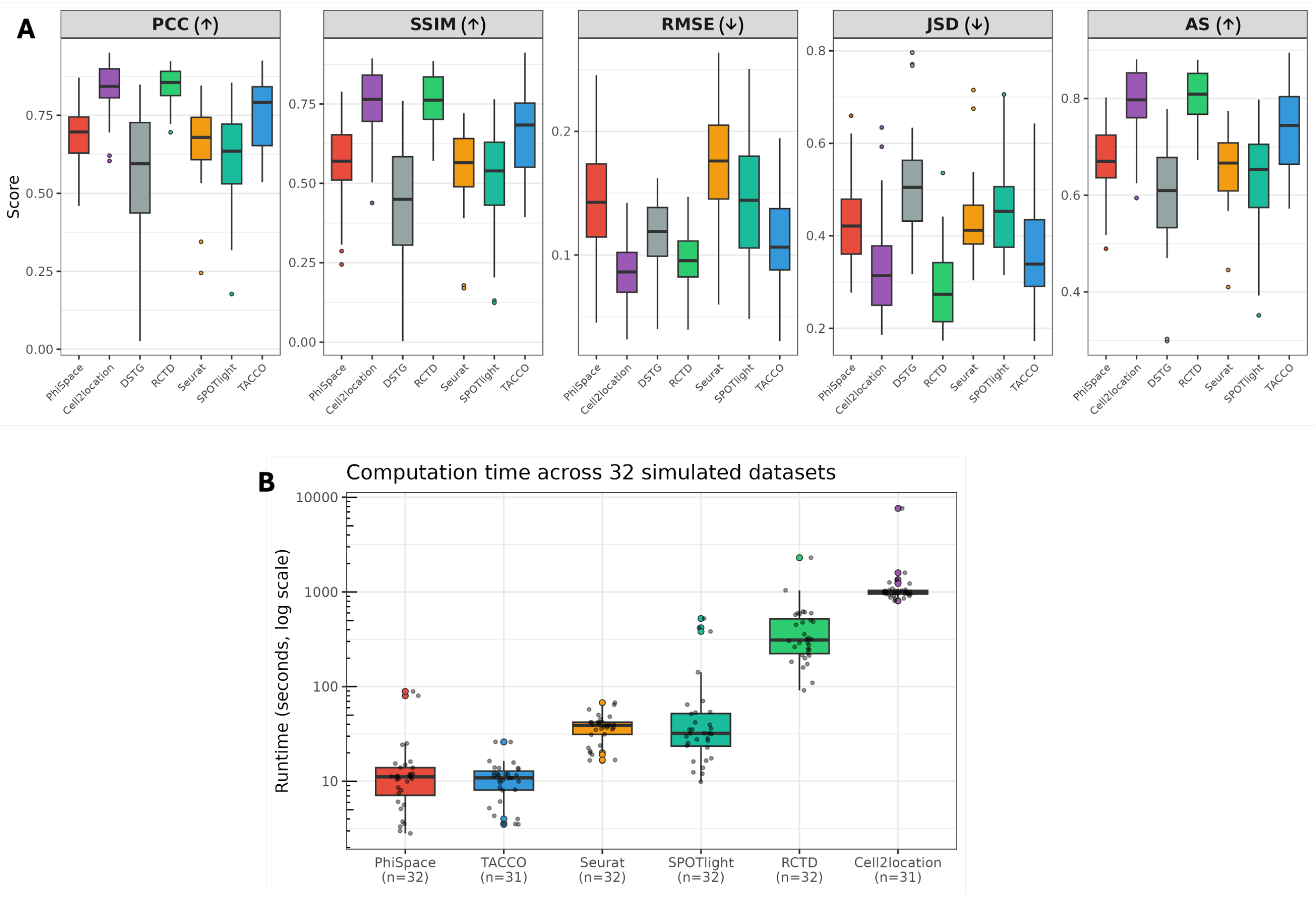


**Fig S1: Benchmarking Φ-Space ST against 6 other methods, related to Figure 2. A:** Following Li et al.[1], we used four metrics measuring the performance of 7 methods for cell type deconvolution. ↑: higher metric means better method; ↑: lower metric means better method. PCC: Pearson correlation coefficient; SSIM: structural similarity index; RMSE: root mean square error; JSD: Jensen-Shannon divergence. **B:** Runtime of six methods over simulated datasets.

# S2 Supplementary tables

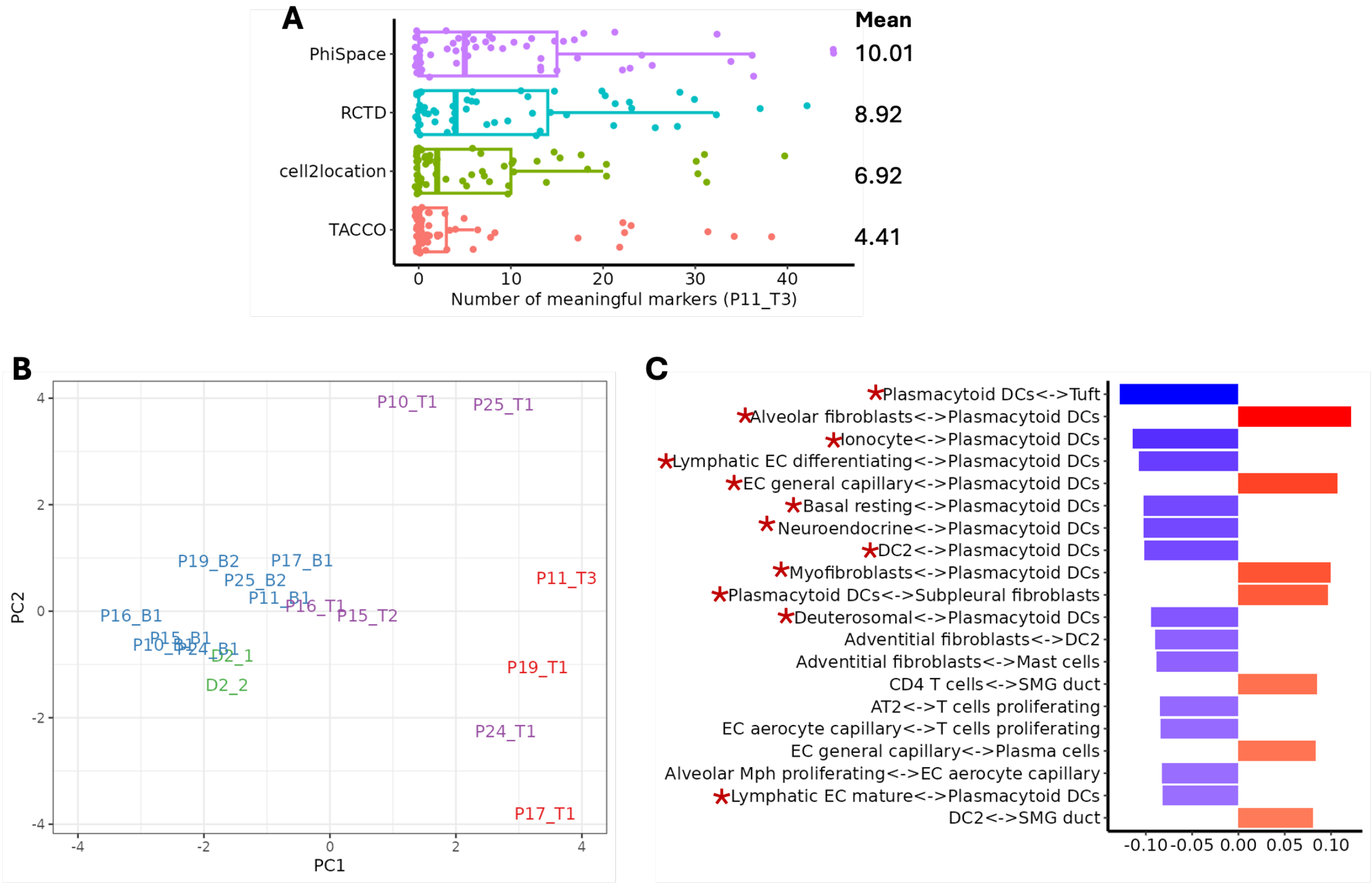


**Fig S2: Immune cell type interactions in 10x Visium lung cancer samples, related to Figure 2.** **A:** Boxplot comparing number of meaningful markers for the prediction of all cell types in P11_T3. **B:** Similar to Fig 2A, but now focusing on immune cell types, we filtered out correlations between non-immune cell types from the correlation matrices and recomputed their first two PCs. The separation between tumorous and non-tumorous tissues was largely retained compared to Fig 2A. **C:** Top 20 cell type interactions involving immune cell types contributing to PC1: 12/20 interactions (asterisks) involved plasmacytoid dendritic cells (pDCs).

# References


[1] Bin Li, Wen Zhang, Chuang Guo, Hao Xu, Longfei Li, Minghao Fang, Yinlei Hu, Xinye Zhang, Xinfeng Yao, Meifang Tang, Ke Liu, Xuetong Zhao, Jun Lin, Linzhao Cheng, Falai Chen, Tian Xue, and Kun Qu. Benchmarking spatial and single-cell transcriptomics integration methods for transcript distribution prediction and cell type deconvolution. *Nat. Methods*, 19(6):662–670, 2022. doi: 10.1038/s41592-022-01480-9.

[2] José E Chacón and Tarn Duong. *Multivariate kernel smoothing and its applications*. CRC Press, London, England, 1st edition edition, 2020.

[3] Tarn Duong. *ks: Kernel Smoothing*, 2024. URL `https://CRAN.R-project.org/package=ks`. R package version 1.14.2.

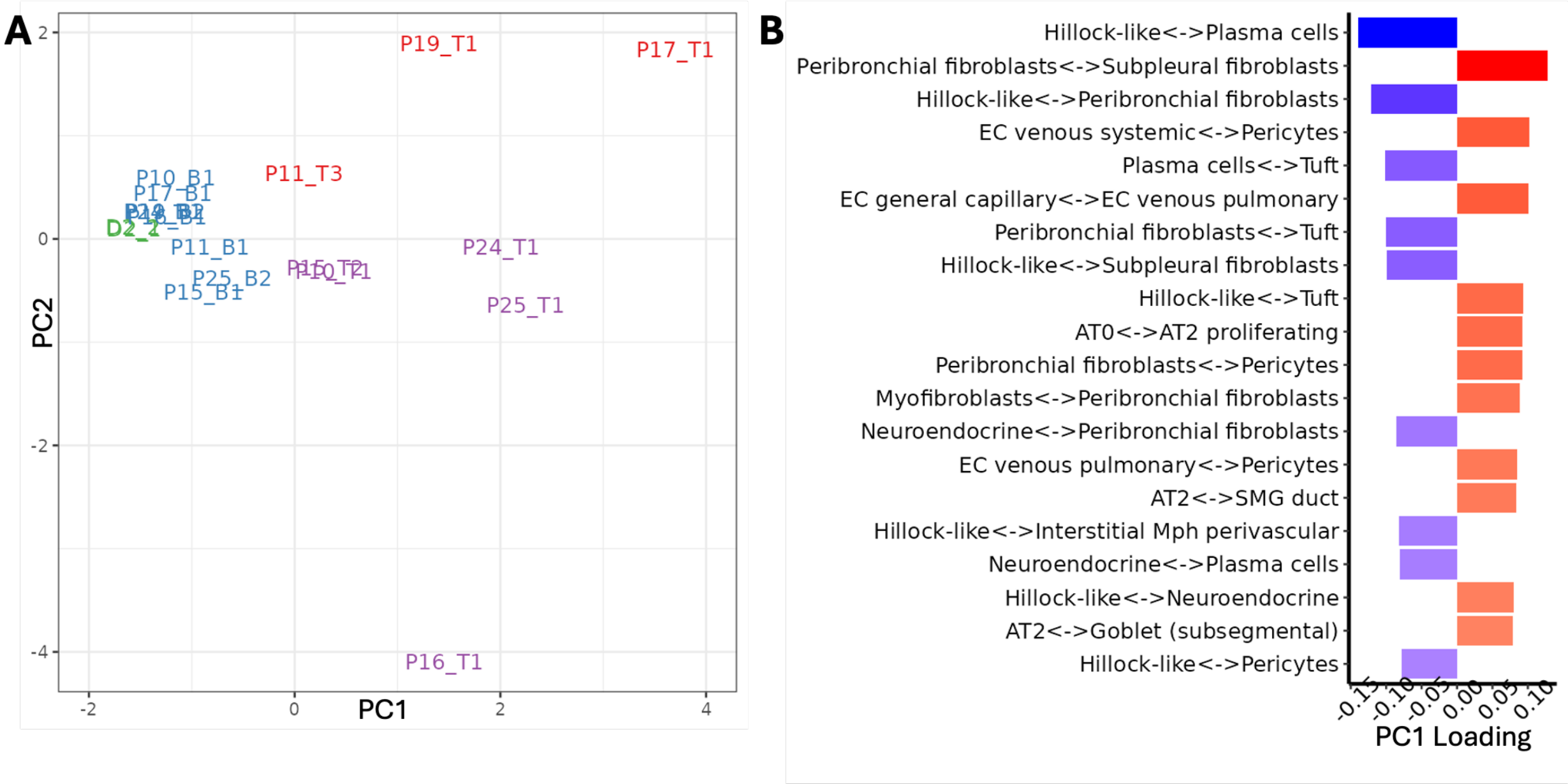


**Fig S3: Cell type co-presence analysis using RCTD deconvolution results, related to Figure 2.** **A:** The first two principal components (PCs) of cell type co-presence matrices derived using RCTD deconvolution results. We observed separation of different disease conditions. However, two PCs were needed for separating LUAD, LUSC and non-cancerous samples instead of just PC1 as in the Φ-Space ST results in Fig 2D. Moreover, neither of the RCTD PCs here seems to correspond to prognosis (Fig 2E). **B:** Top 20 loading values (absolute value) contributing to PC1. Fibroblasts still plays a prominant role, similar to the Φ-Space ST results as in Fig 2F.

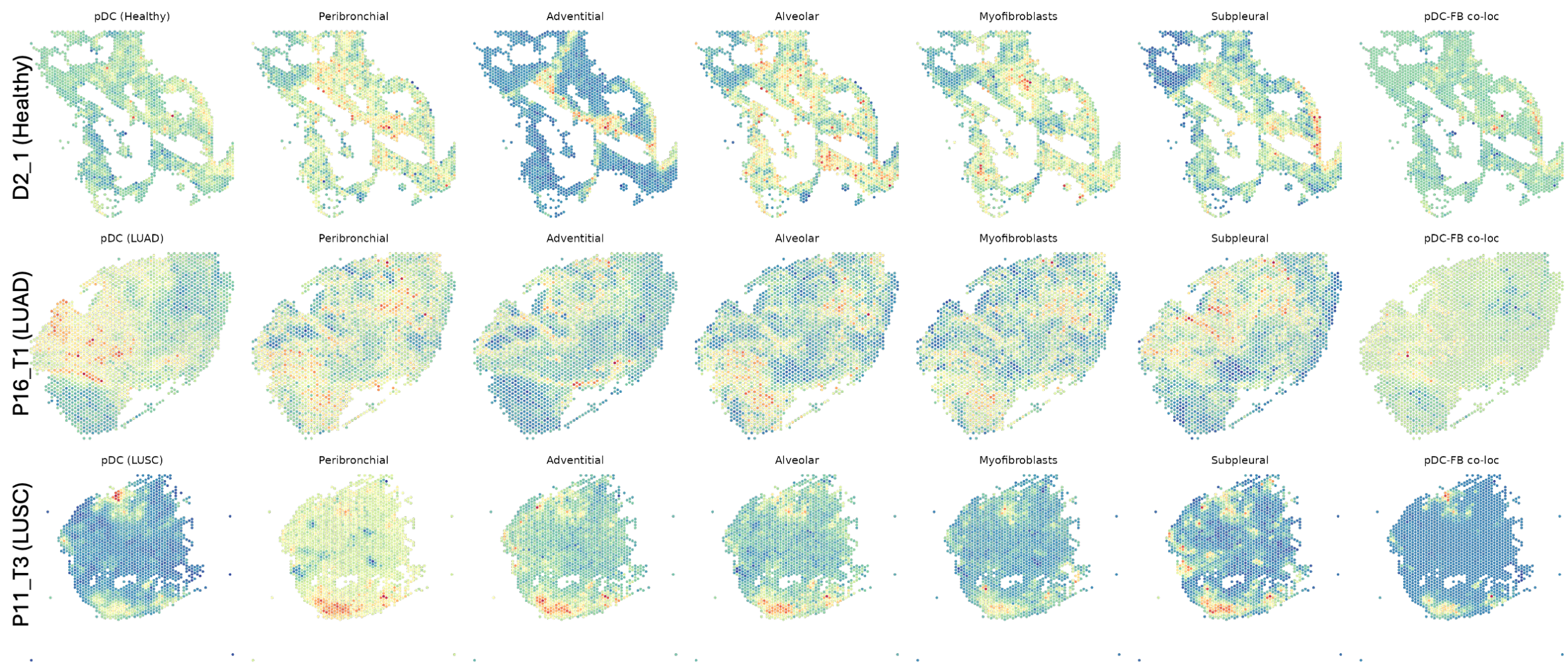


**Fig S4: Spatial heatmaps for pDC and fibroblasts in 3 representative Visium samples, related to Figure 2.** Spatial heatmaps of pDC score, fibroblast subtype scores and pDC–FB co-presence score (Spearman correlation) for representative healthy, LUAD and LUSC samples.

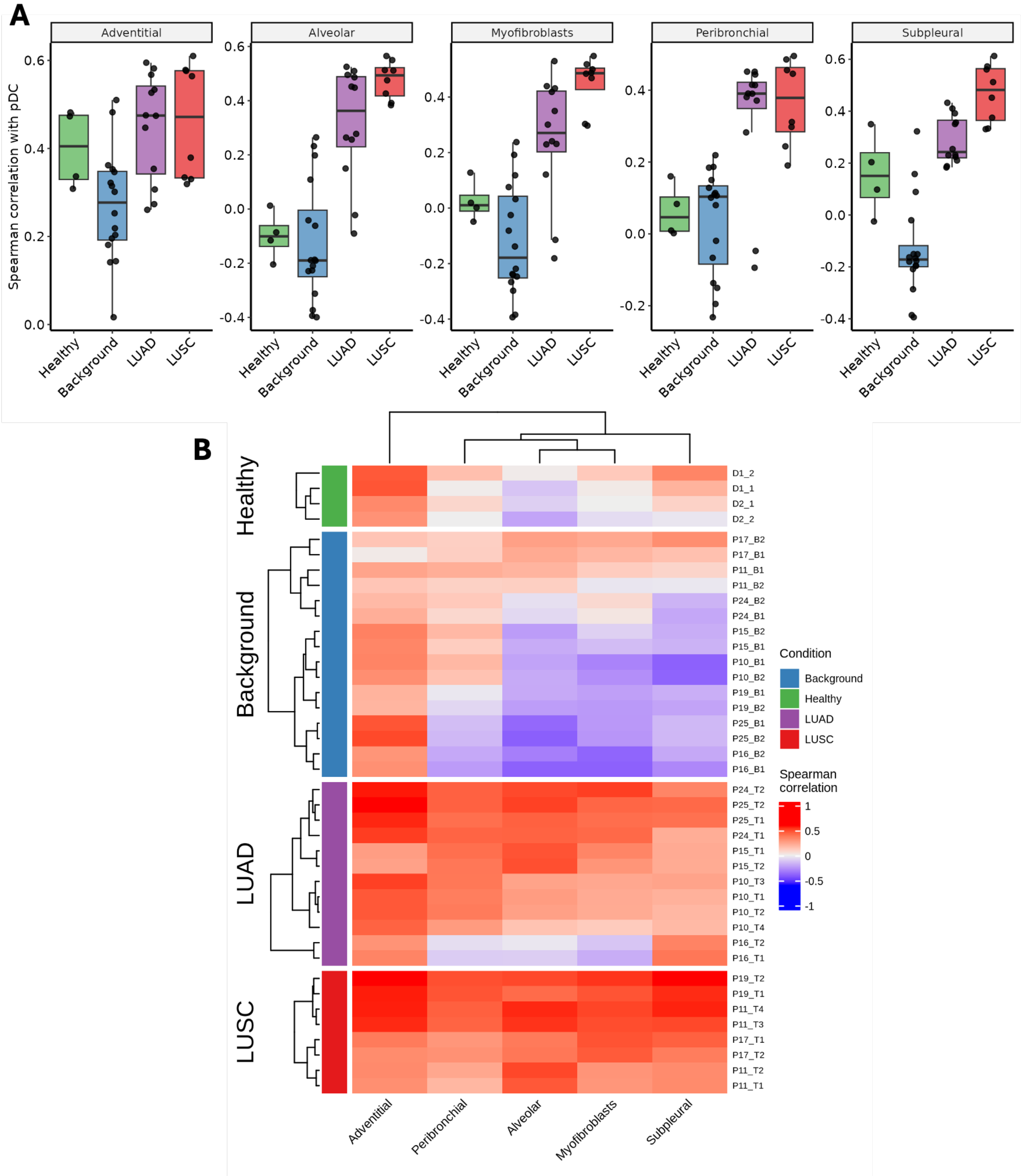


**Fig S5: Co-localisation of pDC and fibroblasts in all 40 Visium samples, related to Figure 2. A:** Boxplots for pDC–fibroblast Spearman correlation by condition (Healthy, Background, LUAD, LUSC) for each fibroblast subtype. **B:** Heatmap for sample by fibroblast subtype Spearman correlations with pDC, grouped by condition.

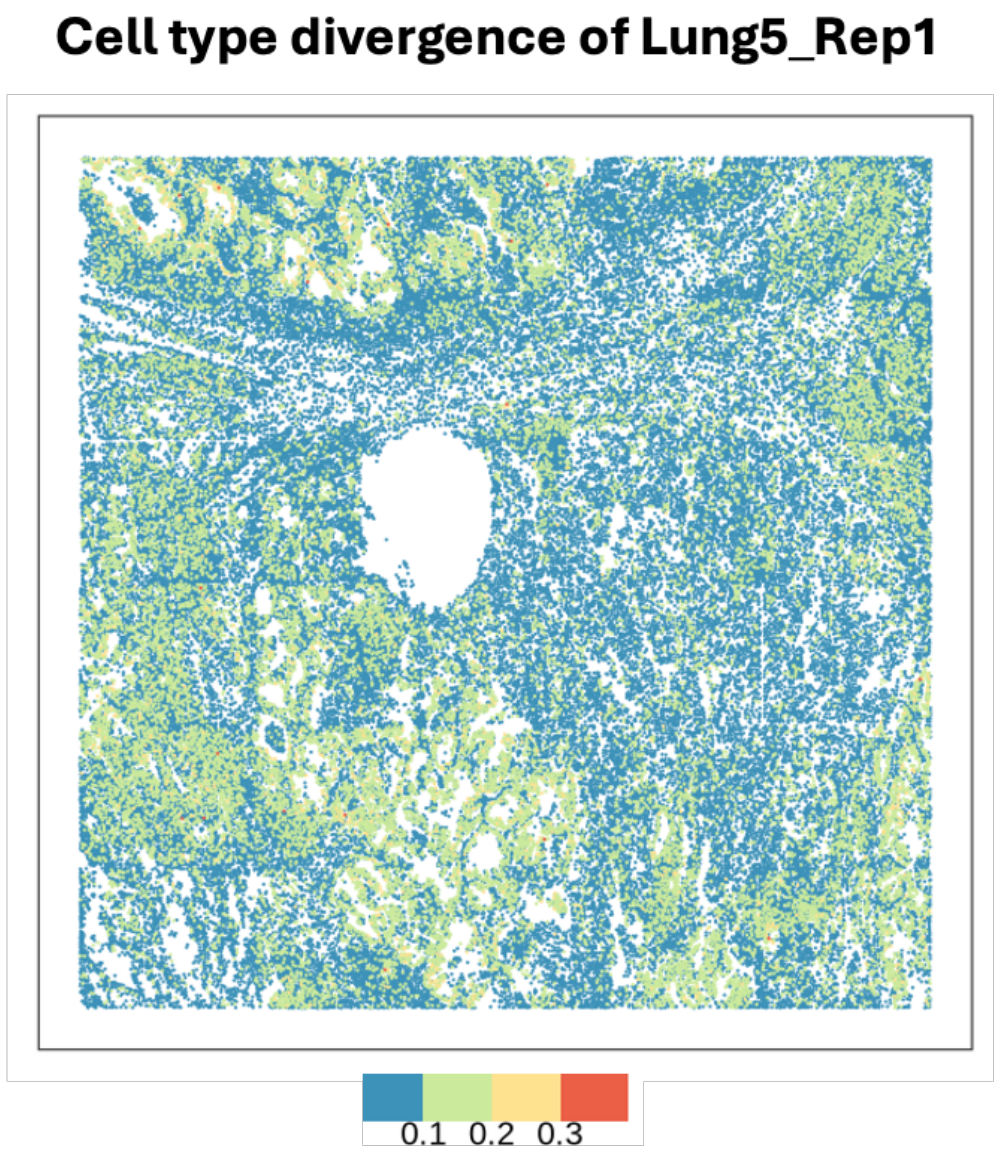


**Fig S6: Cell type divergence in the CosMx Lung5_Rep1 sample, related to Figure 3.** Cell type divergence of all segmented cells in Lung5_Rep1, computed based on their phenotype space embeddings derived using Φ-Space ST. The tumour and lymphoid structure domains in Fig 3A showed higher divergence.

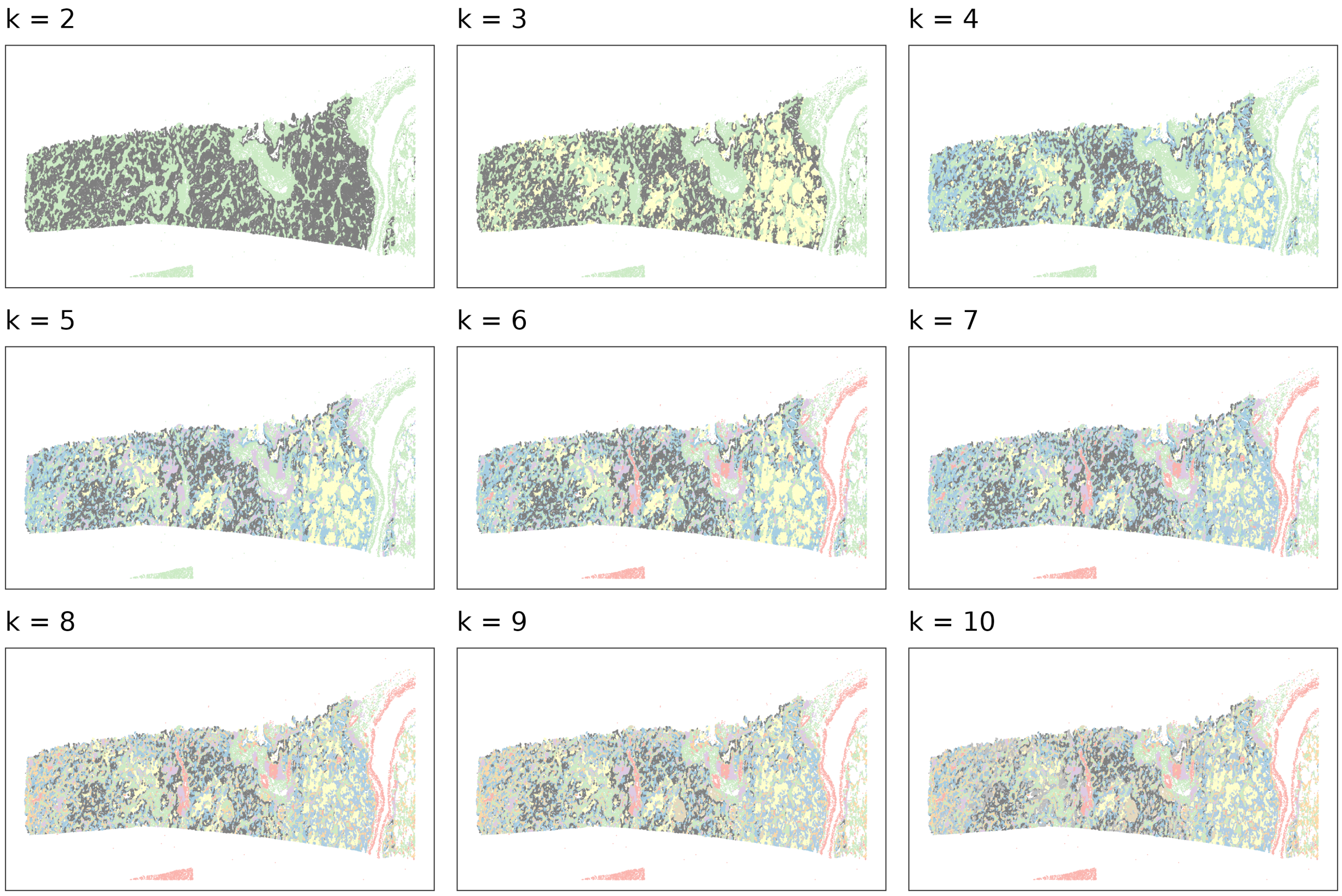


**Fig S7: Spatial niches for Xenium lung sample, related to Figure 4.** When identifying spatial niches for the Xenium lung sample, we applied k-means to spatially smoothed Φ-Space ST scores and varied the number of clusters from 2 to 10. Partition of the whole tissue section became virtually undistinguishable after number of clusters increased to 8. Hence we chose to present the 8-niche result in the main text.

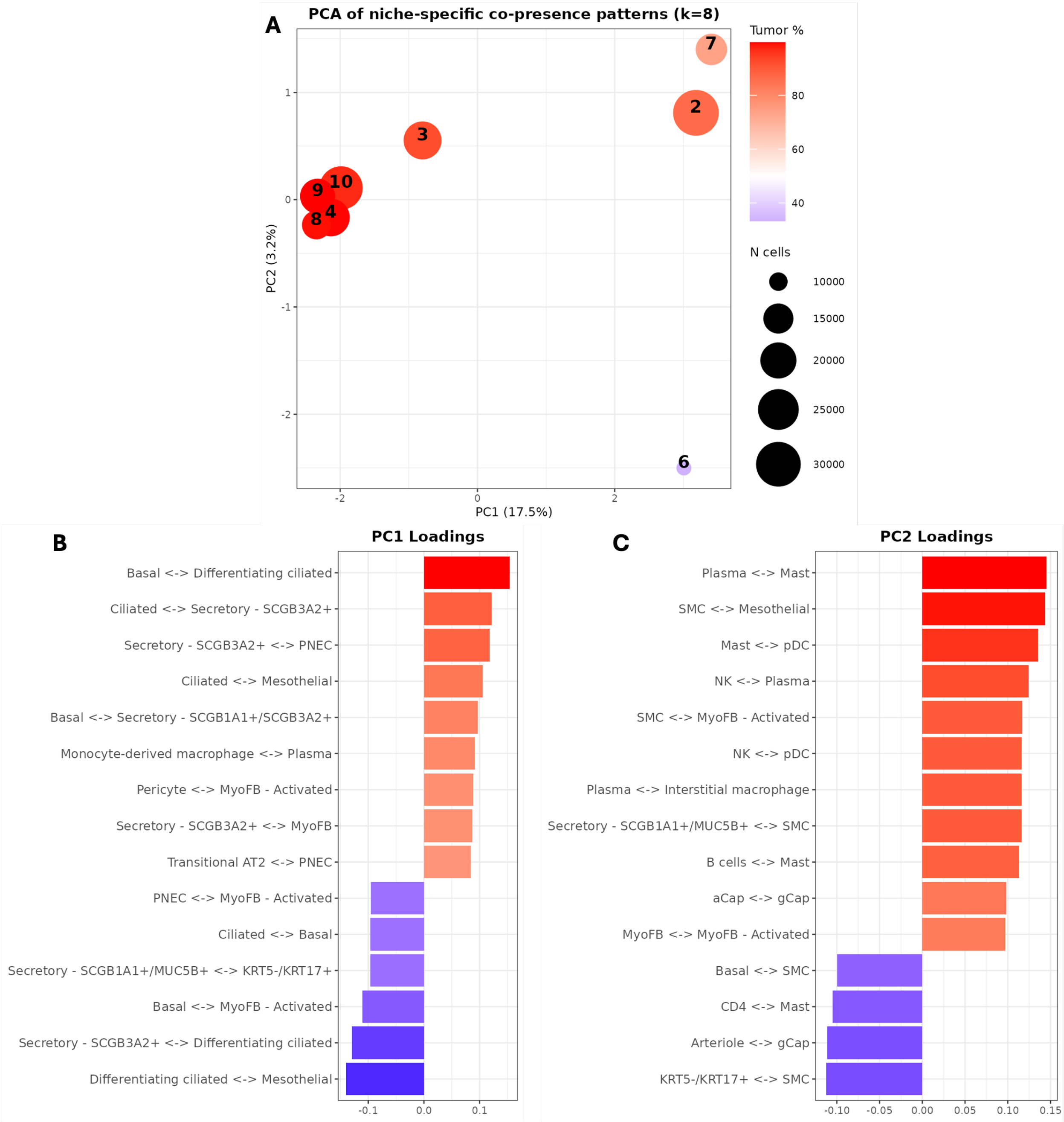


**Fig S8: Cell type co-presence analysis for Xenium NSCLC sample, related to Figure 4. A:** The first two principal components (PCs) of cell type co-presence matrices derived from individual niches in the Xenium NSCLC sample. We observed tumour high niches (3, 4, 8, 9, 10), intermediate niches (2, 7) and tumour low niche (6) occupying three corners on the PC plot. **B&C:** Top 15 loading values (absolute value) contributing to PC1 and PC2.

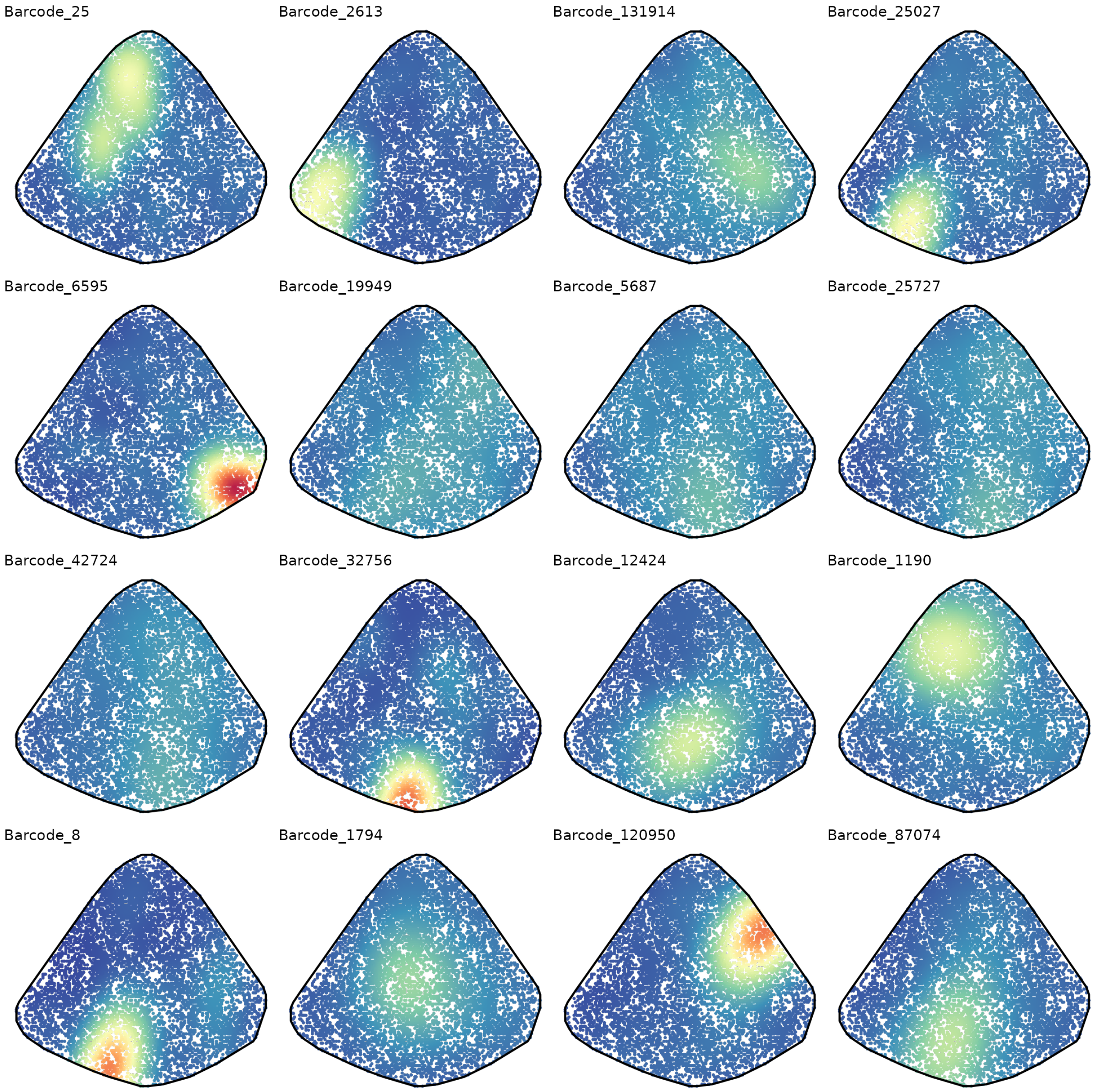


**Fig S9: Spatial distributions of most populous AML barcodes, related to Figure 5.** Each subfigure showed the spatial distribution of a specific AML barcode, where each spot represented a barcoded Stereo-seq bin. The spatial distribution of each barcode was estimated using a 2-dimensional kernel density estimator[2,3]. The warmer the colour was, the higher the estimated density value.

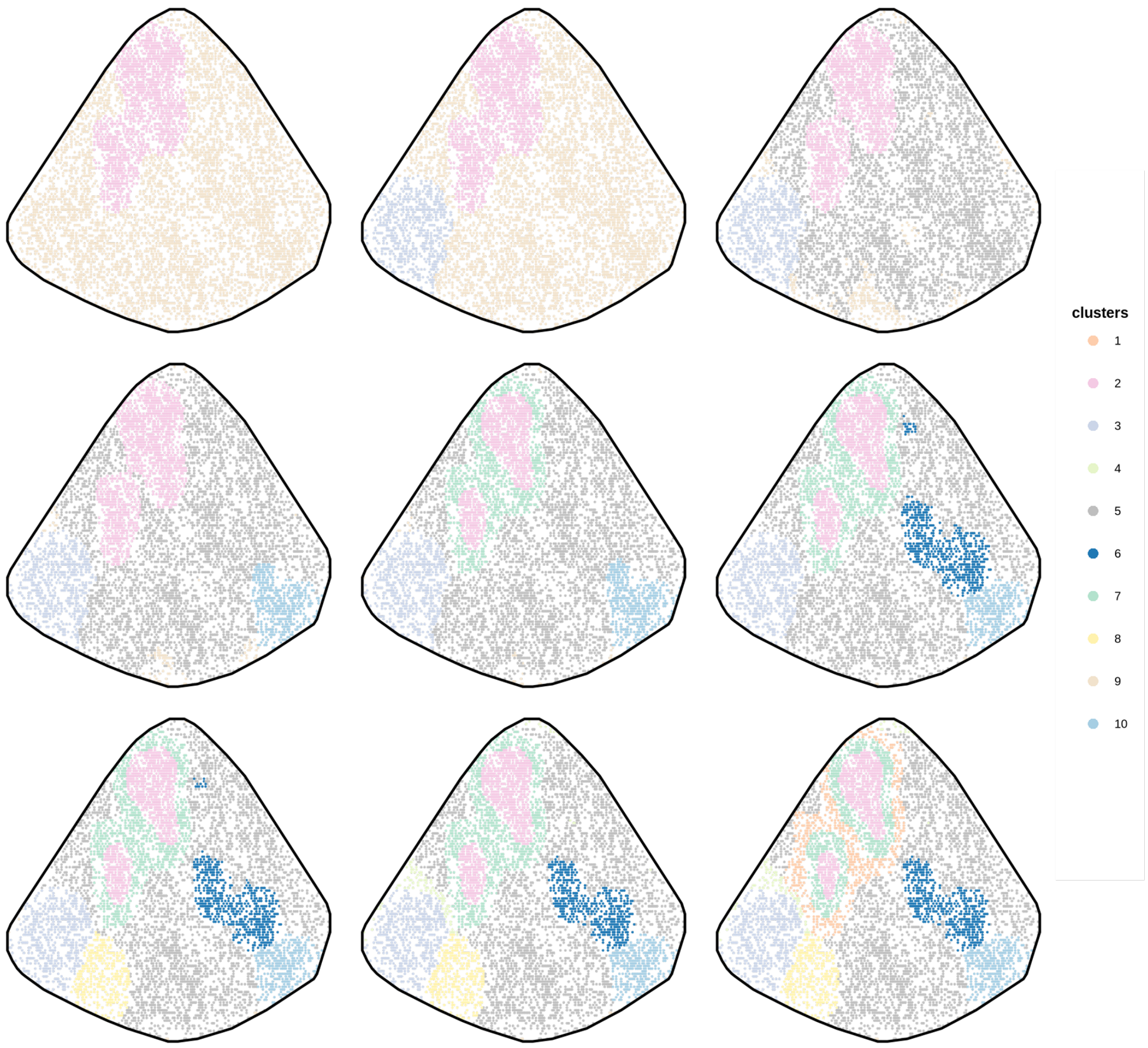


**Fig S10: Clustering Stereo-seq bins according to their barcode composition, related to Figure 5.** We varied the number of clusters $k$ from 2 to 10 in the meta-clone identification pipeline (see STAR Methods). Each subfigure above represented a specific choice of $k$.

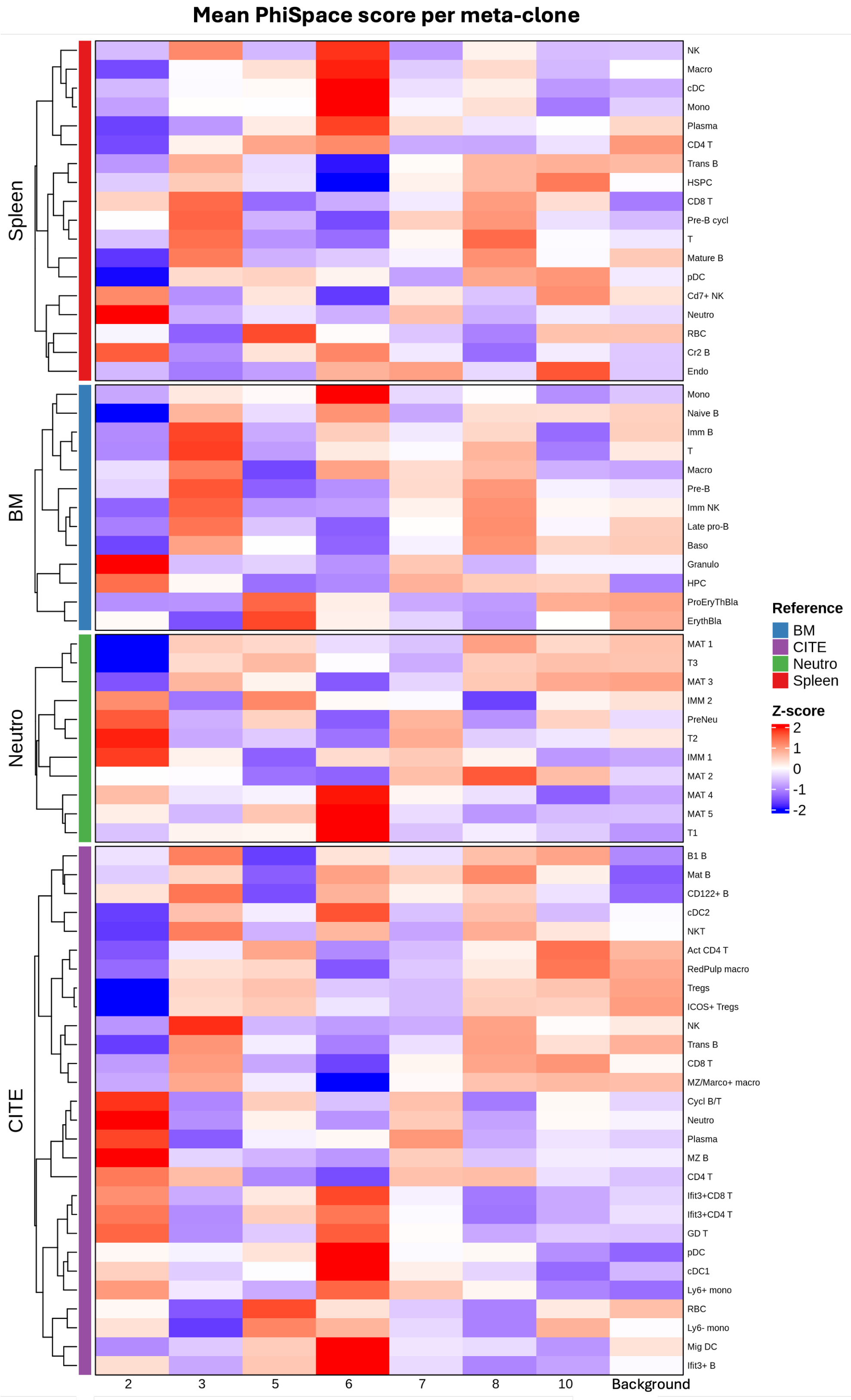


**Fig S11: Cell type composition in meta-clones, related to Figure 5.** Heatmap of mean Φ-Space scores ($z$-scaled) for each meta-clone and background non-barcoded bins. Cell types (rows) grouped by reference dataset.

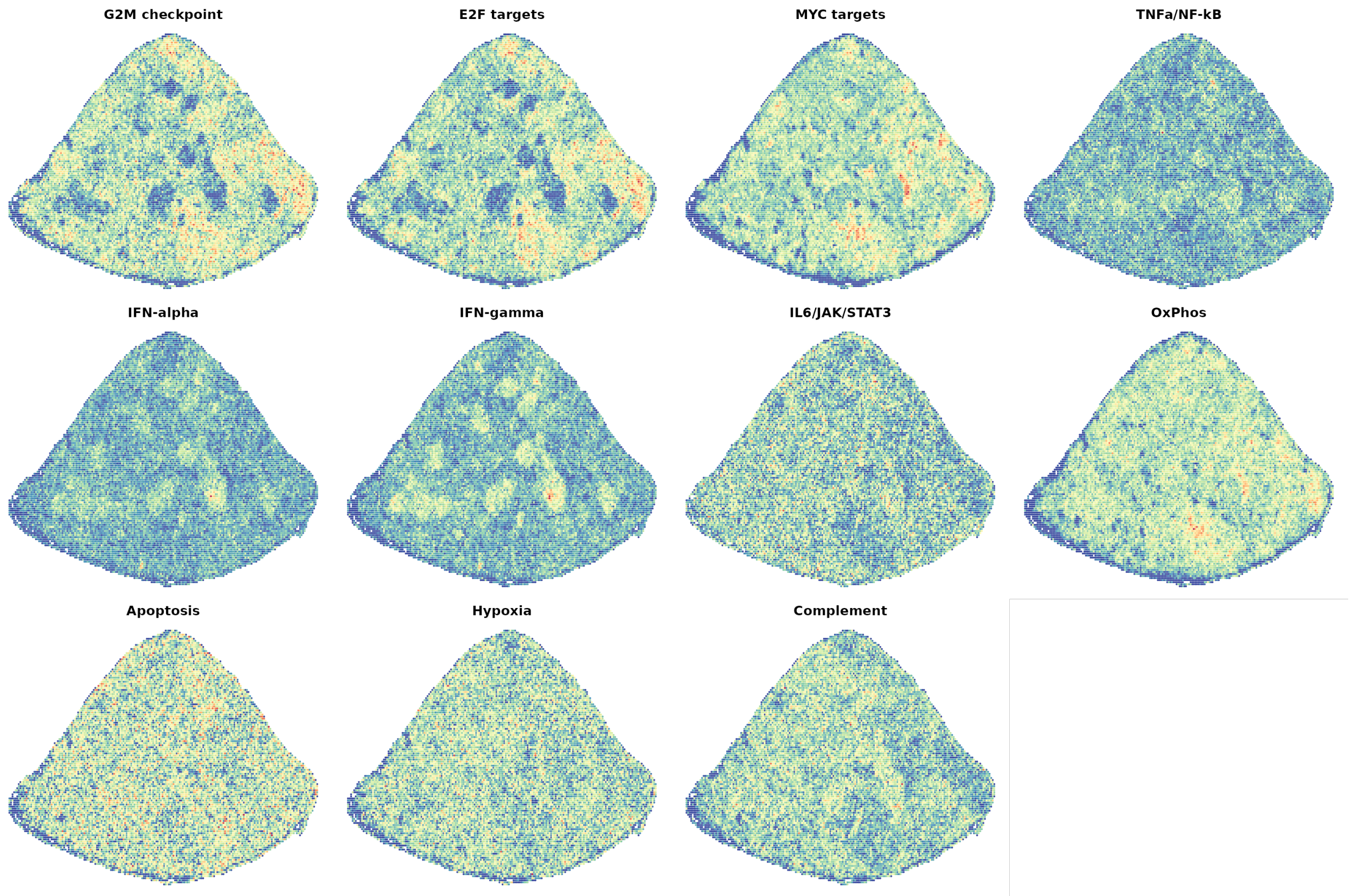


**Fig S12: Spatial distribution of AML gene signatures, related to Figure 5.** Spatial heatmaps of 11 Hallmark gene expression signatures scored using UCell.

## Table S1: Niche-specific enriched cell types in all lung samples in CosMx case study, related to Figure 3.

| | immune | lymphoid structure | macrophages | myeloid-enriched stroma | neutrophils | plasmablast-enriched stroma | stroma | tumor interior | tumor-stroma boundary |
|---|---|---|---|---|---|---|---|---|---|
| Lung5_Rep1 | Alveolar Mph CCL3+ | B cells | Non-classical monocytes | Alveolar Mph CCL3+ | Alveolar Mph CCL3+ | Plasma cells | Adventitial fibroblasts | AT1 | AT1 |
| | B cells | CD4 T cells | Alveolar Mph CCL3+ | Non-classical monocytes | EC aerocyte capillary | Adventitial fibroblasts | Smooth muscle | Basal resting | Multiciliated (non-nasal) |
| | CD8 T cells | EC venous systemic | Monocyte-derived Mph | EC aerocyte capillary | Goblet (nasal) | Club (nasal) | Non-classical monocytes | Multiciliated (non-nasal) | Suprabasal |
| | EC aerocyte capillary | CD8 T cells | EC aerocyte capillary | NK cells | NK cells | Club (non-nasal) | DC2 | Suprabasal | Basal resting |
| | CD4 T cells | Hillock-like | NK cells | EC general capillary | EC general capillary | Smooth muscle | Alveolar fibroblasts | Hillock-like | Smooth muscle |
| Lung5_Rep2 | CD4 T cells | B cells | | Alveolar Mph CCL3+ | Alveolar Mph CCL3+ | Plasma cells | Adventitial fibroblasts | AT1 | AT1 |
| | Suprabasal | CD4 T cells | | Non-classical monocytes | EC aerocyte capillary | Club (nasal) | Smooth muscle | Basal resting | Multiciliated (non-nasal) |
| | NK cells | EC venous systemic | | NK cells | NK cells | Adventitial fibroblasts | DC2 | Multiciliated (non-nasal) | Basal resting |
| | Alveolar Mph CCL3+ | CD8 T cells | | EC aerocyte capillary | Goblet (nasal) | EC general capillary | Lymphatic EC mature | Suprabasal | Suprabasal |
| | AT1 | EC venous pulmonary | | EC general capillary | AT1 | Alveolar fibroblasts | Alveolar macrophages | Hillock-like | Classical monocytes |
| Lung5_Rep3 | CD4 T cells | B cells | Alveolar Mph CCL3+ | Alveolar Mph CCL3+ | Alveolar Mph CCL3+ | Plasma cells | Adventitial fibroblasts | AT1 | Smooth muscle |
| | Non-classical monocytes | CD4 T cells | EC aerocyte capillary | EC aerocyte capillary | EC aerocyte capillary | Club (nasal) | Smooth muscle | Basal resting | AT1 |
| | Alveolar Mph CCL3+ | EC venous systemic | NK cells | Non-classical monocytes | NK cells | AT2 | DC2 | Multiciliated (non-nasal) | Multiciliated (non-nasal) |
| | NK cells | pre-TB secretory | CD4 T cells | NK cells | Non-classical monocytes | Adventitial fibroblasts | Non-classical monocytes | Hillock-like | Basal resting |
| | EC aerocyte capillary | CD8 T cells | Goblet (nasal) | EC general capillary | EC general capillary | Plasmacytoid DCs | Alveolar fibroblasts | Suprabasal | Hillock-like |
| Lung6 | Alveolar Mph CCL3+ | Alveolar Mph CCL3+ | Alveolar Mph CCL3+ | Alveolar Mph CCL3+ | | | Alveolar Mph CCL3+ | Hillock-like | CD4 T cells |
| | Interstitial Mph perivascular | Plasma cells | Interstitial Mph perivascular | Interstitial Mph perivascular | | | Plasma cells | NK cells | Alveolar Mph CCL3+ |
| | Plasma cells | Interstitial Mph perivascular | Alveolar macrophages | DC2 | | | Interstitial Mph perivascular | Suprabasal | Classical monocytes |
| | DC2 | CD4 T cells | DC2 | AT2 | | | CD4 T cells | Basal resting | Lymphatic EC mature |
| | CD4 T cells | B cells | Monocyte-derived Mph | Plasma cells | | | Lymphatic EC mature | Goblet (nasal) | Plasma cells |
| Lung9_Rep1 | EC venous pulmonary | CD4 T cells | Monocyte-derived Mph | EC venous pulmonary | Monocyte-derived Mph | Plasma cells | EC venous pulmonary | pre-TB secretory | Adventitial fibroblasts |
| | Plasma cells | Plasma cells | Non-classical monocytes | EC arterial | EC venous pulmonary | B cells | EC aerocyte capillary | Neuroendocrine | Alveolar fibroblasts |
| | CD4 T cells | EC venous pulmonary | EC venous pulmonary | Non-classical monocytes | CD4 T cells | Smooth muscle | SMG duct | Deuterosomal | Smooth muscle |
| | EC aerocyte capillary | EC aerocyte capillary | EC arterial | SMG duct | Basal resting | EC venous pulmonary | EC general capillary | Tuft | Club (non-nasal) |
| | Alveolar fibroblasts | Lymphatic EC mature | SMG duct | EC aerocyte capillary | AT2 | Multiciliated (non-nasal) | Alveolar Mph CCL3+ | Hillock-like | EC general capillary |
| Lung9_Rep2 | Alveolar fibroblasts | Alveolar fibroblasts | | Alveolar fibroblasts | Non-classical monocytes | Plasma cells | Alveolar fibroblasts | pre-TB secretory | Alveolar fibroblasts |
| | Plasma cells | Plasma cells | | DC2 | DC2 | Alveolar fibroblasts | Adventitial fibroblasts | Neuroendocrine | Plasma cells |
| | Non-classical monocytes | Non-classical monocytes | | Non-classical monocytes | Alveolar fibroblasts | Non-classical monocytes | DC2 | Hillock-like | Adventitial fibroblasts |
| | Adventitial fibroblasts | Adventitial fibroblasts | | Adventitial fibroblasts | Alveolar Mph CCL3+ | Adventitial fibroblasts | Smooth muscle | Deuterosomal | EC general capillary |
| | EC general capillary | DC2 | | Multiciliated (nasal) | Multiciliated (nasal) | Smooth muscle | Alveolar Mph CCL3+ | Tuft | Alveolar macrophages |
| Lung12 | Monocyte-derived Mph | Plasma cells | | Plasma cells | CD4 T cells | Plasma cells | Adventitial fibroblasts | Goblet (nasal) | AT1 |
| | Suprabasal | B cells | | CD4 T cells | NK cells | B cells | Alveolar fibroblasts | AT1 | Club (nasal) |
| | EC arterial | Monocyte-derived Mph | | NK cells | CD8 T cells | EC venous pulmonary | EC general capillary | Classical monocytes | AT2 |
| | NK cells | EC venous pulmonary | | Non-classical monocytes | Non-classical monocytes | EC arterial | Alveolar macrophages | Monocyte-derived Mph | Classical monocytes |
| | CD8 T cells | EC arterial | | EC venous systemic | DC2 | Lymphatic EC mature | Smooth muscle | Basal resting | Smooth muscle |
| Lung13 | Interstitial Mph perivascular | | Monocyte-derived Mph | Monocyte-derived Mph | Club (nasal) | | Adventitial fibroblasts | pre-TB secretory | AT2 |
| | Plasma cells | | Non-classical monocytes | pre-TB secretory | pre-TB secretory | | Suprabasal | Club (nasal) | Goblet (nasal) |
| | DC2 | | Club (non-nasal) | Suprabasal | Multiciliated (non-nasal) | | EC general capillary | Goblet (nasal) | AT1 |
| | EC general capillary | | Alveolar fibroblasts | CD8 T cells | CD4 T cells | | Alveolar fibroblasts | EC arterial | Classical monocytes |
| | CD8 T cells | | Pericytes | EC arterial | Suprabasal | | Interstitial Mph perivascular | AT1 | Club (nasal) |

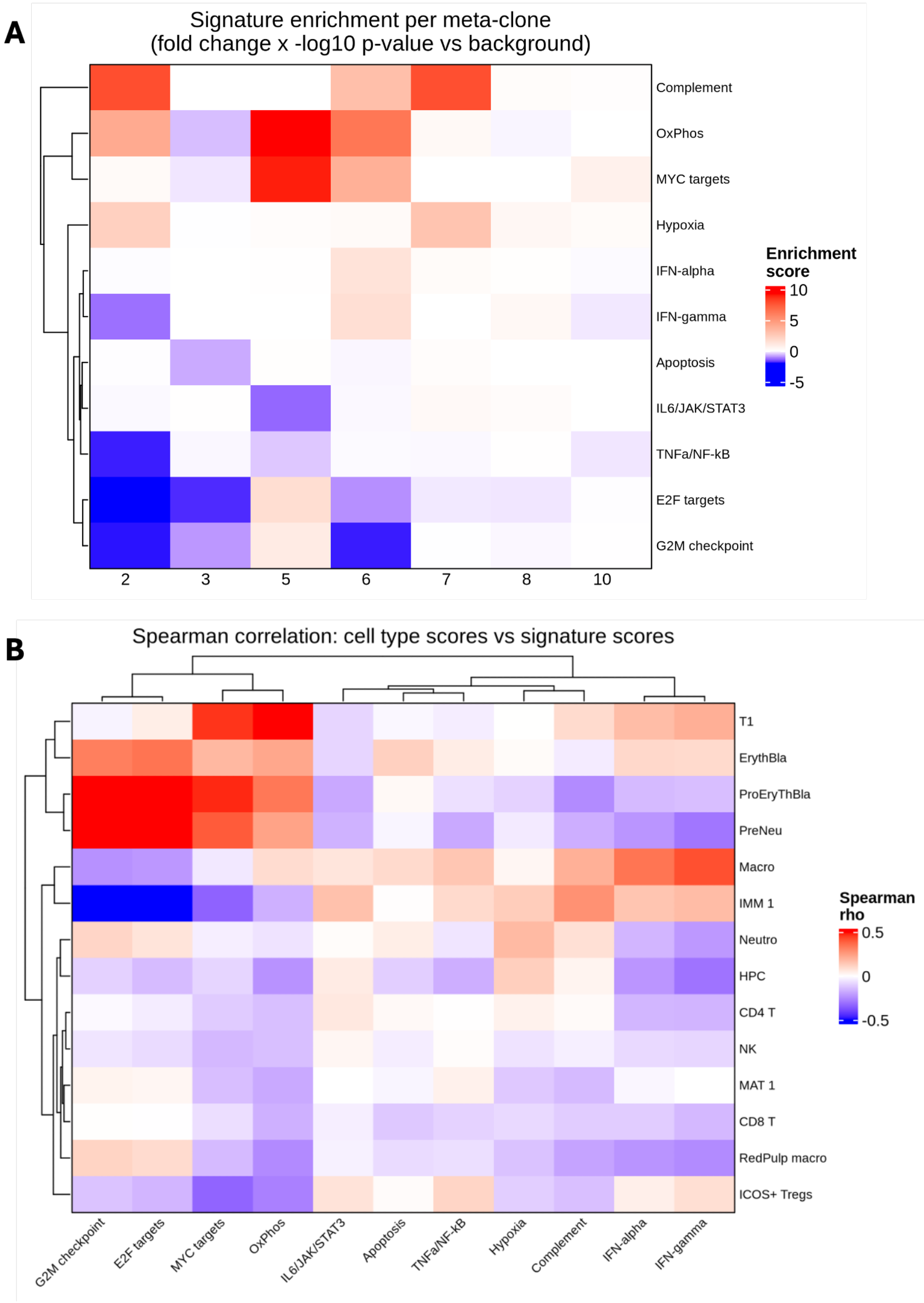


**Fig S13: Enrichment of AML gene signatures in meta-clones, related to Figure 5. A:** Signature enrichment per meta-clone: enrichment score = fold change $\times -\log_{10}$ $p$-value from Wilcoxon test vs background. **B:** Spearman correlations between selected cell type scores and signature scores, linking cell type identity to cellular state.

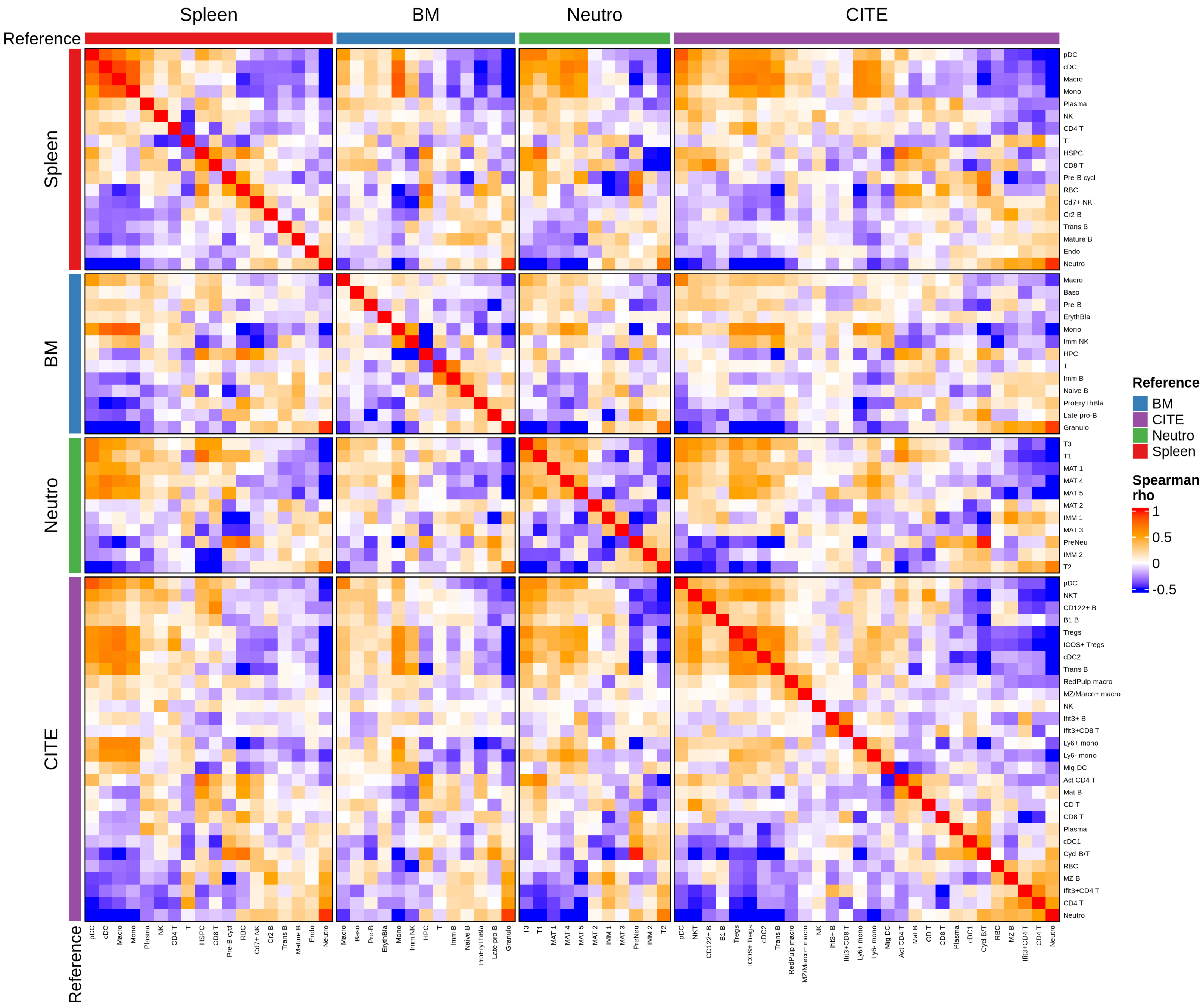


**Fig S14: Correlation between cell type scores from multiple references, related to Figure 5.** Spearman correlation heatmap of all 70 cell type scores across the 4 references (Spleen, BM, Neutro, CITE), computed on the intermediate scRNA-seq data (5,595 cells).

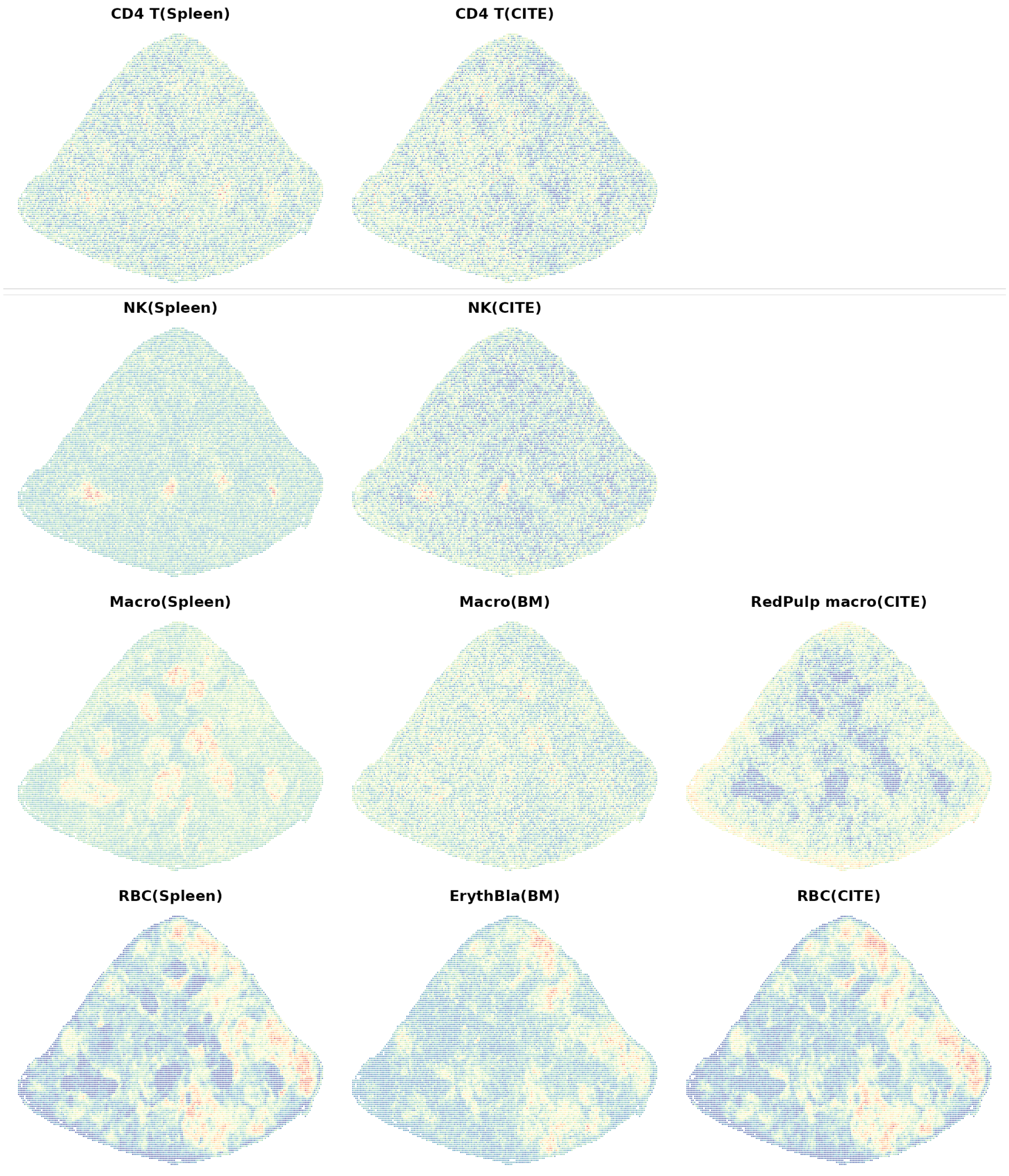


**Fig S15: Spatial distribution of overlapping cell type groups, related to Figure 5.** Side-by-side spatial heatmaps of Φ-Space scores for 4 representative overlapping cell type groups (CD4 T, NK, Macrophage, Erythrocyte) in the Stereo-seq data. Within each row, the same colour scale is used across references.

**Table S2: Cross-reference Spearman correlations of Φ-Space scores, related to Figure 5.** Spearman correlations between overlapping cell type scores across references, computed on the intermediate scRNA-seq data (5,595 cells). Selected pairs are shown; see Supplementary Fig S14 for all 29 pairs.

| Cell type pair | References | Spearman $\rho$ |
|---|---|---|
| Neutrophil | Spleen vs CITE | 0.94 |
| pDC | Spleen vs CITE | 0.83 |
| Monocyte | Spleen vs BM | 0.81 |
| cDC / cDC2 | Spleen vs CITE | 0.67 |
| Monocyte / Ly6$^+$ mono | Spleen vs CITE | 0.63 |
| Monocyte / Ly6$^+$ mono | BM vs CITE | 0.62 |
| Macrophage | Spleen vs BM | 0.38 |
| NK | Spleen vs CITE | 0.38 |
| Mature B / Mat B | Spleen vs CITE | 0.22 |
| CD4 T | Spleen vs CITE | −0.38 |
| cDC1 / cDC2 (control) | CITE vs CITE | −0.42 |
