# Supplementary material for: Φ-Space ST: A platform-agnostic method to identify cell states in spatial transcriptomics studies
Source: Cell Rep Methods. 2026 Jun 12;6(8):101483. doi: 10.1016/j.crmeth.2026.101483 (PMC13494538; doi:10.1016/j.crmeth.2026.101483)
Supplement: Supplementary file 2 [file mmc2.pdf]

Cell Reports Methods

Article

Φ-Space ST: A platform-agnostic method to identify cell states in spatial transcriptomics studies

Graphical abstract

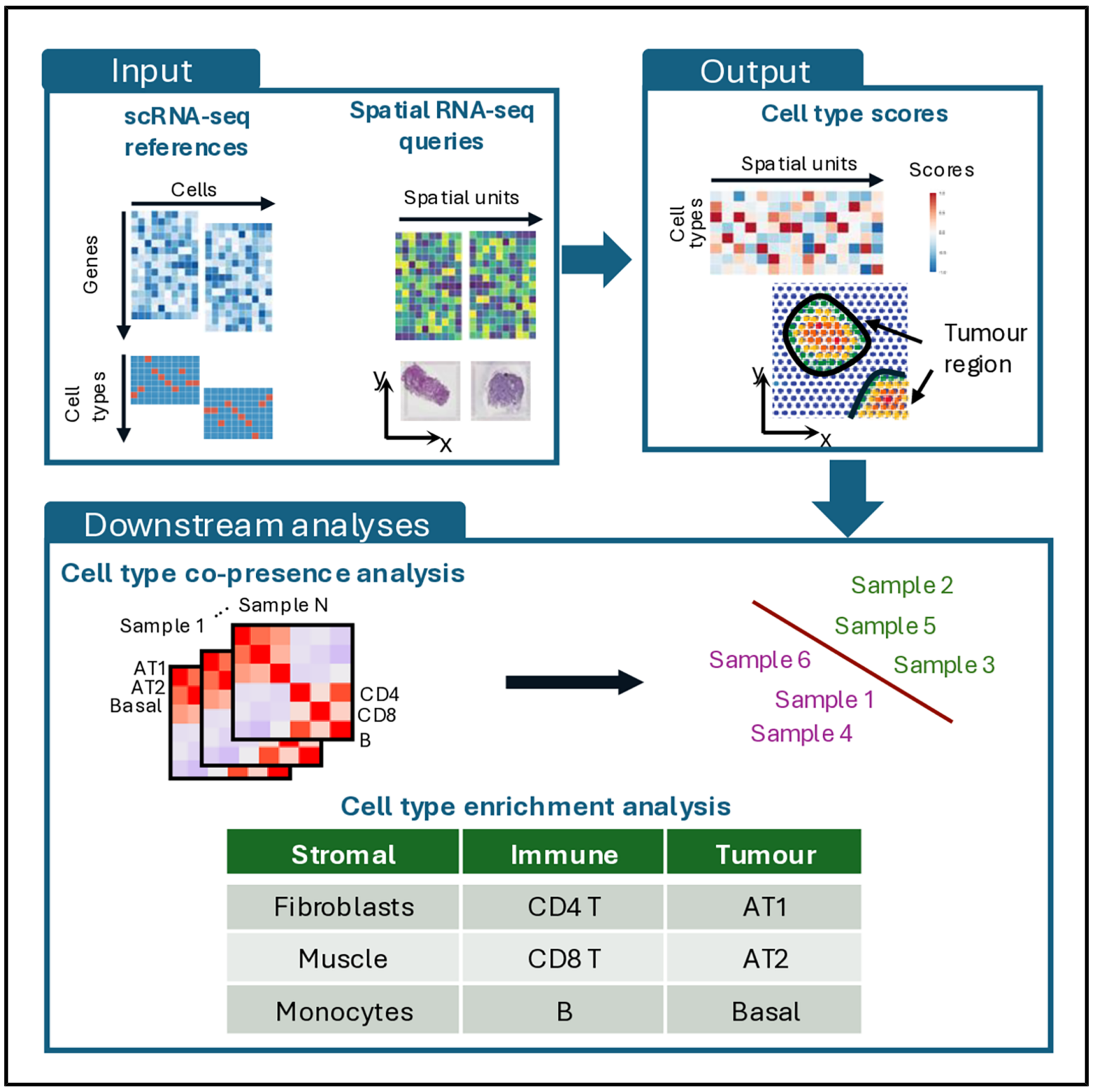

Authors

Jiadong Mao, Jarny Choi, Kim-Anh Lê Cao

Correspondence

kimanh.lecao@unimelb.edu.au

In brief

Mao et al. present Φ-Space ST, a platform-agnostic method for annotating continuous cell states in spatial transcriptomics using multiple scRNA-seq references. Validated across Visium, CosMx, Xenium, and Stereo-seq cancer datasets, Φ-Space ST reveals niche-specific cell-type co-presence patterns and clonal heterogeneity without requiring cell segmentation.

Highlights

- Φ-Space ST annotates cell states across diverse spatial transcriptomics (ST) platforms
- Multiple scRNA-seq references are harmonized for robust spatial annotation
- Segmentation-free workflow enables interpretable niche identification
- AML meta-clones show spatially organized and phenotypically distinct cell states

Mao et al., 2026, Cell Reports Methods 6, 101483
August 17, 2026 © 2026 The Author(s). Published by Elsevier Inc.
https://doi.org/10.1016/j.crmeth.2026.101483

CellPress

Cell Reports Methods

CellPress
OPEN ACCESS

Article

Φ-Space ST: A platform-agnostic method to identify cell states in spatial transcriptomics studies

Jiadong Mao,[1,3] Jarny Choi,[2,3] and Kim-Anh Lê Cao[1,4,*]
[1]Melbourne Integrative Genomics, School of Mathematics and Statistics, The University of Melbourne, Parkville, VIC 3010, Australia
[2]Bioinformatics and Cellular Genomics, St Vincent's Institute of Medical Research, Fitzroy, VIC 3065, Australia
[3]These authors contributed equally
[4]Lead contact
*Correspondence: kimanh.lecao@unimelb.edu.au
https://doi.org/10.1016/j.crmeth.2026.101483

MOTIVATION Cell-type annotation of spatial transcriptomics (ST) data is critical for understanding tissue organization and disease mechanisms, yet existing methods are limited by reliance on single references, restrictive parametric assumptions, and high computational cost. These limitations become especially problematic in complex disease settings, such as cancer, where cells exhibit transitional and hybrid states not represented in any single reference. There is a pressing need for a unified, platform-agnostic framework that can leverage multiple scRNA-seq references to annotate continuous cell states across diverse ST platforms and resolutions.

SUMMARY

We introduce Φ-Space ST, a platform-agnostic method to identify continuous cell states in spatial transcriptomics (ST) data using multiple scRNA-seq references. For ST with supercellular resolution, Φ-Space ST achieves interpretable cell-type deconvolution with significantly faster computation. For subcellular resolution, Φ-Space ST annotates cell states without cell segmentation, leading to highly insightful spatial niche identification. Φ-Space ST harmonizes annotations derived from multiple scRNA-seq references and provides interpretable characterizations of disease cell states by leveraging healthy references. We validate Φ-Space ST in four case studies involving CosMx, Visium, Xenium, and Stereo-seq platforms for various cancer tissues. Our method revealed niche-specific enriched cell types and distinct cell-type co-presence patterns that distinguish tumor from non-tumor tissue regions. These findings highlight the potential of Φ-Space ST as a robust and scalable tool for ST data analysis for understanding complex tissues and pathologies.

INTRODUCTION

The rapid advancement of spatial transcriptomics (ST) technologies has transformed our ability to explore the spatial organisation of gene expression within tissues, offering unprecedented insights into cellular architecture and tissue microenvironments.[1] Technologies such as 10x Genomics Visium[2] and Xenium,[3] NanoString CosMx[4] and BGI's Stereo-seq[5] have allowed researchers to map gene expression at various spatial resolutions, ranging from subcellular to supercellular levels. These advances have unlocked opportunities to understand tissue organization and cellular interactions in both healthy and disease states. However, achieving biologically meaningful annotations of continuous cell states remains a major hurdle, particularly across platforms with varying resolutions and technical specifications.

A common strategy for cell-type annotation in ST data involves comparing expression profiles of query ST data to those in well-annotated scRNA-seq ref.[6,7] However, existing ST platforms lack exact single-cell resolution since they do not directly measure single cells.[1,8] For supercellular resolution ST such as Visium,[2] each measurement unit is a multi-cellular spot and hence may contain a mixture of cell identities. For subcellular resolution ST such as CosMx[4] and Stereo-seq,[5] the minimal measurement unit is often much smaller than the size of a single cell. These subcellular measurement units can be aggregated into suitable sizes via simple spatial binning or sophisticated cell segmentation algorithms.[9,10] However, the resulting bins or segmented cells may still contain fractions of transcripts from multiple neighboring cells.[10,11]

To address the mixing of cell identities commonly seen in both supercellular and subcellular ST data, cell-type deconvolution

© 2026 The Author(s). Published by Elsevier Inc.
This is an open access article under the CC BY license (http://creativecommons.org/licenses/by/4.0/).

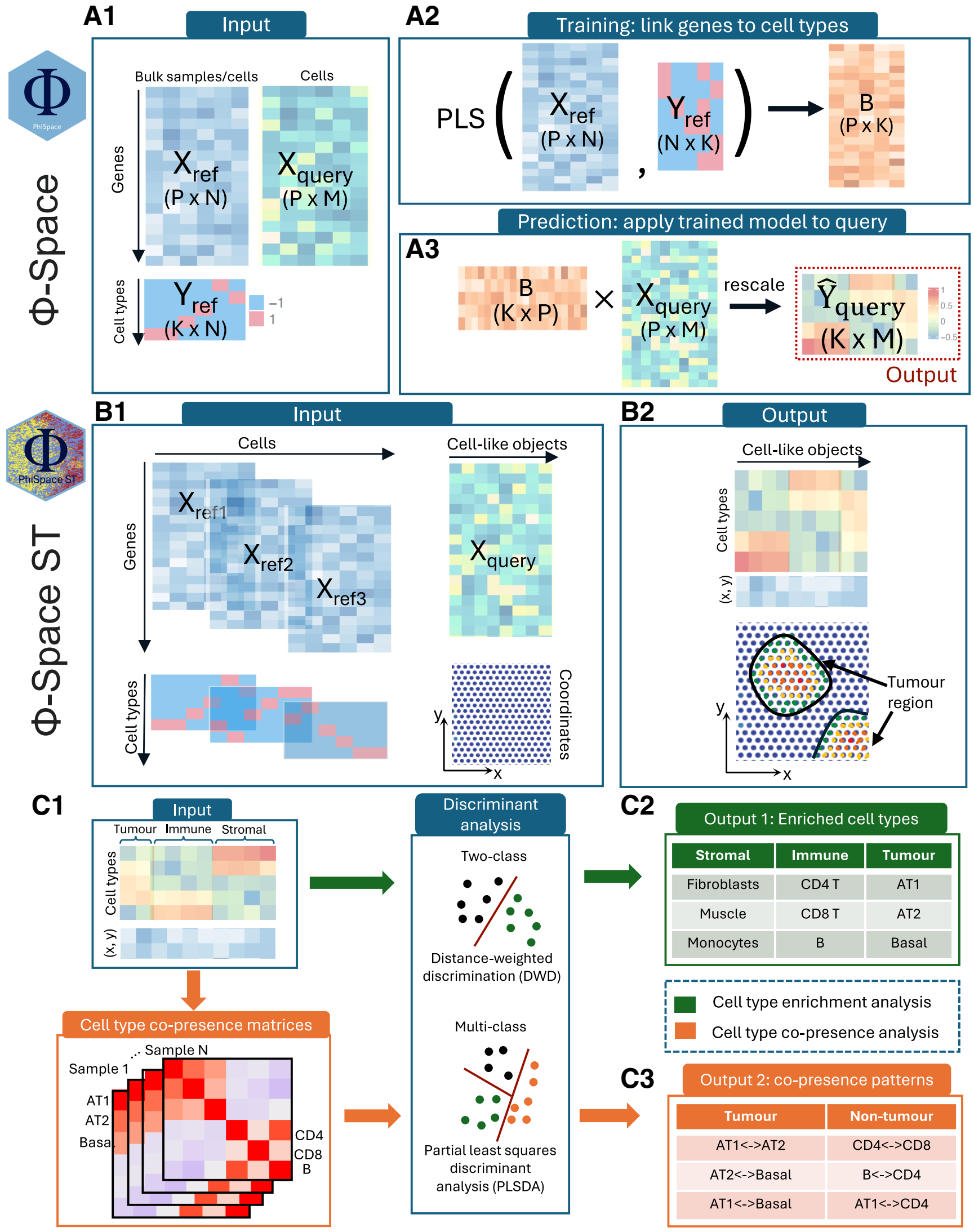

Figure 1. Schema of Φ-Space ST

(A) Framework of the transcriptomic module of our previous Φ-Space method.[20] (A1) Φ-Space enables continuous phenotyping of cells based on either bulk or single-cell RNA-seq references. (A2) We first compute a PLS regression coefficient matrix B to predict all K cell types on a continuous scale using P gene expression levels. (A3) We then apply B to query gene expression matrix to obtain continuous cell-type scores $\hat{Y}_{\text{query}}$ for each of the query cells.

(B) Framework of Φ-Space ST, a platform-agnostic and computationally efficient method designed to identify continuous cell states of spatial cell-like objects (supercellular spots, segmented cells, or spatial bins) using ST data. (B1) We first train PLS regression models from one or multiple scRNA-seq references to continuously predict reference cell types. (B2) We then apply the PLS models to the query ST data. Φ-Space ST accurately identifies complex, e.g., transitional and malignant, cell states.

(C) Analyses based on Φ-Space ST continuous annotations of query cell-like objects, which we refer to as their *phenotype space embeddings*. (C1) We identify spatial niches by clustering the phenotype space embeddings. (C2) Cell-type enrichment analysis, where we identify differentially enriched cell types in each spatial niche. (C3) Cell-type co-presence analysis, where we quantify the spatial co-presence of cell types and then use discriminant analysis to infer how specific co-presence patterns are associated with disease conditions.

methods have been developed.[12–17] These deconvolution methods can estimate cell-type abundances within each spatial measurement unit such as supercellular spots, segmented cells and spatial bins based on reference scRNA-seq datasets. In the remainder of this article, we will refer to these spatial measurements as *cell-like objects*. Existing cell-type deconvolution methods show considerable promise but face several limitations, including over-reliance on single references and restrictive parametric assumptions about the data-generating process.[12,14,16,17] These limitations are especially problematic for complex and dynamic tissue environments, such as cancer, where cells frequently exhibit transitional or hybrid states that are not represented in reference datasets. In addition, existing methods are often computationally inefficient and difficult to extend for spatial omics data beyond transcriptomics, such as spatial proteomics and epigenomics.[18,19]

To address these challenges, we present Φ-Space ST, a computational framework designed for platform-agnostic annotation of cell states in ST data. Φ-Space ST builds on Φ-Space, previously developed for single-cell multiomics analysis[20] (Figure 1A). Φ-Space ST leverages partial least squares (PLS) regression to annotate spatial cell-like objects with continuous cell-type scores. Unlike existing deconvolution methods, Φ-Space ST enables annotation using multiple scRNA-seq references, allowing the identification of complex cell states. Its nonparametric and flexible framework avoids restrictive assumptions, offering greater adaptability. Additionally, Φ-Space ST is significantly faster than state-of-the-art methods like RCTD[12] and cell2location.[14] These features make Φ-Space ST highly versatile for a wide range of ST platforms and biological contexts.

Recent developments in ST analysis have expanded beyond cell-type annotation to address a range of complementary tasks, including multi-sample integration,[21] spatial niche identification,[22,23] and spatially resolved variant cataloging.[24] Several of these tasks, such as cross-sample integration and spatial niche identification, can also be performed as downstream analyses of Φ-Space ST's continuous cell-type scores. Because Φ-Space ST is built on the simple and flexible framework of PLS regression, its output scores can be readily adapted to diverse analytical goals, without requiring purpose-built models for each task.

CellPress
OPEN ACCESS

This versatility positions Φ-Space ST as a foundation for ST workflows, complementing the specialized tools emerging in this rapidly growing field.

In this study, we illustrate the application of Φ-Space ST to four cancer studies involving diverse ST platforms including 10x Genomics Visium[2] and Xenium,[3] NanoString CosMx[4] and BGI's Stereo-seq.[5] We demonstrate the ability of Φ-Space ST in identifying spatial niches, enriched cell types and cell-type co-presence patterns that distinguish tumor from non-tumor tissue regions. By offering robust, scalable and biologically interpretable annotations, Φ-Space ST sets the stage for more comprehensive analyses of spatially resolved transcriptomics data.

RESULTS

Φ-Space ST: A platform-agnostic method for identifying complex cell states from ST data

Overview

Φ-Space ST is a platform-agnostic method for annotating different types of *cell-like objects* in ST data. We define a cell-like object as the collection of transcripts observed in the proximity of a certain tissue location. Depending on the technical specifications of ST platforms, cell-like objects can be multicellular spots (e.g., 10x Visium[2]), segmented cells,[9] or bins of aggregated transcripts (e.g., Stereo-seq[5]).

Multi-reference learning of cell states

Φ-Space ST transfers the rich knowledge about cell states from multiple reference scRNA-seq datasets to the query ST data, based on the assumption that all cell states in the query data can be characterized as mixtures of cell types defined in the reference, including out-of-reference cell states that may be present in the query. For example, some lung cancer cells may be jointly characterized by healthy lung epithelial and mesenchymal cell types.[25]

PLS as methodological backbone

The core of Φ-Space ST is PLS regression.[26,27] The first step of the Φ-Space ST workflow is to select genes that are useful for predicting cell types defined in the reference datasets. In the second step (Figure 1B1), a PLS regression model is trained from each reference scRNA-seq dataset to predict the reference cell type on a continuous scale. In the third step (Figure 1B2), the PLS models are applied to the query ST dataset. Each query cell-like object is characterized by its similarities with individual cell types defined in all references. We refer to this continuous annotation of cell-like objects as their *phenotype space embeddings*.

Quantifying cell-type divergence in complex tissue microenvironment

Conventional cell-type deconvolution methods characterize the cell state of a cell-like object as a *composition* of cell types in the reference, which requires the predicted cell-type scores of each cell-like object to sum up to 1.[12,14–17] While this is a reasonable assumption in some scenarios, it may become stringent in disease samples such as the tumor microenvironment, where tumor and tumor-related cells typically exhibit divergent cell states.[28] The restriction of cell-type scores to sum up to 1 can introduce compositional bias, which may hinder biologically meaningful comparison of cell states across tissue regions and samples (e.g., the difference between transcriptionally active and inactive tissue regions may be masked).[14]

Considering the complex cell-state landscape of disease-altered tissue microenvironment, Φ-Space ST removes the assumption that the query cell state can be characterized as a composition of reference cell types. As a result, we can measure the *cell-state divergence* of a query cell-like object, defined as the 75% quantile of all predicted cell-type scores. Higher cell-state divergence implies that a cell-like object exhibits similarity to more reference cell types, flagging stronger transcriptional activity or transitional cell states.

Advantage of Φ-Space ST scores compared to proportions

In addition to avoiding compositional bias (described above), Φ-Space ST continuous scores can all be low when a cell-like object does not match any reference cell type, or multiple scores can be simultaneously high for transitional or hybrid cell states.[20] Furthermore, the cell-state divergence measure is intrinsically undefined for proportion-based methods where the total is always 1.

When proportion-based output is desired, Φ-Space ST's continuous scores can be converted to proportions using the `Score2Prob()` function in the `PhiSpace` R package.[29] We benchmarked the resulting proportions against six representative deconvolution methods on 32 simulated Visium datasets[15] (STAR Methods; Figure S1A), confirming competitive deconvolution accuracy despite Φ-Space ST not being primarily designed for inferring proportions. Φ-Space ST is also among the fastest methods (median 11.9 s), approximately 25 times faster than RCTD and 80 times faster than Cell2location (Figure S1B).

Phenotype space embeddings for multi-sample biological discovery

The phenotype space embeddings of the cell-like objects from different tissue samples are in the same scale and hence provide a basis for multi-sample biological discoveries from ST data. We use phenotype space embeddings of cell-like objects to identify spatial niches, niche-specific enriched cell types and cell-type co-presence patterns that separate disease from healthy samples (Figure 1C; details in STAR Methods).

Summary of case studies

We apply Φ-Space ST to four cancer studies to showcase the biological insight provided by Φ-Space ST for identifying spatial phenotypic patterns in tumor microenvironments based on data from a diverse range of ST platforms. The case studies are summarized in Table 1 along with the annotation challenges faced for each of these platforms.

Φ-Space ST provides highly interpretable cell-type deconvolution for understanding cell-type co-presence

For supercellular spatial platforms such as 10x Visium, single-cell resolution is technically unattainable (see Table 1). As a result, to infer spatial distribution of cell types, deconvolution algorithms must be used to estimate the abundance of different cell types within each multicellular spot. In this case study, we first demonstrate that Φ-Space ST provides more interpretable cell-type deconvolution compared to some state-of-the-art deconvolution methods and is very fast to compute. We then use the Φ-Space ST deconvolution results to infer cell-type

Table 1. Summary of case studies and associated analytical challenges

Platform	Reference	Query	Challenges
10x Visium: supercellular sequencing-based (whole-transcriptome)	subsample (N = 58,759) of Human Lung Cell Atlas with 61 healthy cell types (Sikkema et al.[30])	eighteen Visium tissue samples from NSCLC patients and healthy donors (De Zuani et al.[31]) *cell-like objects*: multicellular spots (diameter 55 μm)	1, 3
NanoString CosMx: subcellular imaging-based (using a gene panel of ~980 genes)	four scRNA-seq datasets from healthy and fibrotic lungs, each including cells from one of the following lineages: immune, epithelial, endothelial, and mesenchymal (Natri et al.[32])	eight CosMx tissue samples from healthy and NSCLC donors (He et al.[33]) *cell-like objects*: segmented cells	1, 2
10x Xenium: subcellular imaging-based (gene panel of ~377 genes)	four scRNA-seq datasets as in the CosMx case	one Xenium FFPE NSCLC sample, 161,894 cells (Natri et al.[32]) *cell-like objects*: segmented cells	1, 2
BGI Stereo-seq: subcellular sequencing-based (whole-transcriptome)	four scRNA-seq datasets: spleen, mouse spleen (Zhang et al.[11]; Zhang et al.[34]); BM, mouse bone marrow (Harris et al.[35]); Neutro, neutrophils from healthy and cancerous mice (Ng et al. [36]); CITE, CITE-seq (only used the RNA part) of mouse spleen immune cells (Gayoso et al.[37])	one Stereo-seq mouse spleen sample from an AML mouse with cellular lineage tracing barcodes *cell-like objects*: spatial bins ("bin50", i.e., sidelength 25 μm)	1–4

Challenge 1: annotating tumor-associated cell states using healthy cell states; challenge 2: leveraging multiple scRNA-seq references; challenge 3: providing interpretable cell-type deconvolution in tumor microenvironment; challenge 4: identifying spatial niches based on lineage tracing information. N, number of cells; NSCLC, non-small cell lung cancer; CITE-seq, cellular indexing of transcriptomes and epitopes by sequencing; FFPE, formalin-fixed paraffin-embedded; AML, acute myeloid leukemia.

co-presence patterns. This analysis leads to an interpretable separation of samples from different subtypes of non-small cell lung cancer (NSCLC), revealing potential connections between cell-type co-presence and NSCLC prognosis.

Φ-Space ST provides more interpretable cell-type deconvolution

In this benchmark, we applied Φ-Space ST, RCTD,[12] cell2location,[14] and TACCO[17] to the Visium NSCLC dataset.[31] A more comprehensive benchmark involving additional methods is described in STAR Methods and Figure S1. These four methods produced discrepant estimates of the spatial distribution of cell types. For example, for a tumorous lung section P11_T3 (Figure 2A), the B cell abundance in spatial spots inferred by these methods might lead to very different biological interpretations on how B cells infiltrated the tumor region (Figure 2B). Given the important role of B cells in tumor microenvironment,[38,39] these diverging estimates may result in very different cancer prognosis and treatment.

Unlike the benchmark study using simulated data, evaluating the accuracy of cell-type deconvolution is challenging due to the lack of available ground truth. Thus we defined *meaningful markers* of a target cell type in a given tissue sample as genes in the intersection of two gene sets: the differentially expressed marker genes learned from the reference data, and the spatially differentially expressed genes in the query tissue area with high predicted abundance of the target cell type (see STAR Methods). The number of meaningful markers measures how cell-type deconvolution matched prior knowledge about cell types via marker gene identification. When predicting B cell abundance in the tissue section P11_T3, Φ-Space ST had the highest number of meaningful markers (Figure 2B).

Then, to evaluate the performance of the deconvolution methods on all cell types for tissue P11_T3, we calculated the mean number of meaningful markers averaged across all 61 cell types defined in the reference (Figure S2A). Φ-Space ST had the highest mean number of meaningful markers. Finally, across all 18 tissue samples (Figure 2C), Φ-Space ST and RCTD had significantly higher mean number of meaningful markers compared to cell2location and TACCO. In addition, Φ-Space ST only took less than 1 min to compute each tissue sample, much faster than RCTD, which took hours to compute each tissue sample.

Φ-Space ST reveals lung cancer subtype-specific cell-type co-presence

With high throughput and multicellular spots, Visium samples have been viewed as particularly suitable for investigating cell-type co-presence.[40,41] A key to biologically meaningful co-presence analysis is reliable cell-type deconvolution, a task that Φ-Space ST excels at (Figures 2A–2C). Therefore, we computed the cell-type co-presence matrices based on cell-type abundance inferred by Φ-Space ST for the Visium samples. We computed one cell-type co-presence matrix for each of the 18 Visium samples, then extracted the principal components (PCs) of the co-presence matrices (details in STAR Methods).

We observed a clear separation between lung adenocarcinoma (LUAD), lung squamous cell carcinoma (LUSC), and non-tumorous samples (Figure 2D). Moreover, the LUAD and LUSC samples appeared to be more heterogeneous compared to the non-tumorous samples, with LUAD samples and LUSC samples separately grouped together. Since LUSC tends to have worse prognosis than LUAD,[42] we investigated whether the observed

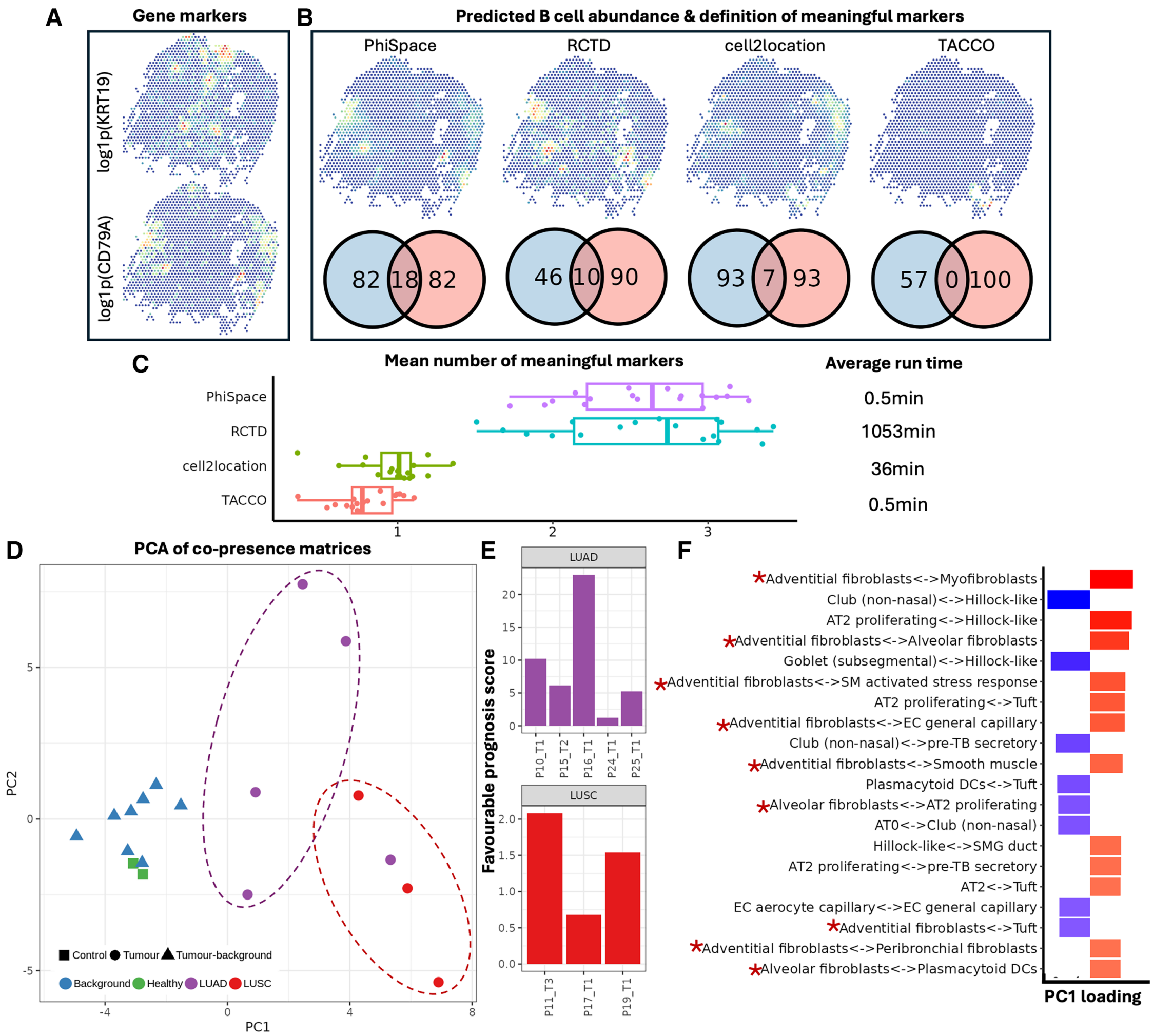

Figure 2. Benchmarking of cell-type deconvolution methods using Visium lung cancer samples

(A) A 10x Visium NSCLC tissue P11_T3 colored according to expression levels of NSCLC marker *KRT19* and B cell marker *CD79A*, where each dot represents a 55 μm multicellular spot.

(B) Predicted B cell abundance in P11_T3 according to Φ-Space ST and three state-of-the-art cell-type deconvolution methods. These four methods resulted in very different estimates of B cell spatial distribution. To evaluate the quality of B cell abundance predictions, we calculated the number of meaningful markers for B cell prediction by each method: in each Venn diagram, the blue circle represents differentially expressed genes in the spatial region predicted to have high B cell abundance; the red circle represents B cell marker genes learned from the reference.

(C) Boxplot comparing mean number of meaningful markers per tissue for all 18 tissues. Average run time of each method is also shown.

(D) We computed cell-type co-presence matrices for 18 lung tissues and computed their first two PCs with proportions of variance explained by these PCs. The PC plot shows a clear separation between non-tumorous (healthy and background) and tumorous (LUAD and LUSC) samples. Furthermore, LUSC, which tend to have worse prognosis than LUAD, tended to be separated from LUAD. (LUAD, lung adenocarcinoma; LUSC, lung squamous cell carcinoma; background, non-tumorous tissues from cancerous lungs.).

(E) To investigate if the heterogeneity of tumorous samples observed in D reflected differential prognoses, we computed the favorable prognosis scores for both LUAD and LUSC samples. P24_T1 had the least favorable predicted prognosis among the LUAD samples, and was located closer to LUSC samples in (D); P11_T3 had the most favorable predicted prognosis among the LUSC samples and was located closer to LUAD samples. In general, tumorous samples tended to form two clusters (circled in D) according to prognoses.

(F) Since different disease conditions were mainly separated along PC1 in (D), we plotted the top 20 cell-type co-presence patterns contributing to PC1: 9/20 involved fibroblasts (marked by ⋆). See also Figures S2–S5.

separation might be due to the differential prognoses of cancer patients. As we lacked additional patient-level information from the original publication,[31] we opted for a computational approach to predict prognosis based on markers identified in the Visium samples[42] (see STAR Methods). Our analysis identified LUAD (LUSC) samples with the least (most) favourable prognosis (Figure 2E). This was confirmed by the location of sample P24_T1 (least favorable prognosis) close to the LUSC samples and P11_T3 (most favorable prognosis) close to the LUAD samples (Figure 2D). Therefore, we were able to attribute the

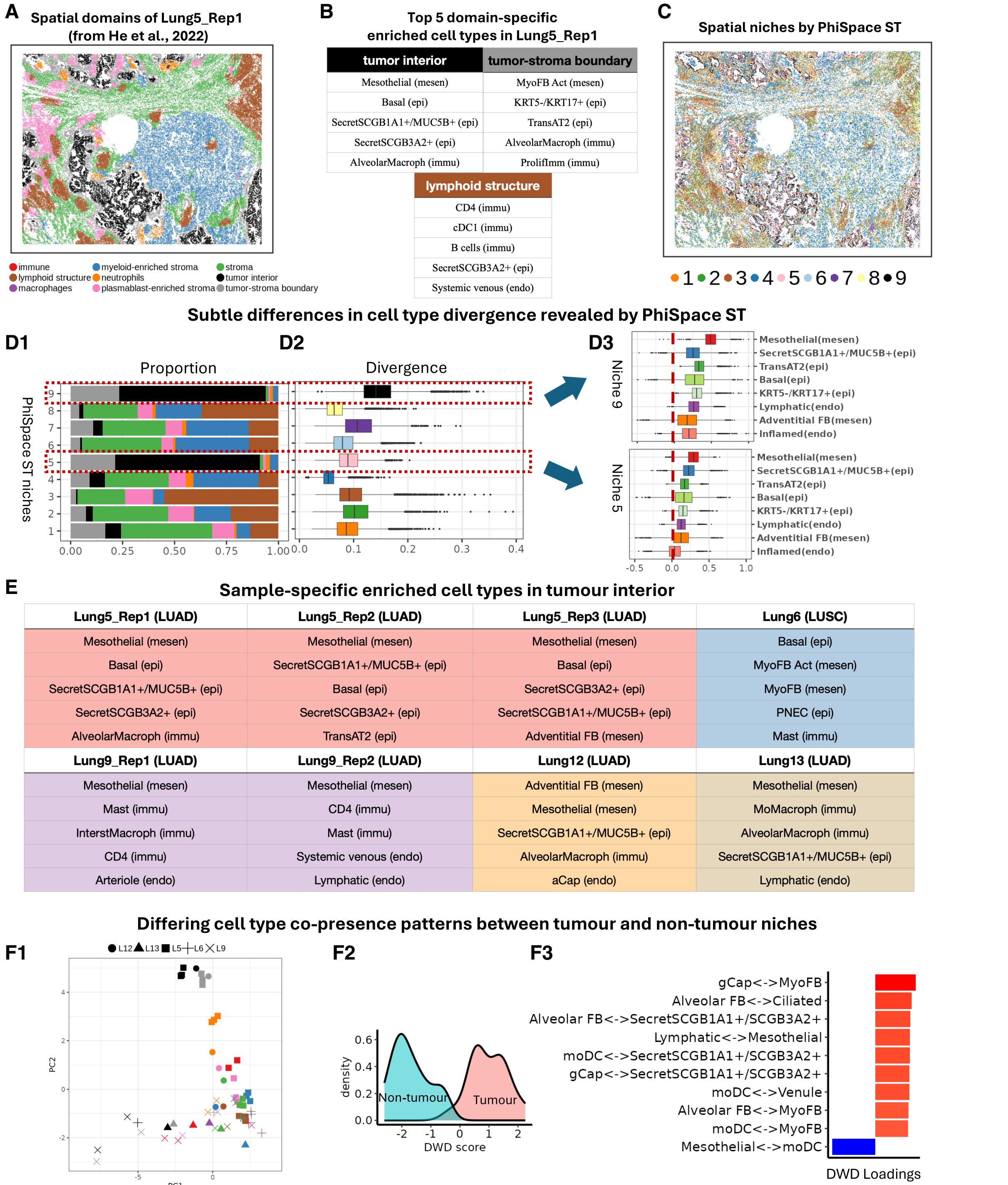

Figure 3. Exploring cell-state heterogeneity in CosMx lung samples

(A) CosMx lung sample Lung5_Rep1 from a patient with LUAD, with spatial domains identified by He et al.[33] Each dot represents the location of the nucleus of a cell.

(B) Top 5 most enriched cell types of some spatial domains in the sample Lung5_Rep1 identified by Φ-Space ST. (SecretSCGB1A1+/MUC5B+: secretory cell with unregulated SCGB1A1 and MUC5B; SecretSCGB3A2+: secretory cell with upregulated SCGB3A2; AlveolarMacroph: alveolar macrophage; MyoFB Act: activated myofibroblast; TransAT2: transitional type-2 alveolar cell; ProlifImm: proliferating immune cell; CD4: CD4 T cell; cDC1: type-1 conventional dendritic cell).

(C) Φ-Space ST niches, identified by clustering phenotype space embeddings of segmented cells.

(legend continued on next page)

heterogeneity of tumorous samples observed on Figure 2D to their differential prognoses.

Since the disease conditions were mainly separated along the first principal component (PC1) on Figure 2D, we investigated how co-presence of specific pairs of cell types contributed to this separation by examining the top 20 PC1 cell-type co-presence loadings (Figure 2F). Notably, adventitial and alveolar fibroblasts were featured in 9 of the top 20 loadings, highlighting the diverse roles of fibroblasts in lung tumor microenvironment.[43,44] To focus solely on immune cell types, we excluded non-immune cell types in the co-presence matrices and reran the analysis. We observed that the separation between LUAD, LUSC, and non-tumorous tissues was largely retained (Figure S2B), with plasmacytoid dendritic cell (pDC) playing a prominent role in explaining this separation (Figure S2C).[45] In particular, the co-presence of pDC and different types of fibroblasts all had positive loadings, suggesting that the pDC-fibroblast co-presence might be related to lung cancer prognosis.[46]

To further examine this association between pDC and fibroblasts, we extended the analysis to all 40 Visium samples (including samples not used in the co-presence PCA) and computed pDC-fibroblast Spearman correlations for each sample (see Figure S4 for some representative samples). All five fibroblast subtypes showed significantly higher pDC co-presence in tumor compared to non-tumor tissue, with a clear gradient from healthy to background to LUAD to LUSC (Figure S5). The tumor-adjacent background samples showed pDC-fibroblast co-presence levels comparable to healthy tissue, indicating that the elevated co-presence is specific to the tumor microenvironment.

Deconvolution results from other methods can also be used to conduct the cell-type co-presence analysis featured in Figures 2D–2F. However, results based on alternative methods were less interpretable compared to Φ-Space ST. For example, although the first two PCs of co-presence matrices based on RCTD also showed a good separation of disease conditions, the PCs there lacked a correspondence to cancer prognosis as in the Φ-Space ST results above (Figure S3).

Φ-Space ST characterizes heterogeneity of lung tumor microenvironment using healthy single-cell reference atlas

Φ-Space ST can combine multiple scRNA-seq references to uncover complex cell states in tumor microenvironment, as we demonstrate using the CosMx dataset from NSCLC patients. Since cell segmentation was available for these tissue sections,[33] we treated segmented cells as cell-like objects.

Φ-Space ST uncovers complex and heterogeneous cell-state landscapes of tumor microenvironment

We first focused on a particular lung section Lung5_Rep1 from a LUAD patient to demonstrate how Φ-Space ST can provide insights into the cell-state heterogeneity of the tumor microenvironment. Nine spatial domains were identified in this tissue section by He et al.[33] (Figure 3A). According to our Φ-Space ST annotation, the tumor interior, tumor-stroma boundary and lymphoid structure domains had higher cell-type divergence (Figure S6; see also STAR Methods). The lymphoid structures were highly divergent, potentially due to the dense population of various immune cell types, such as T, B, and dendritic cells[47] as identified by Φ-Space ST (Figure 3B). The tumor domains were highly divergent, consistent with lung tumors tending to exhibit molecular features of multiple cell types.[42] We found that a combination of mesenchymal, epithelial, and immune cell types were enriched in the tumor domains, including tumor interior and tumor-stroma boundary (Figure 3B). In particular, the secretory and transitional AT2 cell types reflected the glandular cell origin of LUAD[48]; the observed basal and *KRT5-/KRT17+* epithelial cell types in LUAD was consistent with previous study[49]; and the presence of mesothelial and activated myofibroblast cell types flagged the potential existence of epithelial-to-mesenchymal transition[50] and cancer-associated fibroblasts.[51]

Φ-Space ST identifies heterogeneous tumor niches with differential cell-type divergence

To compare Φ-Space ST spatial niches with the spatial domains identified by He et al.[33] (Figure 3A), we identified nine spatial niches of Lung5_Rep1 based on the Φ-Space ST annotation (Figure 3C; see STAR Methods). Overall, our Φ-Space ST niches corresponded well to the domains in Figure 3A. A notable difference was that we identified two tumor niches, niches 5 and 9, which both contained a large proportion of cells from the tumor interior and tumor-stroma boundary domains (Figure 3D1), but had significantly different cell-state divergence (Figure 3D2). In particular, these two niches were enriched by the same cell types but niche 9 showed a much higher divergence compared to niche 5 (Figures 3D2 and D3). This highlighted intra-tumor heterogeneity that was not captured in He et al.[33]

Φ-Space ST identifies differentially enriched cell types in different cancerous lung samples

As we have previously demonstrated,[20] the phenotype space embeddings of query datasets derived using PLS regression models are robust against batch effects. This property of PLS also makes Φ-Space ST annotations from different query ST samples directly comparable. For example, the enriched cell

(D) Cell-type divergence of Φ-Space ST niches. (D1) Cell proportions according to spatial domains (A) for each Φ-Space ST niche. Niches 5 and 9 contained large proportions of cells from tumor domains (tumor interior and tumor-stroma boundary). (D2) Cell-type divergence of Φ-Space ST niches. The two tumor niches 5 and 9 differ greatly in their divergence. (D3) Φ-Space ST scores of the most enriched cell types in niches 5 and 9. While these two niches had similar enriched cell types, the scores in niche 9 were generally higher than in niche 5, leading to the difference in cell-type divergence.

(E) Top 5 most enriched cell types in tumor interior domains of different lung samples. (LUAD: lung adenocarcinoma; LUSC: lung squamous cell carcinoma).

(F) Cell-type co-presence analysis. (F1) First two PCs of all the domain-specific co-presence matrices. Color represents spatial domains as in (A); shapes represent sample ID. Tumor domains (tumor interior and tumor-stroma boundary) and non-tumor domains were separated, reflecting distinct cell-type co-presence patterns. (F2) Discriminant analysis (DWD) of all domain-specific co-presence matrices shows clear separation between tumor and non-tumor domains. (F3) DWD loadings highlighting most discriminant co-presence patterns, where two cell types having positive (negative) loading indicates if their co-presence contributes to the prediction of tumor (non-tumor) domains. (gCap: general capillary cell; FB: fibroblast; Secret: secretory cell; moDC: monocyte-derived dendritic cell.) See also Figure S6 and Table S1.

types in the tumor interior domain were reproducible across biological replicates, i.e., tissue sections from the same lungs (see replicates from Lung5 and Lung9 in Figure 3E). In contrast, the enriched cell types in the tumor interior domain from different patients exhibited significant heterogeneity (compare Lung5, Lung6, Lung9, Lung12, and Lung13 in Figure 3E). Specifically, mesothelial cell type was enriched in all LUAD tumors (Lung5, Lung9, Lung12, and Lung13) but not in LUSC sample (Lung6). Two myofibroblast cell types were enriched in the LUSC sample (Lung6), consistent with a recent finding that LUSC tumors tended to have higher level of myofibroblasts compared to LUAD.[52]

Φ-Space ST reveals cancer-specific cell-type co-presence patterns

To investigate how cell-type co-presence patterns distinguish spatial niches across samples, we calculated the cell-type co-presence matrices for spatial domains in all eight CosMx NSCLC samples, visualized using PCA (detailed in STAR Methods). We observed a separation of tumor domains from non-tumor domains (Figure 3F1). Furthermore, a discriminant analysis using distance-weighted discrimination (DWD) (Figure 1C) led to an improved separation between tumor and non-tumor domains (Figure 3F2). The DWD loadings highlighted pairs of cell types whose co-presence in the same segmented cell contribute to the prediction of tumor or non-tumor domains (Figure 3F3). The tumor domains were characterized by complex interactions between endothelial (gCap, lymphatic, and venule), epithelial (ciliated and secretory), and mesenchymal (myoFB, alveolar FB, and mesothelial) cell types, indicating possible epithelial-mesenchymal, mesenchymal-epithelial, and endothelial-mesenchymal transitions as typically seen in malignant tumors.[25,53] In addition, the role of monocyte-derived dendritic cells (moDC) was also highlighted. The co-presence of moDC, secretory, venule, and myofibroblast cell types in tumor domains was consistent with a previous observation that myeloid DCs (including moDC) were commonly observed in NSCLC specimens[54]; however, the co-presence of moDC and mesothelial tended to be observed in non-tumor domains, which may reflect the shared role of mesothelial cells and moDCs in antigen presentation.[55]

Φ-Space ST enables cross-platform validation of spatial cell-type co-presence patterns

To further validate the platform-agnostic nature of Φ-Space ST, we applied it to a 10x Genomics Xenium *in situ* dataset of formalin-fixed paraffin-embedded (FFPE) human NSCLC tissue.[56] The Xenium platform detects transcripts at subcellular resolution using targeted probe panels. This dataset contains 161,894 segmented cells profiled across 377 genes, with 89% of cells identified as tumor and 11% as stroma or unannotated by a pathologist (Figure 4A).

We used the same four lineage-specific scRNA-seq references from Natri et al.[32] as in the CosMx analysis, covering immune, epithelial, endothelial, and mesenchymal lineages. Although substantially smaller than the 980-gene CosMx panel, the Xenium 377-gene panel was curated to include cancer-related cell-type markers across tissue types. Gene overlap with the references was high (87%–97% across the four references).

Spatial niche clustering (see STAR Methods) identified distinct tumor and stromal niches (Figure 4B; see Figure S7 for the selection of the number of niches). The highest tumor niches (4, 8, and 9 with >80% tumor; Figure 4C) were enriched for secretory or AT2 epithelial cells, consistent with the glandular cell origin of LUAD also observed in the CosMx analysis. Low-tumor niches (6 and 7) were enriched for stromal cells (e.g., AT1, ciliated), immune, and fibroblast subtypes.

PCA of niche-specific co-presence matrices revealed cell-type pairs driving the greatest inter-niche variation in co-presence (Figure S8A). Secretory-involved and fibroblast-involved pairs feature prominently among the top loadings (Figures S8B and S8C), recapitulating our CosMx finding (Figure 3F3). In particular, fibroblast-involved co-presence patterns as drivers of niche-level variation are consistent across all three lung cancer case studies: Visium (Figure 2F), CosMx (Figure 3F), and Xenium (Figures S8B and S8C).

Φ-Space ST achieves segmentation-free annotation of subcellular ST data

Clonally resolved ST data links spatial information with clonal identity from lineage tracing. This technological advance promises to uncover transcriptional mechanisms that contribute to intratumor heterogeneity of cancer clones.[57,58] However, clonally resolved ST data remain rare and under-explored, with no established workflow. To address this challenge, we developed a Φ-Space ST-based workflow to provide unique spatial insights by incorporating information from cancer lineage tracing (Figure 5A). We investigated the heterogeneity of acute myeloid leukemia (AML) clones based on a Stereo-seq mouse spleen dataset[58] (details in Table 1). Liquid tumors such as AML tumors have less clearly defined spatial structures compared to solid tumors,[59] which adds to the challenges in analyzing this dataset.

This Stereo-seq dataset lacks reliable cell segmentation, so we binned the whole tissue slide into bins of sidelengths ∼25 μm (as in Holze et al.[58]). Therefore, cell-like objects in this case study were spatial bins. Since a comprehensive mouse spleen cell atlas is not available, we used Φ-Space ST to leverage multiple scRNA-seq reference datasets without the need for prior integration (details in STAR Methods). Since we wished to make use of a scRNA-seq dataset generated from the same mouse spleen as the Stereo-seq sample,[57] we used a "bridging" strategy for continuous annotation transfer with Φ-Space ST. Our approach is very different from other deconvolution methods which cannot transfer continuous annotations: we first annotated the intermediate scRNA-seq spleen sample, then used the scRNA-seq spleen sample (with continuous Φ-Space ST annotations) to annotate the Stereo-seq sample.

Φ-Space ST multi-reference annotation provides a comprehensive characterization of AML spleen

The mouse spleen has a distinctive morphology, where T and B lymphocytes in the white pulp coordinate adaptive immune responses. Surrounding the white pulp is the marginal zone, a region enriched with macrophages, dendritic cells, and marginal zone B cells that capture antigens and facilitate their delivery to lymphocytes in the white pulp[60] (Figure 5B1). Specific to this spleen sample was the prevalence of *Elane* expression (a

CellPress
OPEN ACCESS

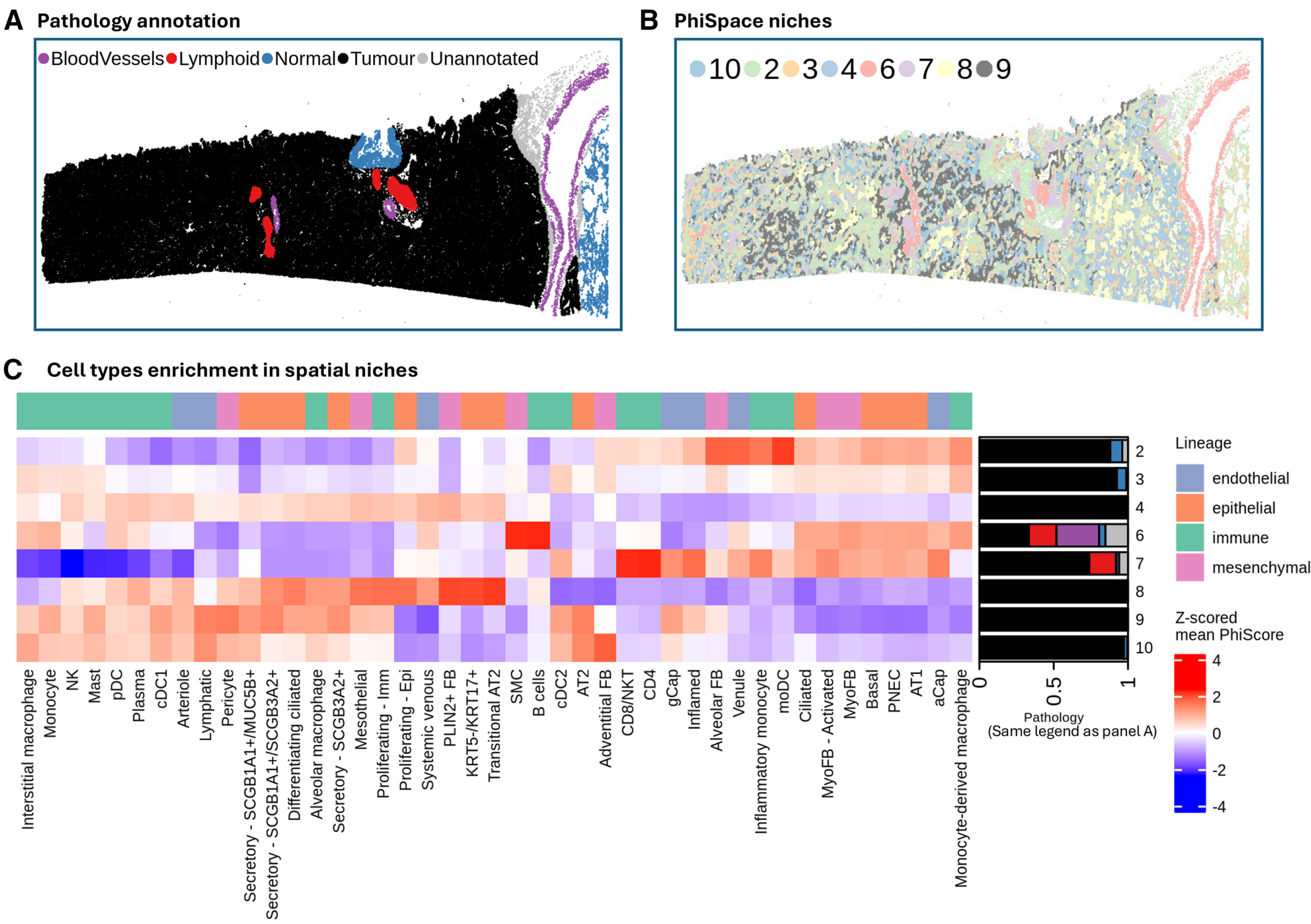

Figure 4. Validation of tumor hallmarks in Xenium lung samples

(A) Annotation of Xenium lung sample by a pathologist.

(B) Niches identified by clustering spatially smoothed Φ-Space ST scores. We selected 8 niches after comparing clustering results with the number of niches varying from 2 to 10 (Figure S7).

(C) Cell-type enrichment in different niches, shown as a heatmap with *Z*-scored mean Φ-Space ST scores. The top bar indicates the lineage of a given cell type (a column in the heatmap). The right image shows the composition of pathological annotations in each niche, with most niches having dominant proportions of tumor except 6 and 7. See also Figures S7 and S8.

neutrophil marker). As lineage tracing information was available, we were able to locate the AML cells (Figure 5B2).

The four reference datasets we used contained immune cell types from different mouse tissue types, including mouse spleen, bone marrow (origin of the myeloid lineages of immune cells), and tumor (details in Table 1). These references jointly provide a comprehensive characterization of the immune landscape in AML mouse spleen. We first clustered the spatial bins based on their multi-reference phenotype space embeddings, which resulted in more subtle spatial niches compared to the clusters based on gene expression (Figure 5B3; see STAR Methods). Thus, our Φ-Space ST approach successfully captured biologically meaningful spatial variations across morphological structures in the spleen.

We further evaluated whether the Φ-Space ST cell-type scores could reveal the differences between bins that contained AML barcodes and non-barcoded bins. Our DWD analysis (detailed in STAR Methods) showed that barcoded bins tended to have stronger ICOS+ regulatory T cell (ICOS+ Treg), red blood cell (RBC), neutrophil (Neutro, T1), hematopoietic progenitor cell (HPC), and CD4 T cell identities, compared to non-barcoded bins (Figure 5C). The strong ICOS+ Treg identity of barcoded bins likely reflects the ability of AML cells to promote the expansion of Tregs by expressing ICOS ligand.[61] The neutrophil identity of the barcoded region may be explained by the high expression of the neutrophil marker *Elane* in that region (Figure 5B1). Notably, the barcoded and non-barcoded bins were enriched by different types of neutrophils, with barcoded bins enriched by tumor-associated T1 neutrophils and non-barcoded bins enriched by mature neutrophils (MAT2 and MAT3).

AML clones form phenotypically distinct meta-clones

In this clonally resolved Stereo-seq dataset, we observed that individual AML clones in mouse spleen tended to be concentrated in specific spatial domains, which motivated us to define "meta-clones," i.e., spatial niches of bins with similar composition of AML clones (Figure S9). We aggregated the spatial bins into meta-clones according to their clonal compositions and identified eight meta-clones with significantly different compositions of individual AML clones (Figures 5D1 and 5D2; details in STAR Methods).

The AML meta-clones exhibited differential cell states, characterized by their most enriched cell types compared to non-barcoded bins (Figures5D3; details in STAR Methods). We observed that meta-clone 7 formed the "periphery" of meta-clone 2. Both

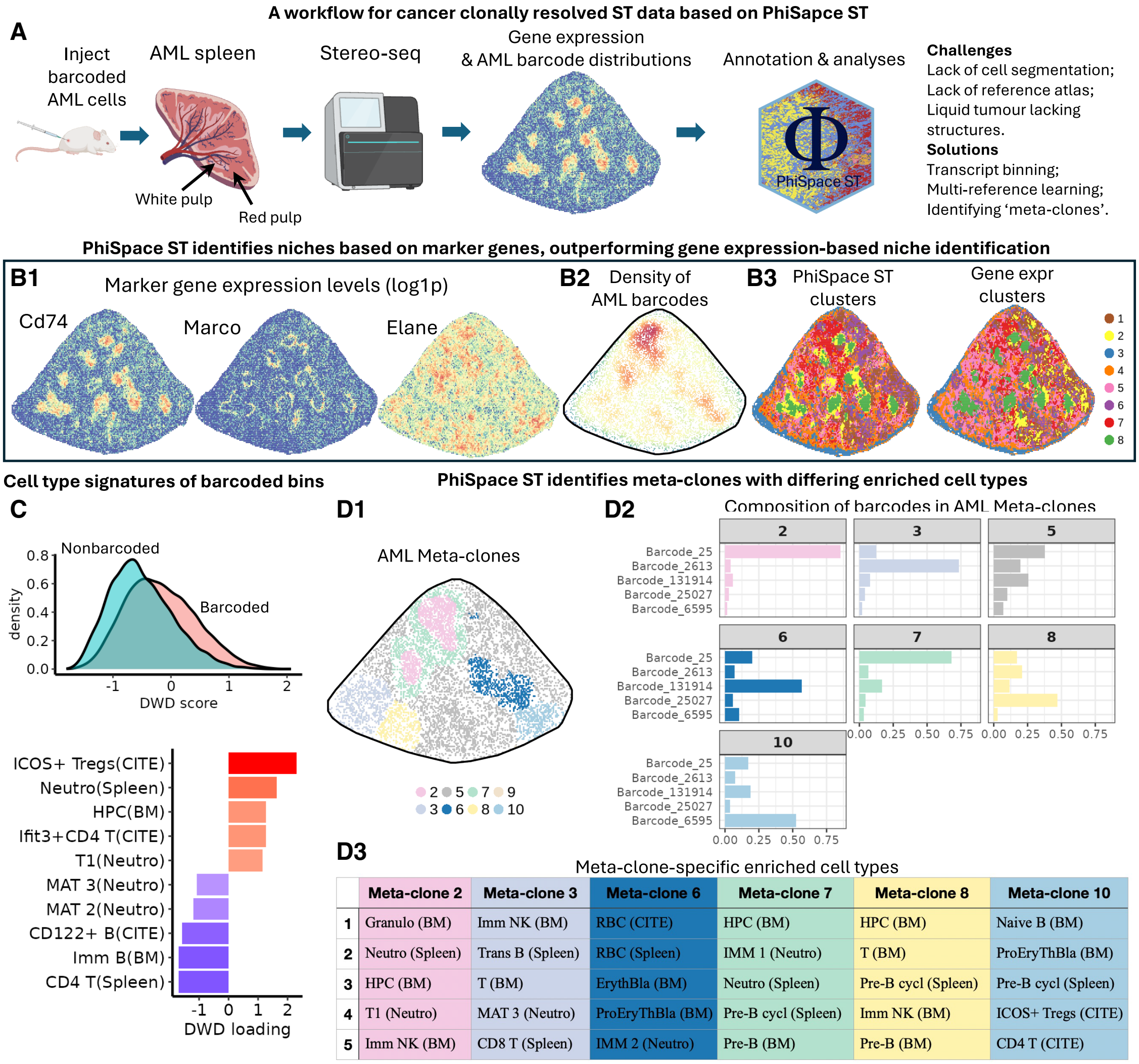

	Meta-clone 2	Meta-clone 3	Meta-clone 6	Meta-clone 7	Meta-clone 8	Meta-clone 10
1	Granulo (BM)	Imm NK (BM)	RBC (CITE)	HPC (BM)	HPC (BM)	Naive B (BM)
2	Neutro (Spleen)	Trans B (Spleen)	RBC (Spleen)	IMM 1 (Neutro)	T (BM)	ProEryThBla (BM)
3	HPC (BM)	T (BM)	ErythBla (BM)	Neutro (Spleen)	Pre-B cycl (Spleen)	Pre-B cycl (Spleen)
4	T1 (Neutro)	MAT 3 (Neutro)	ProEryThBla (BM)	Pre-B cycl (Spleen)	Imm NK (BM)	ICOS+ Tregs (CITE)
5	Imm NK (BM)	CD8 T (Spleen)	IMM 2 (Neutro)	Pre-B (BM)	Pre-B (BM)	CD4 T (CITE)

Figure 5. Uncovering cancer clone-specific cell states in Stereo-seq AML mouse spleen data

(A) Overview of the workflow for analyzing clonally resolved ST data based on Φ-Space ST. Stereo-seq AML mouse spleen data with lineage tracing information were obtained from an AML mouse with barcoded cancer cells. (Created in https://BioRender.com.) Using gene expression and barcode information as input, Φ-Space ST identifies AML "meta-clones" with differential enriched cell types based on multiple reference datasets.

(B) Φ-Space ST identifies more nuanced spatial niches compared to conventional gene expression-based niche identification. (B1) Spatial distribution of morphological gene markers *Cd74* (white pulp), *Marco* (marginal zone), and *Elane* (neutrophil). Each dot represents a spatial bin of sidelengths ∼25 μm where at least one transcript was detected. (B2) Density of all AML barcodes in the mouse spleen sample. (B3) Eight clusters of spatial bins were identified based on either Φ-Space ST phenotype space embeddings or gene expressions of the bins. The Φ-Space ST clusters better delineated marginal zone and the high barcode density regions shown in A2 compared to the gene expression clusters.

(C) A DWD discriminant analysis revealed differences between bins that contained AML barcodes ("barcoded") and bins that did not contain any barcodes ("non-barcoded"). Barcoded bins tended to have larger DWD scores. Top 10 DWD loadings (ranked by absolute value) show most important cell types for distinguishing barcoded and non-barcoded bins. (CITE, Spleen, BM, Neutro: four reference datasets; Treg, regulatory T cell; Neutro, neutrophil; HPC, hematopoietic progenitor cell; T1, tumor-associated neutrophil; Mat 2, 3, mature neutrophil; Imm B, immature B cell).

(D) AML meta-clones and their enriched cell types. (D1) Eight meta-clones were identified based on their barcode compositions. Bins with similar barcode (clonal) compositions were grouped into the same meta-clone. (D2) Proportion of 5 most abundant barcodes in each meta-clone (with the exception of meta-clone 9 that is too rare). The meta-clones had a diverse composition of individual AML clones, where all meta-clones, except 5, had a dominating AML clone. (D3) Enriched cell types within individual meta-clones. Compared to (F), neighboring meta-clones (i.e., 2&7, 6&10, and 3&8) tended to have similar enriched cell types, whereas non-neighboring meta-clones tended to have dissimilar enriched cell types (Granulo, granulocyte, including neutrophil; IMM NK, immature natural killer; Trans B, transitional B cell; RBC, red blood cell; (Pro)ErythBla, (pro)erythroblast; Imm 1, 2, immature neutrophil; Pre-B (cycl), (cycling) pre-B cell). See also Figures S9–S13 and Table S2.

CellPress
OPEN ACCESS

meta-clones had strong neutrophil identities. The neighboring meta-clones 6 and 10 both had strong erythroid identities while the neighboring meta-clones 3 and 8 both had strong lymphoid identities (Figures 5D2 and 5D3). In addition, non-neighboring meta-clones tended to have enriched cell types from different lineages. These observations provided unique insights into the spatial organization of AML tumors and their heterogeneity in terms of cell states, which remain poorly understood.

To provide a more comprehensive characterization of meta-clone-specific cell states, we computed the mean Φ-Space ST score for all 70 cell types within each meta-clone (Figure S11), confirming that neighboring meta-clones shared similar enriched cell types. To extend the analysis beyond cell-type identities, we scored each spatial bin for 11 biologically relevant gene expression signatures from the MSigDB Hallmark collection[62] using UCell.[63] Meta-clone 2 was strongly depleted for proliferation signatures (G2M checkpoint, E2F targets) and TNF-α/NF-κB signaling, but enriched for complement and oxidative phosphorylation. Meta-clone 6 was enriched for oxidative phosphorylation and MYC targets, suggesting a proliferation-associated state directly contrasting meta-clone 2 (Figure S13A). Correlation analysis further linked cell-type identity to gene expression signatures, with progenitor-like cell types associated with proliferative signatures and macrophages associated with complement and interferon-γ response (Figure S13B).

DISCUSSION

Φ-Space ST addresses key challenges in annotating cell states in complex tissues and disease environments. By leveraging the power of PLS regression to annotate cell-like objects on a continuous scale using multiple references, Φ-Space ST provides a unified, platform-agnostic, and computationally efficient framework for analyzing ST data at multiple resolutions. The continuous cell-state annotation enables the characterization of both known and previously uncharacterized cell types, overcoming limitations of traditional methods that rely on single reference and are computationally costly.

In addition to enabling the unique downstream analyses demonstrated in our case studies, we demonstrated that Φ-Space ST, when its continuous scores are converted to proportions, achieves competitive accuracy with established deconvolution methods. Moreover, the pDC-fibroblast co-presence pattern identified in the Visium case study points to a promising research direction. While recent large-scale spatial proteomics studies of NSCLC have identified DCs and fibroblasts as key tumor microenvironment components,[64] further validation in clinically annotated spatial cohorts with DC subtype resolution would be valuable to establish a definitive link between pDC-fibroblast co-presence and patient prognosis.

Future work will focus on extending Φ-Space ST to other pathological conditions and integrating additional omics assays, such as spatial proteomics and epigenomics, to further expand its applicability.

Limitations of the study

This study has several limitations. First, Φ-Space ST relies on the availability and quality of scRNA-seq reference datasets; cell states absent from all references cannot be directly identified, though they may manifest as low scores across all cell types. Second, for subcellular ST platforms without reliable cell segmentation, transcript binning introduces an arbitrary spatial resolution that may not correspond to true cell boundaries. Third, while we demonstrated cross-platform consistency of key findings across Visium, CosMx, and Xenium, all three lung cancer case studies analyzed NSCLC tissue; the generalizability of our biological findings to other cancer types and organs remains to be validated. Finally, the deconvolution benchmark used simulated data with known ground truth, which may not fully capture the complexity of real tissue samples.

RESOURCE AVAILABILITY

Lead contact

Further information and requests for resources should be directed to and will be fulfilled by the lead contact, Kim-Anh Lê Cao (kimanh.lecao@unimelb.edu.au).

Materials availability

This study did not generate new unique reagents or materials.

Data and code availability

The Φ-Space R package and analysis code are available on GitHub (https://github.com/jiadongm/PhiSpace) and archived on Zenodo (https://doi.org/10.5281/zenodo.19941499). This study utilized publicly available datasets which are listed in the key resources table. Any additional information required to reanalyze the data reported in this paper is available from the lead contact upon request.

ACKNOWLEDGMENTS

We would like to thank Prof. Mark Dawson, Dr. Dane Vassiliadis, and Ms. Henrietta Holze (Sir Peter MacCullum Cancer Center) for providing the processed AML mouse spleen Stereo-seq data and their helpful suggestions for writing the Stereo-seq case study. We would also like to thank Mr. Yidi Deng (University of Melbourne) for helpful discussions. J.M. and K.A.L.C. were supported in part by the National Health and Medical Research Council Investigator Grant (GNT2025648).

AUTHOR CONTRIBUTIONS

J.M. developed the Φ-Space ST method, performed all computational analyses, and drafted the manuscript; J.C. contributed to conception of the project, biological interpretation, and manuscript revision; K.A.L.C. conceived and supervised the project and contributed to manuscript writing; J.C. and K.A.L.C. contributed equally to this work.

DECLARATION OF INTERESTS

The authors declare they have no competing interests.

DECLARATION OF GENERATIVE AI AND AI-ASSISTED TECHNOLOGIES IN THE WRITING PROCESS

During the writing process of this work, the authors used Claude (Anthropic, model Opus 4.6) in order to assist with polishing manuscript texts, correcting typos, and grammatical mistakes. After using this tool, the authors reviewed and edited the content as needed and take full responsibility for the content of the publication.

STAR★METHODS

Detailed methods are provided in the online version of this paper and include the following:

- KEY RESOURCES TABLE
- EXPERIMENTAL MODEL AND STUDY PARTICIPANT DETAILS
  - Human tissue datasets
  - Mouse datasets
- METHOD DETAILS
  - Multi-reference continuous phenotyping
  - Spatial niche identification
  - Data preprocessing
  - Parameter settings for case studies
  - Deconvolution benchmark setup
  - Stereo-seq spatial bin clustering
  - Meta-clone identification
  - Gene expression signature scoring
  - Cross-reference consistency analysis
- QUANTIFICATION AND STATISTICAL ANALYSIS
  - Niche-specific enriched cell types
  - Cell-type co-presence
  - Enriched cell types in AML meta-clones
  - Meta-clone-specific gene expression signatures

SUPPLEMENTAL INFORMATION

Supplemental information can be found online at https://doi.org/10.1016/j.crmeth.2026.101483.

Received: October 14, 2025
Revised: March 3, 2026
Accepted: May 12, 2026
Published: June 12, 2026

REFERENCES

1. Cheng, M., Jiang, Y., Xu, J., Mentis, A.F.A., Wang, S., Zheng, H., Sahu, S.K., Liu, L., and Xu, X. (2023). Spatially resolved transcriptomics: a comprehensive review of their technological advances, applications, and challenges. J. Genet. Genomics *50*, 625–640. https://doi.org/10.1016/j.jgg.2023.03.011.

2. 10x Genomics (2024a). Visium spatial gene expression: Map the whole transcriptome within the tissue context. https://www.10xgenomics.com/products/spatial-gene-expression.a. .

3. 10x Genomics (2026). Xenium in situ platform. https://www.10xgenomics.com/platforms/xenium. . Accessed: 13 Feb 2026.

4. NanoString Technologies (2024). CosMx smi for single-cell imaging. https://nanostring.com/products/cosmx-spatial-molecular-imager/single-cell-imaging-overview/. . Accessed: 20 Sep 2024.

5. BGI Genomics (2024). Spatial transcriptome Stereo-seq. https://www.bgi.com/global/service/spatial-transcriptome-stereo-seq.

6. Dann, E., Cujba, A.M., Oliver, A.J., Meyer, K.B., Teichmann, S.A., and Marioni, J.C. (2023). Precise identification of cell states altered in disease using healthy single-cell references. Nat. Genet. *55*, 1998–2008. https://doi.org/10.1038/s41588-023-01523-7.

7. Lotfollahi, M., Yuhan, H., Theis, F.J., and Satija, R. (2024). The future of rapid and automated single-cell data analysis using reference mapping. Cell *187*, 2343–2358. https://doi.org/10.1016/j.cell.2024.03.009.

8. Tian, L., Chen, F., and Macosko, E.Z. (2023). The expanding vistas of spatial transcriptomics. Nat. Biotechnol. *41*, 773–782. https://doi.org/10.1038/s41587-022-01448-2.

9. Petukhov, V., Xu, R.J., Soldatov, R.A., Cadinu, P., Khodosevich, K., Moffitt, J.R., and Kharchenko, P.V. (2022). Cell segmentation in imaging-based spatial transcriptomics. Nat. Biotechnol. *40*, 345–354. https://doi.org/10.1038/s41587-021-01044-w.

10. Ma, Y., and Zhou, X. (2024). Accurate and efficient integrative reference-informed spatial domain detection for spatial transcriptomics. Nat. Methods *21*, 1231–1244. https://doi.org/10.1038/s41592-024-02284-9.

11. Zhang, H., Hunter, M.V., Chou, J., Quinn, J.F., Zhou, M., White, R.M., and Tansey, W. (2023a). BayesTME: An end-to-end method for multiscale spatial transcriptional profiling of the tissue microenvironment. Cell Syst. *14*, 605–619.e7. https://doi.org/10.1016/j.cels.2023.06.003.

12. Cable, D.M., Murray, E., Zou, L.S., Goeva, A., Macosko, E.Z., Chen, F., and Irizarry, R.A. (2022). Robust decomposition of cell type mixtures in spatial transcriptomics. Nat. Biotechnol. *40*, 517–526. https://doi.org/10.1038/s41587-021-00830-w.

13. Chen, J., Liu, W., Luo, T., Yu, Z., Jiang, M., Wen, J., Gupta, G.P., Giusti, P., Zhu, H., Yang, Y., and Li, Y. (2022). A comprehensive comparison on cell-type composition inference for spatial transcriptomics data. Brief. Bioinform. *23*, bbac245. https://doi.org/10.1093/bib/bbac245.

14. Kleshchevnikov, V., Shmatko, A., Dann, E., Aivazidis, A., King, H.W., Li, T., Elmentaite, R., Lomakin, A., Kedlian, V., Gayoso, A., et al. (2022). Cell2location maps fine-grained cell types in spatial transcriptomics. Nat. Biotechnol. *40*, 661–671. https://doi.org/10.1038/s41587-021-01139-4.

15. Li, B., Zhang, W., Guo, C., Xu, H., Li, L., Fang, M., Hu, Y., Zhang, X., Yao, X., Tang, M., et al. (2022a). Benchmarking spatial and single-cell transcriptomics integration methods for transcript distribution prediction and cell type deconvolution. Nat. Methods *19*, 662–670. https://doi.org/10.1038/s41592-022-01480-9.

16. Li, H., Zhou, J., Li, Z., Chen, S., Liao, X., Zhang, B., Zhang, R., Wang, Y., Sun, S., and Gao, X. (2023). A comprehensive benchmarking with practical guidelines for cellular deconvolution of spatial transcriptomics. Nat. Commun. *14*, 1548. https://doi.org/10.1038/s41467-023-37168-7.

17. Mages, S., Moriel, N., Avraham-Davidi, I., Murray, E., Watter, J., Chen, F., Rozenblatt-Rosen, O., Klughammer, J., Regev, A., and Nitzan, M. (2023). TACCO unifies annotation transfer and decomposition of cell identities for single-cell and spatial omics. Nat. Biotechnol. *41*, 1465–1473. https://doi.org/10.1038/s41587-023-01657-3.

18. Deng, Y., Bartosovic, M., Ma, S., Zhang, D., Kukanja, P., Xiao, Y., Su, G., Liu, Y., Qin, X., Rosoklija, G.B., et al. (2022). Spatial profiling of chromatin accessibility in mouse and human tissues. Nature *609*, 375–383. https://doi.org/10.1038/s41586-022-05094-1.

19. Liu, Y., DiStasio, M., Su, G., Asashima, H., Enninful, A., Qin, X., Deng, Y., Nam, J., Gao, F., Bordignon, P., et al. (2023). High-plex protein and whole transcriptome co-mapping at cellular resolution with spatial CITE-seq. Nat. Biotechnol. *41*, 1405–1409. https://doi.org/10.1038/s41587-023-01676-0.

20. Mao, J., Deng, Y., and Lê Cao, K.A. (2025). Φ-space: continuous phenotyping of single-cell multi-omics data. Genome Biol. *26*, 1–25. https://doi.org/10.1186/s13059-025-03504-5.

21. Shu, H., Chen, J., Xu, C., Hu, J., Wang, Y., Peng, J., Jiang, Q., Shang, X., and Wang, T. (2025). Efficient integration of spatial omics data for joint domain detection, matching, and alignment with stMSA. Genome Res. *35*, 2285–2299. https://doi.org/10.1101/gr.280584.125.

22. Zhen, P., Wang, X., Shu, H., Hu, J., Wang, Y., Peng, J., Shang, X., Chen, J., and Wang, T. (2025). DisConST: Distribution-aware contrastive learning for spatial domain identification. Genom. Proteom. Bioinform. qzaf085. https://doi.org/10.1093/gpbjnl/qzaf085.

23. Zhu, P., Shu, H., Wang, Y., Wang, X., Zhao, Y., Hu, J., Peng, J., Shang, X., Tian, Z., Chen, J., and Wang, T. (2025). MAEST: accurately spatial domain detection in spatial transcriptomics with graph masked autoencoder. Brief. Bioinform. *26*, bbaf086. https://doi.org/10.1093/bib/bbaf086.

24. Yang, C., Liu, Y., Wang, X., Jia, Q., Fan, Y., Lu, Z., Shi, J., Liu, Z., Chen, G., Li, J., et al. (2025). stSNV: a comprehensive resource of SNVs in spatial transcriptome. Nucleic Acids Res. *53*, D1224–D1234. https://doi.org/10.1093/nar/gkae945.

CellPress
OPEN ACCESS

25. Xiao, D., and He, J. (2010). Epithelial mesenchymal transition and lung cancer. J. Thorac. Dis. *2*, 154–159. https://doi.org/10.3978/j.issn.2072-1439.2010.02.03.7.

26. Wold, H. (1966). Estimation of principal components and related models by iterative least squares. In Multivariate Analysis (Academic Press), pp. 391–420.

27. Lê Cao, K.A., and Welham, Z.M. (2021). Multivariate Data Integration Using R, 1st edition (Chapman and Hall/CRC).

28. Liang, C., FANTOM Consortium; Forrest, A.R.R., and Wagner, G.P. (2015). The statistical geometry of transcriptome divergence in cell-type evolution and cancer. Nat. Commun. *6*, 6066. https://doi.org/10.1038/ncomms7066.

29. Mao, J. Φ-Space: Continuous phenotyping for single-cell and spatial multiomics data. (2025). https://github.com/jiadongm/PhiSpacerpackageversion1.1.0.

30. Sikkema, L., Ramírez-Suástegui, C., Strobl, D.C., Gillett, T.E., Zappia, L., Madissoon, E., Markov, N.S., Zaragosi, L.E., Ji, Y., Ansari, M., et al. (2023). An integrated cell atlas of the lung in health and disease. Nat. Med. *29*, 1563–1577. https://doi.org/10.1038/s41591-023-02327-2.

31. De Zuani, M., Xue, H., Park, J.S., Dentro, S.C., Seferbekova, Z., Tessier, J., Curras-Alonso, S., Hadjipanayis, A., Athanasiadis, E.I., Gerstung, M., et al. (2024). Single-cell and spatial transcriptomics analysis of non-small cell lung cancer. Nat. Commun. *15*, 4388. https://doi.org/10.1038/s41467-024-48700-8.

32. Natri, H.M., Del Azodi, C.B., Peter, L., Taylor, C.J., Chugh, S., Kendle, R., Chung, M.I., Flaherty, D.K., Matlock, B.K., Calvi, C.L., et al. (2024). Cell-type-specific and disease-associated expression quantitative trait loci in the human lung. Nat. Genet. *56*, 595–604. https://doi.org/10.1038/s41588-024-01702-0.

33. He, S., Bhatt, R., Brown, C., Brown, E.A., Buhr, D.L., Chantranuvatana, K., Danaher, P., Dunaway, D., Garrison, R.G., Geiss, G., et al. (2022). High-plex imaging of RNA and proteins at subcellular resolution in fixed tissue by spatial molecular imaging. Nat. Biotechnol. *40*, 1794–1806. https://doi.org/10.1038/s41587-022-01483-z.

34. Zhang, Y., Shen, J., Cheng, W., Roy, B., Zhao, R., Chai, T., Sheng, Y., Zhang, Z., Chen, X., Liang, W., et al. (2023b). Microbiota-mediated shaping of mouse spleen structure and immune function characterized by scRNA-seq and stereo-seq. J. Genet. Genomics *50*, 688–701. https://doi.org/10.1016/j.jgg.2023.04.012.

35. Harris, B.D., Lee, J., and Gillis, J. (2021). A meta-analytic single-cell atlas of mouse bone marrow hematopoietic development. Preprint at bioRxiv. https://doi.org/10.1101/2021.08.12.456098.

36. Ng, M.S.F., Kwok, I., Tan, L., Shi, C., Cerezo-Wallis, D., Tan, Y., Leong, K., Calvo, G.F., Yang, K., Zhang, Y., et al. (2024). Deterministic reprogramming of neutrophils within tumors. Science *383*, eadf6493. https://doi.org/10.1126/science.adf6493.

37. Gayoso, A., Steier, Z., Lopez, R., Regier, J., Nazor, K.L., Streets, A., and Yosef, N. (2021). Joint probabilistic modeling of single-cell multi-omic data with totalVI. Nat. Methods *18*, 272–282. https://doi.org/10.1038/s41592-020-01050-x.

38. Leong, T.L., and Bryant, V.L. (2021). B cells in lung cancer-not just a bystander cell: a literature review. Transl. Lung Cancer Res. *10*, 2830–2841. https://doi.org/10.21037/tlcr-20-788.

39. Laumont, C.M., and Nelson, B.H. (2023). B cells in the tumor microenvironment: Multi-faceted organizers, regulators, and effectors of anti-tumor immunity. Cancer Cell *41*, 466–489. https://doi.org/10.1016/j.ccell.2023.02.017.

40. Liu, Q., Hsu, C.Y., and Shyr, Y. (2022). Scalable and model-free detection of spatial patterns and colocalization. Genome Res. *32*, 1736–1745. https://doi.org/10.1101/gr.276851.122.

41. Kojima, Y., Mii, S., Hayashi, S., Hirose, H., Ishikawa, M., Akiyama, M., Enomoto, A., and Shimamura, T. (2024). Single-cell colocalization analysis using a deep generative model. Cell Syst. *15*, 180–192.e7. https://doi.org/10.1016/j.cels.2024.01.007.

42. Chen, J.W., and Dhahbi, J. (2021). Lung adenocarcinoma and lung squamous cell carcinoma cancer classification, biomarker identification, and gene expression analysis using overlapping feature selection methods. Sci. Rep. *11*, 13323. https://doi.org/10.1038/s41598-021-92725-8.

43. Wong, K.Y., Cheung, A.H.K., Chen, B., Chan, W.N., Yu, J., Lo, K.W., Kang, W., and To, K.F. (2022). Cancer-associated fibroblasts in nonsmall cell lung cancer: From molecular mechanisms to clinical implications. Int. J. Cancer *151*, 1195–1215. https://doi.org/10.1002/ijc.34127.

44. Cords, L., Engler, S., Haberecker, M., Rüschoff, J.H., Moch, H., de Souza, N., and Bodenmiller, B. (2024). Cancer-associated fibroblast phenotypes are associated with patient outcome in non-small cell lung cancer. Cancer Cell *42*, 396–412.e5. https://doi.org/10.1016/j.ccell.2023.12.021.

45. Zhou, B., Lawrence, T., and Liang, Y. (2021). The role of plasmacytoid dendritic cells in cancers. Front. Immunol. *12*, 749190. https://doi.org/10.3389/fimmu.2021.749190.

46. Pinto, A., Rega, A., Crother, T.R., and Sorrentino, R. (2012). Plasmacytoid dendritic cells and their therapeutic activity in cancer. Oncoimmunology *1*, 726–734. https://doi.org/10.4161/onci.20171.

47. Weng, Y., Yuan, J., Cui, X., Wang, J., Chen, H., Xu, L., Chen, X., Peng, M., and Song, Q. (2024). The impact of tertiary lymphoid structures on tumor prognosis and the immune microenvironment in non-small cell lung cancer. Sci. Rep. *14*, 16246. https://doi.org/10.1038/s41598-024-64980-y.

48. Sainz de Aja, J., Dost, A.F.M., and Kim, C.F. (2021). Alveolar progenitor cells and the origin of lung cancer. J. Intern. Med. *289*, 629–635. https://doi.org/10.1111/joim.13201.

49. Fukui, T., Shaykhiev, R., Agosto-Perez, F., Mezey, J.G., Downey, R.J., Travis, W.D., and Crystal, R.G. (2013). Lung adenocarcinoma subtypes based on expression of human airway basal cell genes. Eur. Respir. J. *42*, 1332–1344. https://doi.org/10.1183/09031936.00144012.

50. Li, F., Song, Q.Z., Zhang, Y.F., Wang, X.R., Cao, L.M., Li, N., Zhao, L.X., Zhang, S.X., and Zhuang, X.F. (2022b). Identifying the EMT-related signature to stratify prognosis and evaluate the tumor microenvironment in lung adenocarcinoma. Front. Genet. *13*, 1008416. https://doi.org/10.3389/fgene.2022.1008416.

51. Xiao, Y., Wang, Z., Gu, M., Wei, P., Wang, X., and Li, W. (2024). Cancer-associated fibroblasts: heterogeneity and their role in the tumor immune response. Clin. Exp. Med. *24*, 126. https://doi.org/10.1007/s10238-024-01375-3.

52. Hanley, C.J., Waise, S., Ellis, M.J., Lopez, M.A., Pun, W.Y., Taylor, J., Parker, R., Kimbley, L.M., Chee, S.J., Shaw, E.C., et al. (2023). Single-cell analysis reveals prognostic fibroblast subpopulations linked to molecular and immunological subtypes of lung cancer. Nat. Commun. *14*, 387. https://doi.org/10.1038/s41467-023-35832-6.

53. Gurzu, S., Turdean, S., Kovecsi, A., Contac, A.O., and Jung, I. (2015). Epithelial-mesenchymal, mesenchymal-epithelial, and endothelial-mesenchymal transitions in malignant tumors: An update. World J. Clin. Cases *3*, 393–404. https://doi.org/10.12998/wjcc.v3.i5.393.

54. Bergeron, A., El-Hage, F., Kambouchner, M., Lecossier, D., and Tazi, A. (2006). Characterisation of dendritic cell subsets in lung cancer micro-environments. Eur. Respir. J. *28*, 1170–1177. https://doi.org/10.1183/09031936.06.00114205.

55. Batra, H., and Antony, V.B. (2015). Pleural mesothelial cells in pleural and lung diseases. J. Thorac. Dis. *7*, 964–980. https://doi.org/10.3978/j.issn.2072-1439.2015.04.48.

56. 10x Genomics (2024b). Preview data: FFPE human lung cancer with Xenium multimodal cell segmentationhttps://www.10xgenomics.com/datasets/preview-data-ffpe-human-lung-cancer-with-xenium-multimodal-cell-segmentation-1-standardxeniumHumanMulti-TissueandCancerPanel(377genes),XeniumOnboardAnalysisv2.0.0.

57. Fennell, K.A., Vassiliadis, D., Lam, E.Y.N., Martelotto, L.G., Balic, J.J., Hollizeck, S., Weber, T.S., Semple, T., Wang, Q., Miles, D.C., et al. (2022). Non-genetic determinants of malignant clonal fitness at single-cell resolution. Nature *601*, 125–131. https://doi.org/10.1038/s41586-021-04206-7.

58. Holze, H., Talarmain, L., Fennell, K.A., Lam, E.Y., Dawson, M.A., and Vassiliadis, D. (2024). Analysis of synthetic cellular barcodes in the genome and transcriptome with BARtab and bartools. Cell Rep. Methods *4*, 100763. https://doi.org/10.1016/j.crmeth.2024.100763.

59. Reiss, D.J., Nakayama, Y., Weng, A.P., Stokes, M.E., Sehn, L., Steidl, C., Scott, D.W., Huang, C.C., and Gandhi, A.K. (2024). High-plex imaging and cellular neighborhood spatial analysis reveals multiple immune escape and suppression patterns in diffuse large B-cell lymphoma. Leukemia *38*, 1164–1168. https://doi.org/10.1038/s41375-024-02239-1.

60. den Haan, J.M., Mebius, R.E., and Kraal, G. (2012). Stromal cells of the mouse spleen. Front. Immunol. *3*, 201. https://doi.org/10.3389/fimmu.2012.00201.

61. Han, Y., Dong, Y., Yang, Q., Xu, W., Jiang, S., Yu, Z., Yu, K., and Zhang, S. (2018). Acute myeloid leukemia cells express ICOS ligand to promote the expansion of regulatory T cells. Front. Immunol. *9*, 2227. https://doi.org/10.3389/fimmu.2018.02227.

62. Liberzon, A., Birger, C., Thorvaldsdóttir, H., Ghandi, M., Mesirov, J.P., and Tamayo, P. (2015). The molecular signatures database (MSigDB) hallmark gene set collection. Cell Syst. *1*, 417–425. https://doi.org/10.1016/j.cels.2015.12.004.

63. Andreatta, M., and Carmona, S.J. (2021). UCell: Robust and scalable single-cell gene signature scoring. Comput. Struct. Biotechnol. J. *19*, 3796–3798. https://doi.org/10.1016/j.csbj.2021.06.043.

64. Aung, T.N., Monkman, J., Warrell, J., Vathiotis, I., Bates, K.M., Gavrielatou, N., Trontzas, I.P., Tan, C.W., Fernandez, A.I., Moutafi, M., et al. (2025). Spatial signatures for predicting immunotherapy outcomes using multi-omics in non-small cell lung cancer. Nat. Genet. *57*, 2482–2493. https://doi.org/10.1038/s41588-025-02351-7.

65. Hao, Y., Stuart, T., Kowalski, M.H., Choudhary, S., Hoffman, P., Hartman, A., Srivastava, A., Molla, G., Madad, S., Fernandez-Granda, C., and Satija, R. (2024). Dictionary learning for integrative, multimodal and scalable single-cell analysis. Nat. Biotechnol. *42*, 293–304. https://doi.org/10.1038/s41587-023-01767-y.

66. Elosua-Bayes, M., Nieto, P., Mereu, E., Gut, I., and Heyn, H. (2021). SPOTlight: seeded NMF regression to deconvolute spatial transcriptomics spots with single-cell transcriptomes. Nucleic Acids Res. *49*, e50. https://doi.org/10.1093/nar/gkab043.

67. Finak, G., McDavid, A., Yajima, M., Deng, J., Gersuk, V., Shalek, A.K., Slichter, C.K., Miller, H.W., McElrath, M.J., Prlic, M., et al. (2015). MAST: a flexible statistical framework for assessing transcriptional changes and characterizing heterogeneity in single-cell RNA sequencing data. Genome Biol *16*, 278. https://doi.org/10.1186/s13059-015-0844-5.

68. Wang, B., and Zou, H. (2018). Another look at distance-weighted discrimination. J. R. Stat. Soc. Series B Stat. Methodol. *80*, 177–198. https://doi.org/10.1111/rssb.12244.

69. Duong, T. (2007). ks: kernel density estimation and kernel discriminant analysis for multivariate data in R. J. Stat. Soft. *21*, 1–16. https://doi.org/10.18637/jss.v021.i07.

70. Haghverdi, L., Lun, A.T.L., Morgan, M.D., and Marioni, J.C. (2018). Batch effects in single-cell RNA-sequencing data are corrected by matching mutual nearest neighbors. Nat. Biotechnol. *36*, 421–427. https://doi.org/10.1038/nbt.4091.

71. Lun, A.T.L., Riesenfeld, S., Andrews, T., Dao, T., Gomes, T., and Marioni, J.C.; participants in the 1st Human Cell Atlas Jamboree (2019). EmptyDrops: distinguishing cells from empty droplets in droplet-based single-cell RNA sequencing data. Genome Biol *20*, 63. https://doi.org/10.1186/s13059-019-1662-y.

72. Dries, R., Zhu, Q., Dong, R., Eng, C.H.L., Li, H., Liu, K., Fu, Y., Zhao, T., Sarkar, A., Bao, F., et al. (2021). Giotto: a toolbox for integrative analysis and visualization of spatial expression data. Genome Biol. *22*, 78. https://doi.org/10.1186/s13059-021-02286-2.

73. Birk, S., Bonafonte-Pardàs, I., Feriz, A.M., Boxall, A., Agirre, E., Memi, F., Maguza, A., Fan, R., Castelo-Branco, G., Theis, F.J., et al. (2025). Quantitative characterization of cell niches in spatially resolved omics data. Nat. Genet. *57*, 927–938. https://doi.org/10.1038/s41588-025-02120-6.

74. Lun, A.T.L., Bach, K., and Marioni, J.C. (2016). Pooling across cells to normalize single-cell RNA sequencing data with many zero counts. Genome Biol. *17*, 75. https://doi.org/10.1186/s13059-016-0947-7.

75. Cable, D. spacexr: SpatialeXpressionR: Cell type identification and cell type-specific differential expression in spatial transcriptomics (2023). https://github.com/dmcable/spacexrrpackageversion2.2.1,commit5baf6393552e401857db1eb79ddb0af16ff15f84.

76. Song, Q., and Su, J. (2021). DSTG: deconvoluting spatial transcriptomics data through graph-based artificial intelligence. Brief. Bioinform. *22*, bbaa414. https://doi.org/10.1093/bib/bbaa414.

77. Xiao, Y., Hsiao, T.H., Suresh, U., Chen, H.I.H., Wu, X., Wolf, S.E., and Chen, Y. (2014). A novel significance score for gene selection and ranking. Bioinformatics *30*, 801–807. https://doi.org/10.1093/bioinformatics/btt671.

78. McDavid, A., Finak, G., and M, Y. MAST: Model-based Analysis of Single Cell Transcriptomics. (2024https://github.com/RGLab/MAST/rpackageversion1.30.0.

79. Human BioMolecular Atlas Program (2024). Azimuth: App for reference-based single-cell analysis. https://azimuth.hubmapconsortium.org/references/. .

80. Marron, J.S., Todd, M.J., and Ahn, J. (2007). Distance-weighted discrimination. J. Am. Stat. Assoc. *102*, 1267–1271. https://doi.org/10.1198/016214507000001120.

81. Wang, B., and Zou, H. kerndwd: Distance Weighted Discrimination (DWD) and Kernel Methods (2020). https://CRAN.R-project.org/package=kerndwdrpackageversion2.0.3.

CellPress
OPEN ACCESS

STAR★METHODS

KEY RESOURCES TABLE

REAGENT or RESOURCE	SOURCE	IDENTIFIER
**Deposited data**		
Human Lung Cell Atlas Core (ann_finest_level); Sikkema et al.[30]	CZ CELLxGENE	https://cellxgene.cziscience.com/collections/6f6d381a-7701-4781-935c-db10d30de293
10x Visium NSCLC dataset (18 lung sections, 9 individuals); De Zuani et al.[31]	BioStudies (EMBL-EBI)	Accession E-MTAB-13530
NanoString CosMx NSCLC dataset (8 lung sections, 5 patients); He et al.[33]	NanoString Technologies	https://nanostring.com/products/cosmx-spatial-molecular-imager/ffpe-dataset/nsclc-ffpe-dataset/
10x Xenium NSCLC dataset (FFPE, 377 genes, 161,894 cells); 10x Genomics	10x Genomics	https://www.10xgenomics.com/products/xenium-in-situ/preview-dataset-human-breast
scRNA-seq from healthy and fibrotic lungs (4 lineage-specific datasets); Natri et al.[32]	Gene Expression Omnibus (GEO)	Accession GSE227136
Mouse spleen scRNA-seq (SPF mice); Zhang et al.[11]; Zhang et al.[34]	CNGB Sequence Archive (CNSA), CNGBdb	Accession CNP0003930
Mouse bone marrow scRNA-seq (HSC atlas); Harris et al.[35]	Mouse HSC Atlas	https://gillisweb.cshl.edu/HSC_atlas/
Mouse spleen CITE-seq; Gayoso et al.[37]	GitHub	https://github.com/YosefLab/totalVI_reproducibility
Mouse neutrophil scRNA-seq; Ng et al.[36]	GEO	Accession GSE243466
AML mouse spleen Stereo-seq data; Holze et al.[58]	Zenodo	https://zenodo.org/records/10685805
AML mouse spleen scRNA-seq ('Mouse 2'); Fennell et al.[57]	GEO	Accession GSE161676
**Software and algorithms**		
PhiSpace R package (v 1.1.0)	This paper	https://github.com/jiadongm/PhiSpace; https://doi.org/10.5281/zenodo.19941499
spacexr R package (RCTD)	Cable et al.[12]	CRAN; https://github.com/dmcable/spacexr
cell2location Python package (v0.1.4)	Kleshchevnikov et al.[14]	https://github.com/BayraktarLab/cell2location
tacco Python package (v0.2.2)	Mages et al.[17]	https://github.com/simonwm/tacco
Seurat R package (v5.4.0)	Hao et al.[65]	CRAN; RRID: SCR_007322
SPOTlight R/Bioconductor package (v1.14.0)	Elosua-Bayes et al.[66]	Bioconductor
MAST R/Bioconductor package	Finak et al.[67]	Bioconductor; RRID: SCR_016340
kerndwd R package	Wang and Zou[68]	CRAN
ks R package	Duong[69]	CRAN
UCell R/Bioconductor package	Andreatta and Carmona[63]	Bioconductor
batchelor R/Bioconductor package (fastMNN)	Haghverdi et al.[70]	Bioconductor
DropletUtils R/Bioconductor package	Lun et al.[71]	Bioconductor
Giotto R package	Dries et al.[72]	https://giottosuite.com; RRID: SCR_018795
**Other**		
MSigDB Hallmark gene sets	Liberzon et al.[62]	https://www.gsea-msigdb.org/gsea/msigdb
BioRender (figure illustration)	BioRender	https://BioRender.com

EXPERIMENTAL MODEL AND STUDY PARTICIPANT DETAILS

This study is entirely computational and performs secondary analysis of previously published datasets; no new experimental data were generated.

Human tissue datasets

The 10x Visium NSCLC dataset consists of 18 lung tissue sections from 9 individuals (1 healthy donor, 8 NSCLC patients).[31] The CosMx NSCLC dataset consists of 8 tumorous lung tissue sections from 5 NSCLC patients.[33] The Xenium NSCLC dataset consists of one FFPE human NSCLC tissue section.[56] The scRNA-seq reference datasets from healthy and fibrotic lungs are from Natri et al.[32] Detailed participant information including age, sex and clinical characteristics are provided in the original publications. Ethical approvals and informed consent procedures are described in the respective original studies.

Mouse datasets

The Stereo-seq dataset is from an AML mouse with cellular lineage tracing (SPLINTR) barcodes.[58] The four scRNA-seq reference datasets used for the Stereo-seq case study were derived from mouse spleen,[34] bone marrow,[35] splenic immune cells (CITE-seq)[37] and neutrophils.[36]

METHOD DETAILS

We denote the R reference datasets by $(X_{\text{ref},r}, Y_{\text{ref},r})$, $r = 1, \ldots, R$, where each $X_{\text{ref},r}$ is a gene by cell scRNA-seq gene expression matrix of normalised values (Figure 1A). The phenotype matrix $Y_{\text{ref},r}$ is a dummy matrix that represents the K_r cell type labels of cells in $X_{\text{ref},r}$. That is, each row of $Y_{\text{ref},r}$ represents a cell type and a cell is assigned the value 1 if it belongs to that cell type or -1 otherwise (Figure 1A).

We denote the query ST dataset by $(X_{\text{query}}, S_{\text{query}})$, where X_{query} is the query gene expression matrix with rows representing genes and columns representing spatial cell-like objects (segmented cells, multicellular spots or transcript bins), and where S_{query} contains the coordinates of the cell-like objects (x,y).

Multi-reference continuous phenotyping

The main steps for deriving the Φ-Space ST cell type scores based on multiple references are as follows.

1. Gene selection. For each reference dataset $(X_{\text{ref},r}, Y_{\text{ref},r})$, we use the variable selection method based on partial least squares (PLS) regression, as described in Mao et al.,[20] to select the most useful genes $\mathcal{G}_r$ for predicting the cell type $Y_{\text{ref},r}$. That is, each $\mathcal{G}_r$ contains the genes with the largest absolute values of PLS regression coefficients for predicting $Y_{\text{ref},r}$.
2. PLS model training (Figure 1B1). For the rth reference, we train a PLS regression model[26,27] using only genes shared by $\mathcal{G}_r$, the selected genes for reference r and $\mathcal{G}$, all genes sequenced in the query. We estimate a low-rank regression coefficient matrix B_r of size $(|\mathcal{G}_r| \times K_r)$ to fit the regression model $Y_{\text{ref},r} = \tilde{X}_{\text{ref},r} B_r$, where K_r denotes the number of cell types in the rth reference and $\tilde{X}_{\text{ref},r}$ denotes the rth gene expression matrix using only selected genes. The rank of B_r is equal to the number of PLS components, by default set to K_r.
3. Prediction (Figure 1B2). We compute the continuous predicted cell type scores $\hat{Y}_{\text{query},r}$ as a rescaled version of $\tilde{X}_{\text{query}} B_r$, where $\tilde{X}_{\text{query}}$ denotes the query gene expression matrix using only selected genes; see Mao et al.[20] for details of the rescaling. The final predicted cell type scores $\hat{Y}_{\text{query}}$ is the concatenation of all $\hat{Y}_{\text{query},r}$ and has size $\left(N \times \sum_{r=1}^{R} K_r\right)$, where N is the number of cell-like objects in the query. That is, the number of columns of $\hat{Y}_{\text{query}}$ is equal to the sum of cell types defined in all R references.

We call the predicted cell type score matrix $\hat{Y}_{\text{query}}$ the *phenotype space embeddings* of the query cell-like objects. Compared to hard cell type classification of cell type which assigns a unique cell type label to each query cell-like object, the phenotype space embedding contains much richer information regarding the spatial distribution of cell states and can be used for various downstream analyses described below.

Since each reference dataset is processed independently, cell types that appear in multiple references produce separate score columns in the phenotype space embedding (e.g., CD4 T cells from both spleen and bone marrow CITE-seq references in the Stereo-seq case study; see Table 1). No merging or averaging is applied: the column 'CD4 T(Spleen)' captures the query's similarity to CD4 T cells as characterised by spleen-derived gene expression patterns, while 'CD4 T(CITE)' captures the similarity as defined by CITE-seq reference markers. Cell types unique to a single reference contribute columns that no other reference can provide, enriching the phenotype space with specialised biological information. This design preserves the nuance that the same broad cell type label may reflect different transcriptional programs across tissue contexts, and allows downstream analyses to determine which distinctions are biologically relevant. Genuinely duplicated cell types can be dealt with by either manual deletion or by PCA (see 'Spatial niche identification' below). See Figures S14 and S15; Table S2 for a quantitative evaluation of the cross-reference consistency of Φ-Space ST scores.

Spatial niche identification

Φ-Space ST provides a simple approach for spatial niche identification (Figure 1C1), a major analytical task in ST data analysis to partition the whole spatial domain into cell communities.[10,73] We conduct k-means clustering based on the PCs of the Φ-Space ST cell type scores, where the number of PCs is selected via scree plot. Using PCs rather than the original cell type scores allows

to denoise some cell type scores that may not be spatially variable enough to contribute toward niche identification. This PCA step also reduces redundant information and possible co-linearity introduced by duplicated cell types from multiple references.

Data preprocessing

All datasets were preprocessed using the `PhiSpace` R package[29] as follows. The Human Lung Cell Atlas ('Core') dataset[30] was downloaded and subsampled using `subsample` (10% per cell type, minimum 50 cells), then log1p normalised using `logTransf`. Feature importance scores were obtained using `tunePhiSpace`. The 10x Visium NSCLC samples[31] were downloaded in h5 format, read using `SeuratLoad10X_Spatial`, converted to SingleCellExperiment (SCE) objects, and log1p normalised using `logTransf`. The four scRNA-seq datasets from healthy and fibrotic lungs[32] were subsampled (10% per cell type, minimum 50 cells) and log1p normalised using `logTransf`. Feature importance scores were obtained using `tunePhiSpace`. The CosMx NSCLC data[33] was downloaded as a `Giotto`[72] object, converted to SCE objects, and log1p normalised using `logTransf`. The Xenium NSCLC data[56] was loaded using `read10xCounts()` from the `DropletUtils` R package. After removing control probes and filtering cells with zero counts, the dataset comprised 377 genes across 161,894 cells. Expression values were normalised using `logTransf`. Pathologist annotations were assigned via point-in-polygon mapping from the accompanying GeoJSON file. Mouse spleen scRNA-seq data[34] was scran normalised.[74] Mouse bone marrow data[35] was subsampled (10% per cell type, minimum 50 cells) and rank normalised using `RankTransf`, as raw counts were unavailable. Mouse spleen CITE-seq data[37] was filtered to retain spleen cells only; mild batch effects between two experimental batches were corrected using `batchelorfastMNN`,[70] and the reconstructed log-normalised data were used. Mouse neutrophil scRNA-seq data[36] was scran normalised.[74] For the AML mouse spleen scRNA-seq data,[57] normalisation was matched to each reference: scran normalisation when paired with the Spleen,[34] CITE-seq[37] or Neutro[36] references; rank normalisation when paired with the bone marrow ref.[35]; and log1p normalisation (`logTransf`) when used as a reference to annotate the Stereo-seq data. The Stereo-seq bin50 data[58] was log1p normalised using `logTransf`.

Parameter settings for case studies

In all case studies the number of PLS components was set to *ncomp* = *K*, with *K* denoting the total number of cell types in the ref.[20] In the CosMx case study, the query data contained 960 genes (951 shared with the reference); no gene selection was performed and all 951 shared genes were used. For the Visium and Stereo-seq case studies, the top *nfeat* most useful features were selected: *nfeat* = 500 for Visium; for Stereo-seq, *nfeat* = 500 for all references except the Neutro reference (*nfeat* = 600), yielding approximately 3,000 training genes in both cases. In the Xenium case study, the same four references as the CosMx case were used; since the Xenium gene panel contained only 377 genes, all shared genes were used without feature selection.

Deconvolution benchmark setup

For the quantitative benchmark shown in Figure S1, each competing method used the same feature selection strategy as Φ-Space ST to ensure a fair comparison. RCTD[12] was implemented using the R package `spacexr`[75] with unnormalised counts. Cell2location[14] was implemented using the Python package `cell2location` (v0.1.4), following the official vignette with `q05_abundance` as the predicted cell type abundance. TACCO[17] was implemented using the Python package `tacco` (v0.2.2) via `tacco.tl.annotate` with default parameters. Seurat label transfer[65] was implemented in R via `FindTransferAnchors` and `TransferData`. SPOTlight[66] was implemented via the `SPOTlight` function with marker genes identified by `FindAllMarkers`. For DSTG,[76] metric values from Li et al.[15] were used directly. For the Visium case study comparison (Figure 2), we defined *meaningful markers* of a target cell type *C* in tissue *T* by method *M* as the intersection of: (i) the *spatial differentially expressed (DE) genes*, defined as the top 100 genes (ranked by importance score[77]) differentially expressed in the top 20% of spots with higher *C* identity according to method *M*, identified by MAST[78]; and (ii) the *cell type marker genes*, defined as the 100 genes comprising the 10 Azimuth markers[79] plus the 90 genes most positively correlated with those markers. For each NSCLC Visium sample, *favourable prognosis scores* were calculated based on NSCLC prognosis markers from Chen et al.[42] For LUSC samples, the score was the ratio of total *CELSR2* count to mean total gene count. For LUAD samples, the score was the ratio of mean total count for 4 favourable markers (*PERP*, *ELFN2*, *ARHGAP12*, *QSOX1*) to mean total count for 6 unfavourable markers (*KRT17*, *KRT6A*, *S100A2*, *TRIM29*, *REPS1*, *GPC1*).

Stereo-seq spatial bin clustering

Stereo-seq bins were clustered based on gene expression by computing the top 15 PCs of the log1p-transformed gene expression matrix and applying *k*-means clustering (R function `kmeans`, `algorithm = "Lloyd"`, `iter.max = 500`, `nstart = 50`, *k* = 8 following[58]). For Φ-Space -based clustering, the same *k*-means specifications were applied to the top 15 PCs of the Φ-Space scores.

Meta-clone identification

AML meta-clones were identified from the barcode composition of spatial bins as follows.

1. *Barcode-seq matrix.* A sparse matrix was generated with spatial bins as columns and individual AML barcodes as rows; each entry represents the count of a specific barcode in a specific bin.
2. *Low-abundance barcode filtering.* Barcodes with fewer than 50 total counts were removed; 35 of 370 barcodes were retained after filtering.

3. *Local smoothing.* For each bin, the mean counts of neighboring bins within a 500 μm diameter were computed as augmented counts. The clustering results were insensitive to diameter values between 100 μm and 1000 μm.
4. *k-means clustering.* Bins were clustered based on augmented barcode counts; k was varied from 2 to 10 (Figure S10) and the $k = 8$ result is presented in the main text.

Gene expression signature scoring

To characterise functional programs in the Stereo-seq AML mouse spleen sample, we scored each of the 15,454 spatial bins for 11 biologically relevant gene expression signatures from the MSigDB Hallmark collection[62] using UCell.[63] The selected signatures covered proliferation (G2M checkpoint, E2F targets), oncogenic signaling (MYC targets), inflammatory signaling (TNF-α via NF-κB, IFN-α response, IFN-γ response, IL6/JAK/STAT3), metabolism (oxidative phosphorylation), apoptosis, hypoxia and complement. Gene overlap between the Hallmark gene sets and the Stereo-seq transcriptome was high (94–99% per signature).

Cross-reference consistency analysis

For the Stereo-seq AML mouse spleen case study, we assessed the empirical consistency of Φ-Space ST scores for overlapping cell types across the four reference datasets. Spearman rank correlations were computed for overlapping or closely related cell type pairs across the 5,595-cell intermediate scRNA-seq dataset. Spatial consistency was further assessed by comparing side-by-side spatial heatmaps and spatial Spearman correlations for representative overlapping cell type groups in the Stereo-seq data.

QUANTIFICATION AND STATISTICAL ANALYSIS

Niche-specific enriched cell types

Once spatial niches have been identified, we further the analysis to identify niche-specific enriched cell types (Figure 1C2). We perform discriminant analysis using one of the following two methods.

- *Distance-weighted discrimination (DWD) for two niches*.[80] DWD is a powerful method for binary classification. We use DWD to identify a linear combination of Φ-Space cell type scores such that cell-like objects from the two niches can be best separated. We implemented DWD using the `kerndwd` package.[68,81]
- *Partial least squares discriminant analysis (PLS-DA) for multiple niches*.[27] PLS-DA is a flexible method for multi-class classification. We use PLS-DA to identify linear combinations of Φ-Space cell type scores such that cell-like objects from multiple niches can be best separated. We implemented PLS-DA using the `PhiSpace` package.[20]

Note that PLS-DA can also handle the case involving two niches. However, we found that the DWD loadings seemed to provide clearer biological interpretation in our case studies. Hence we opted for DWD for the two-niche case. We then rank the cell types according to their contribution toward predicting a given spatial niche, based on either the DWD or PLS-DA loadings. We define spatially enriched cell types as those that contribute most positively to predicting a specific niche.

Cell-type co-presence

To characterise the pairwise co-presence of different cell types within a spatial region (Figure 1C3), we compute the co-presence matrix as follows. Given a spatial region (e.g., a spatial niche or tissue sample) containing N cell-like objects annotated with K cell types, we extract the $N \times K$ matrix of normalised Φ-Space ST scores for that region. The co-presence matrix is then defined as the $K \times K$ Spearman correlation matrix of these scores. Each entry r_{ij} of the correlation matrix quantifies the pairwise co-occurrence of cell types i and j: a positive value indicates that the two cell types tend to co-occur in the same cell-like objects, while a negative value indicates that the cell types are mutually exclusive.

To compare co-presence patterns across spatial regions, we vectorise the lower triangle of each $K \times K$ co-presence matrix, yielding a $(K(K\text{-}1)/2)$-dimensional vector per region. Stacking these vectors across M regions produces an $M \times K(K\text{-}1)/2$ matrix, to which we apply PCA. The resulting PCs identify linear combinations of cell type pairs whose co-presence patterns vary the most across regions. Importantly, when a PC loading highlights multiple cell type pairs simultaneously, this reveals coordinated multi-cell-type co-presence modules, i.e., groups of cell types whose pairwise co-presences rise and fall together across spatial regions. These modules may capture higher-order co-presence structure. We can also perform discriminant analysis using DWD or PLS-DA to identify cell type pairs whose co-presence distinguishes between disease conditions.

Enriched cell types in AML meta-clones

To identify enriched cell types in each AML meta-clone compared to the background (non-barcoded bins), we applied a hypothesis-testing approach. For each cell type, Φ-Space ST scores of bins in a given meta-clone were compared to scores of non-barcoded bins using a two-sample Wilcoxon rank-sum test (`statswilcox.test`). A mean fold change (MFC) was calculated as:

$$\text{MFC} = \frac{\text{mean cell type score of meta} - \text{clone} - \text{mean cell type score of background}}{\text{standard deviation of cell type score of background}}.$$

The significance score for a given cell type was then defined as $-log(p) \times MFC$, where p is the Wilcoxon test p-value. The sign of the significance score indicates enrichment (positive) or depletion (negative). The five cell types with the largest positive significance scores (all with adjusted p-value < 0.05) were selected as the most enriched cell types for each meta-clone.

Meta-clone-specific gene expression signatures

For each gene expression signature, UCell scores in a given meta-clone were compared to scores in background non-barcoded bins using a two-sample Wilcoxon rank-sum test. Signature enrichment scores were calculated as mean fold change multiplied by $-log_{10}(p)$, where p is the Wilcoxon test p-value. Spearman correlations between Φ-Space ST cell type scores and gene signature scores were computed across all spatial bins to link inferred cell type identity to cellular state.

Cell Reports Methods, Volume *6*

Supplemental information

Φ-Space ST: A platform-agnostic method to identify cell states in spatial transcriptomics studies

Jiadong Mao, Jarny Choi, and Kim-Anh Lê Cao

S1 Supplementary figures

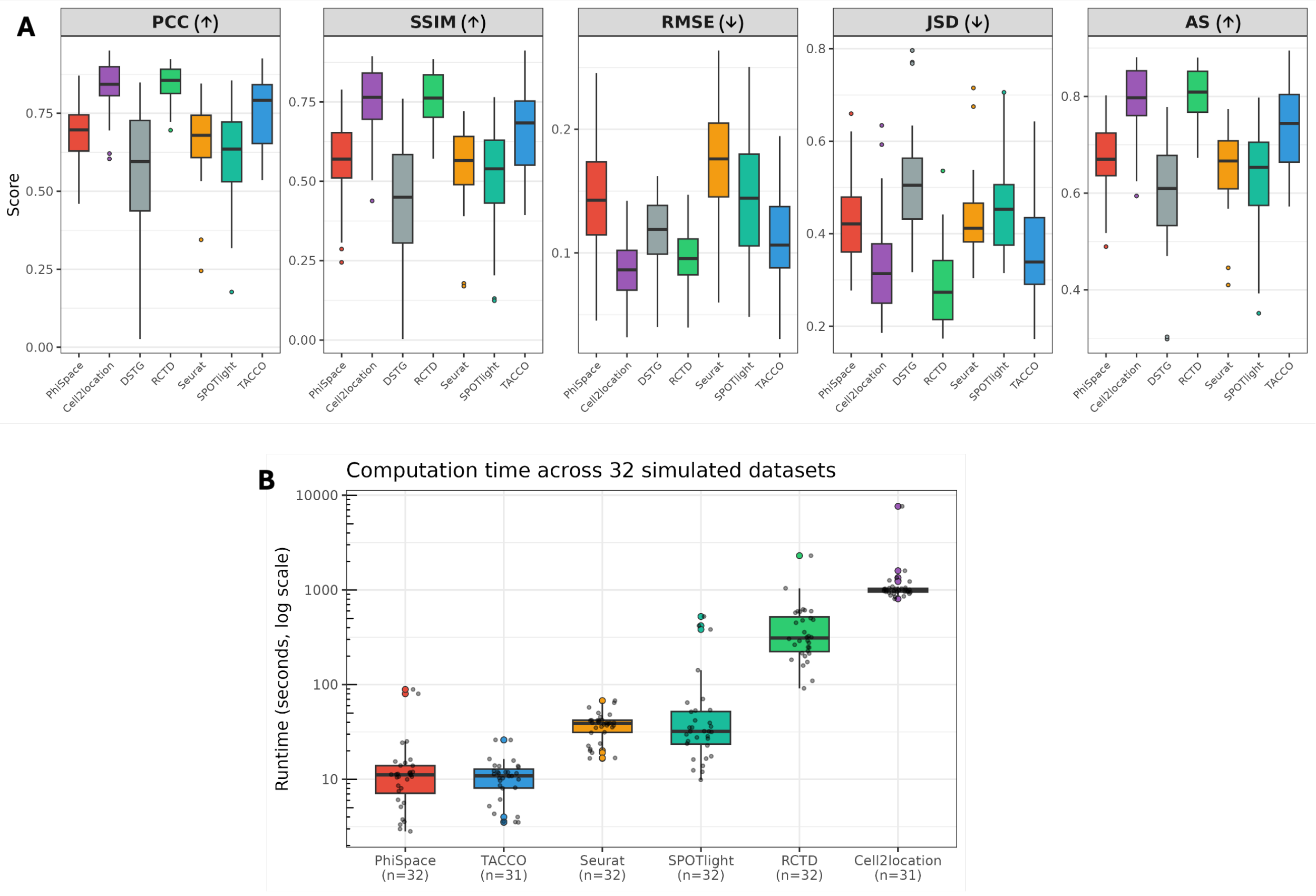

Fig S1: Benchmarking Φ-Space ST against 6 other methods, related to Figure 2. A: Following Li et al.[1], we used four metrics measuring the performance of 7 methods for cell type deconvolution. ↑: higher metric means better method; ↑: lower metric means better method. PCC: Pearson correlation coefficient; SSIM: structural similarity index; RMSE: root mean square error; JSD: Jensen-Shannon divergence. **B:** Runtime of six methods over simulated datasets.

S2 Supplementary tables

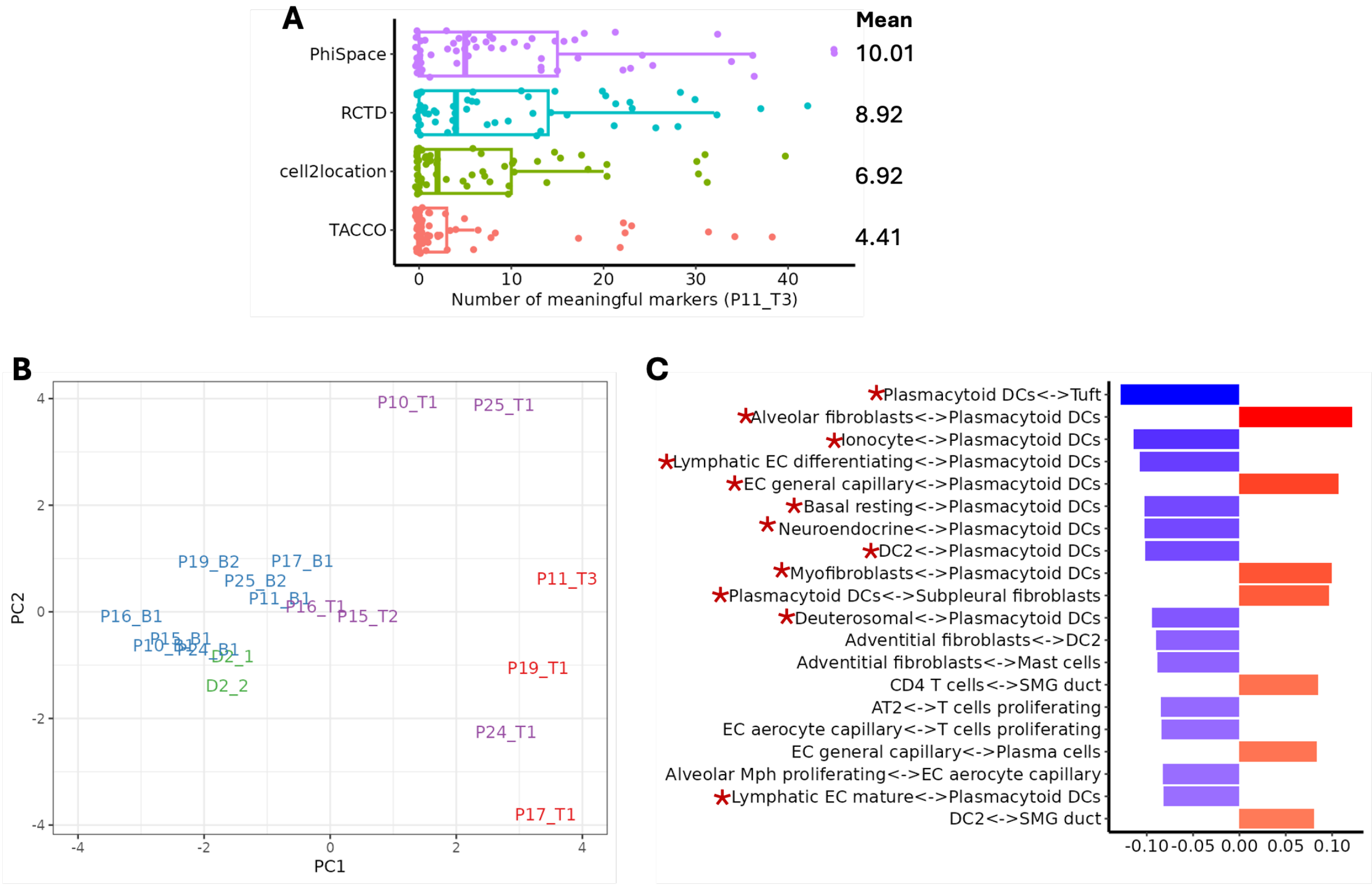

Fig S2: Immune cell type interactions in 10x Visium lung cancer samples, related to Figure 2. A: Boxplot comparing number of meaningful markers for the prediction of all cell types in P11_T3. **B:** Similar to Fig 2A, but now focusing on immune cell types, we filtered out correlations between non-immune cell types from the correlation matrices and recomputed their first two PCs. The separation between tumorous and non-tumorous tissues was largely retained compared to Fig 2A. **C:** Top 20 cell type interactions involving immune cell types contributing to PC1: 12/20 interactions (asterisks) involved plasmacytoid dendritic cells (pDCs).

References

[1] Bin Li, Wen Zhang, Chuang Guo, Hao Xu, Longfei Li, Minghao Fang, Yinlei Hu, Xinye Zhang, Xinfeng Yao, Meifang Tang, Ke Liu, Xuetong Zhao, Jun Lin, Linzhao Cheng, Falai Chen, Tian Xue, and Kun Qu. Benchmarking spatial and single-cell transcriptomics integration methods for transcript distribution prediction and cell type deconvolution. *Nat. Methods*, 19(6):662–670, 2022. doi: 10.1038/s41592-022-01480-9.

[2] José E Chacón and Tarn Duong. *Multivariate kernel smoothing and its applications.* CRC Press, London, England, 1st edition edition, 2020.

[3] Tarn Duong. *ks: Kernel Smoothing*, 2024. URL `https://CRAN.R-project.org/package=ks`. R package version 1.14.2.

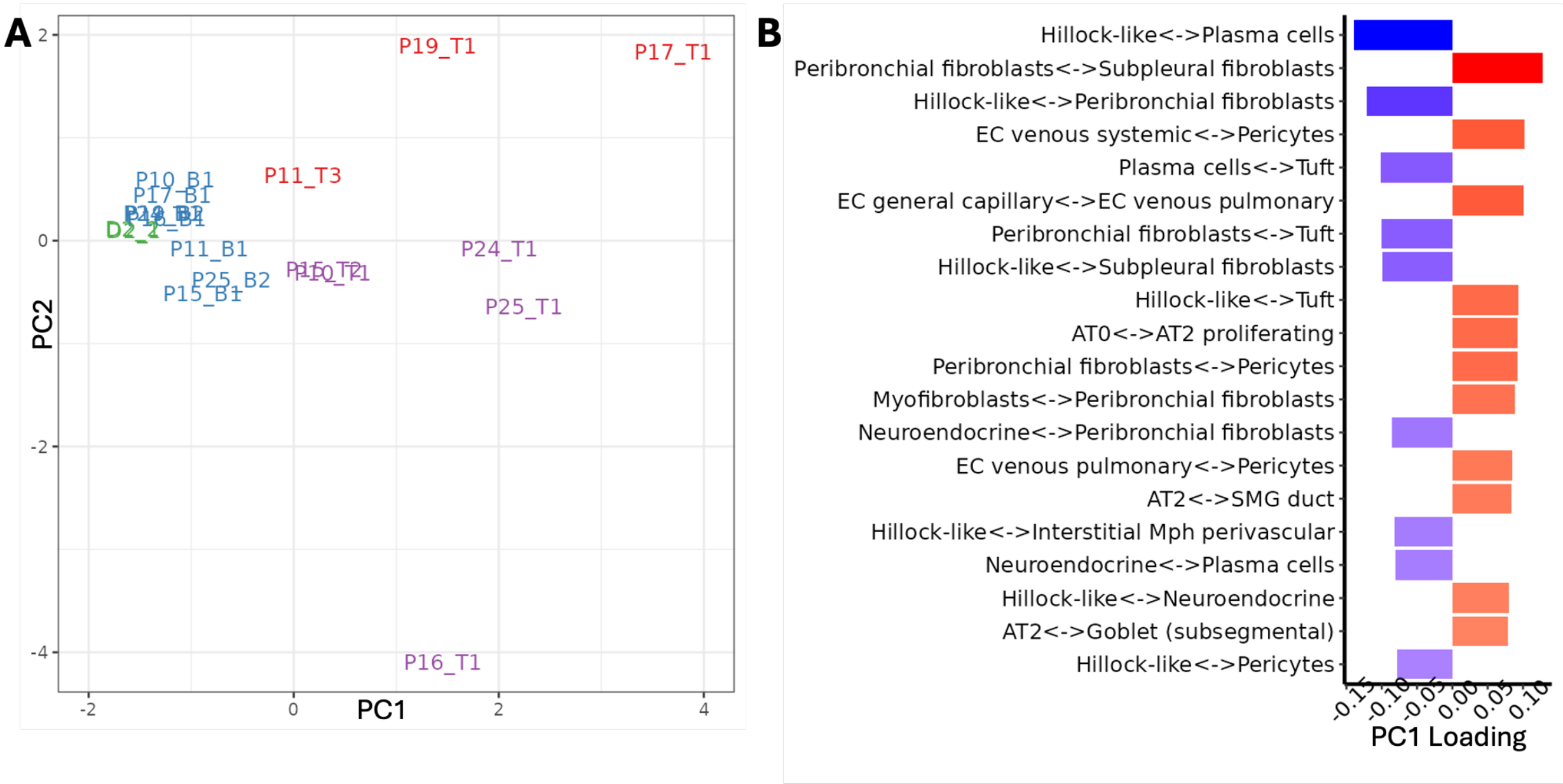

Fig S3: Cell type co-presence analysis using RCTD deconvolution results, related to Figure 2. A: The first two principal components (PCs) of cell type co-presence matrices derived using RCTD deconvolution results. We observed separation of different disease conditions. However, two PCs were needed for separating LUAD, LUSC and non-cancerous samples instead of just PC1 as in the Φ-Space ST results in Fig 2D. Moreover, neither of the RCTD PCs here seems to correspond to prognosis (Fig 2E). **B:** Top 20 loading values (absolute value) contributing to PC1. Fibroblasts still plays a prominant role, similar to the Φ-Space ST results as in Fig 2F.

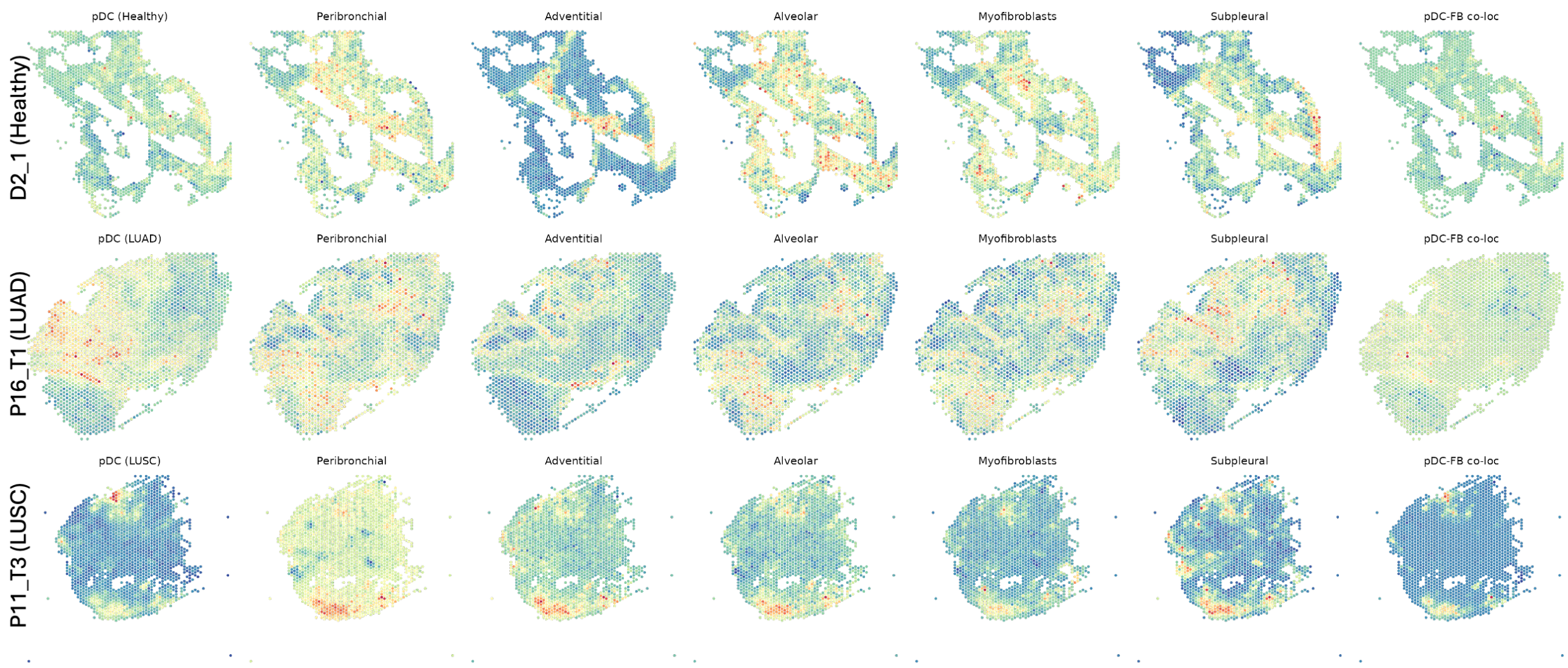

Fig S4: Spatial heatmaps for pDC and fibroblasts in 3 representative Visium samples, related to Figure 2. Spatial heatmaps of pDC score, fibroblast subtype scores and pDC–FB co-presence score (Spearman correlation) for representative healthy, LUAD and LUSC samples.

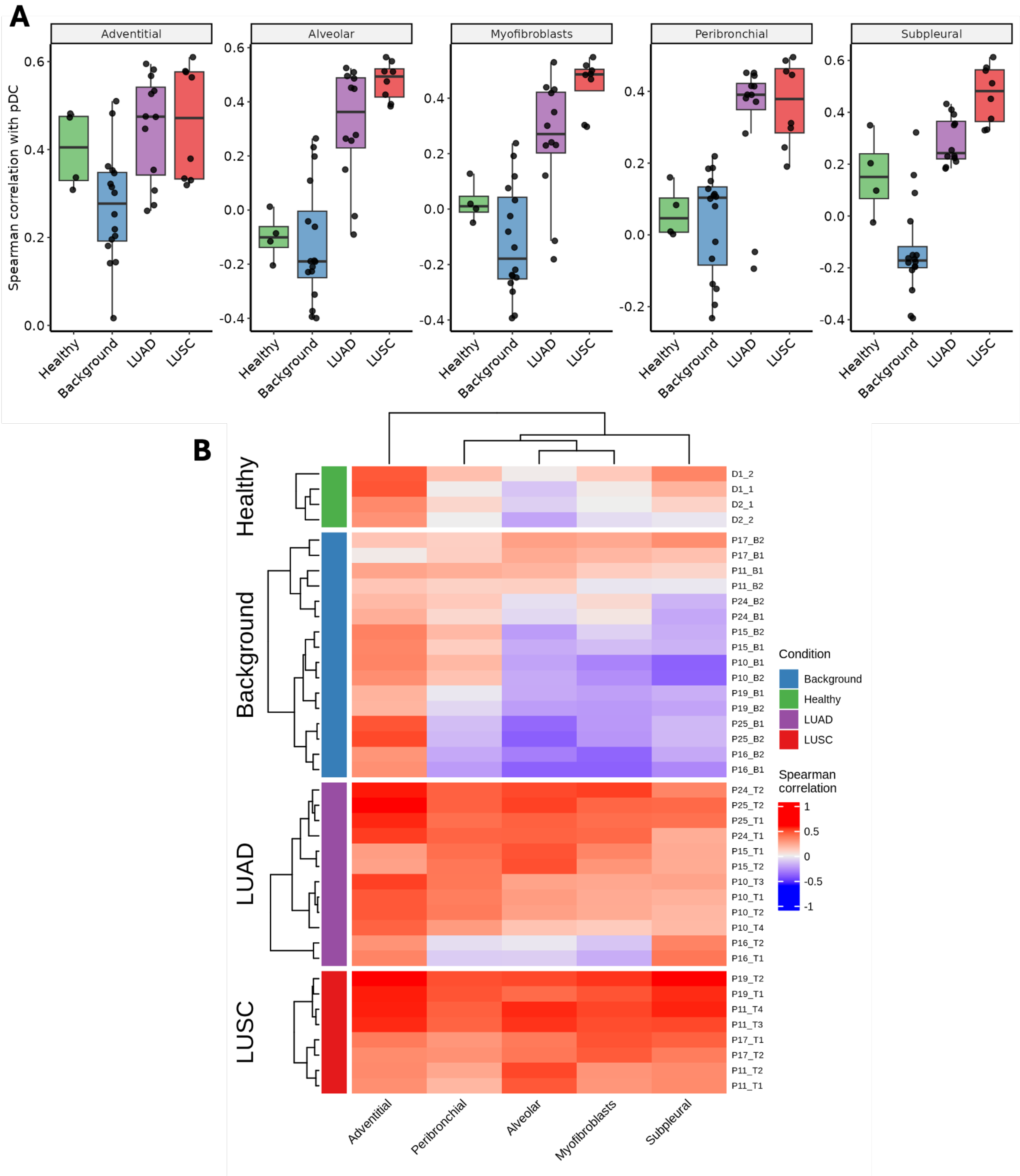

Fig S5: Co-localisation of pDC and fibroblasts in all 40 Visium samples, related to Figure 2. **A:** Boxplots for pDC–fibroblast Spearman correlation by condition (Healthy, Background, LUAD, LUSC) for each fibroblast subtype. **B:** Heatmap for sample by fibroblast subtype Spearman correlations with pDC, grouped by condition.

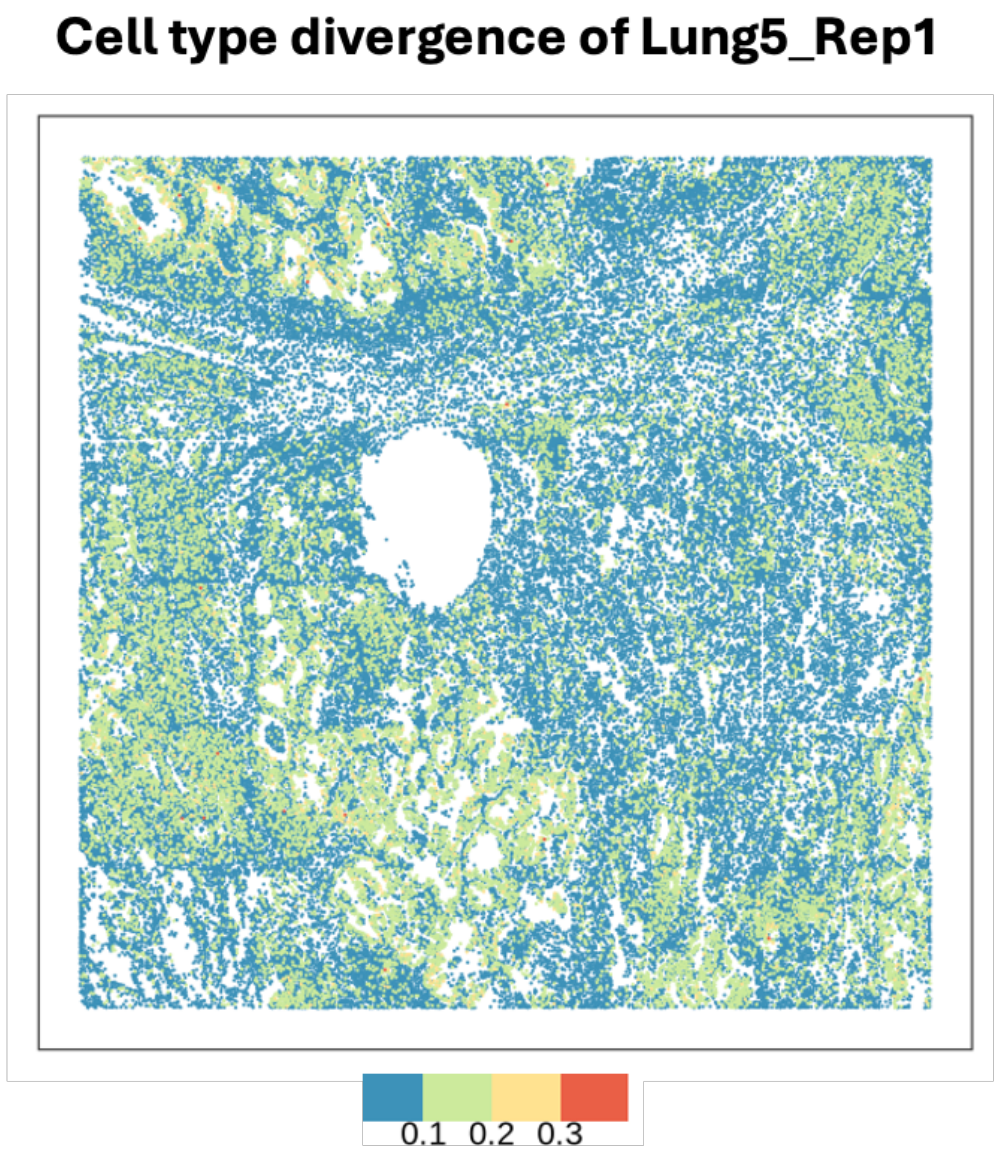

Fig S6: Cell type divergence in the CosMx Lung5_Rep1 sample, related to Figure 3. Cell type divergence of all segmented cells in Lung5_Rep1, computed based on their phenotype space embeddings derived using Φ-Space ST. The tumour and lymphoid structure domains in Fig 3A showed higher divergence.

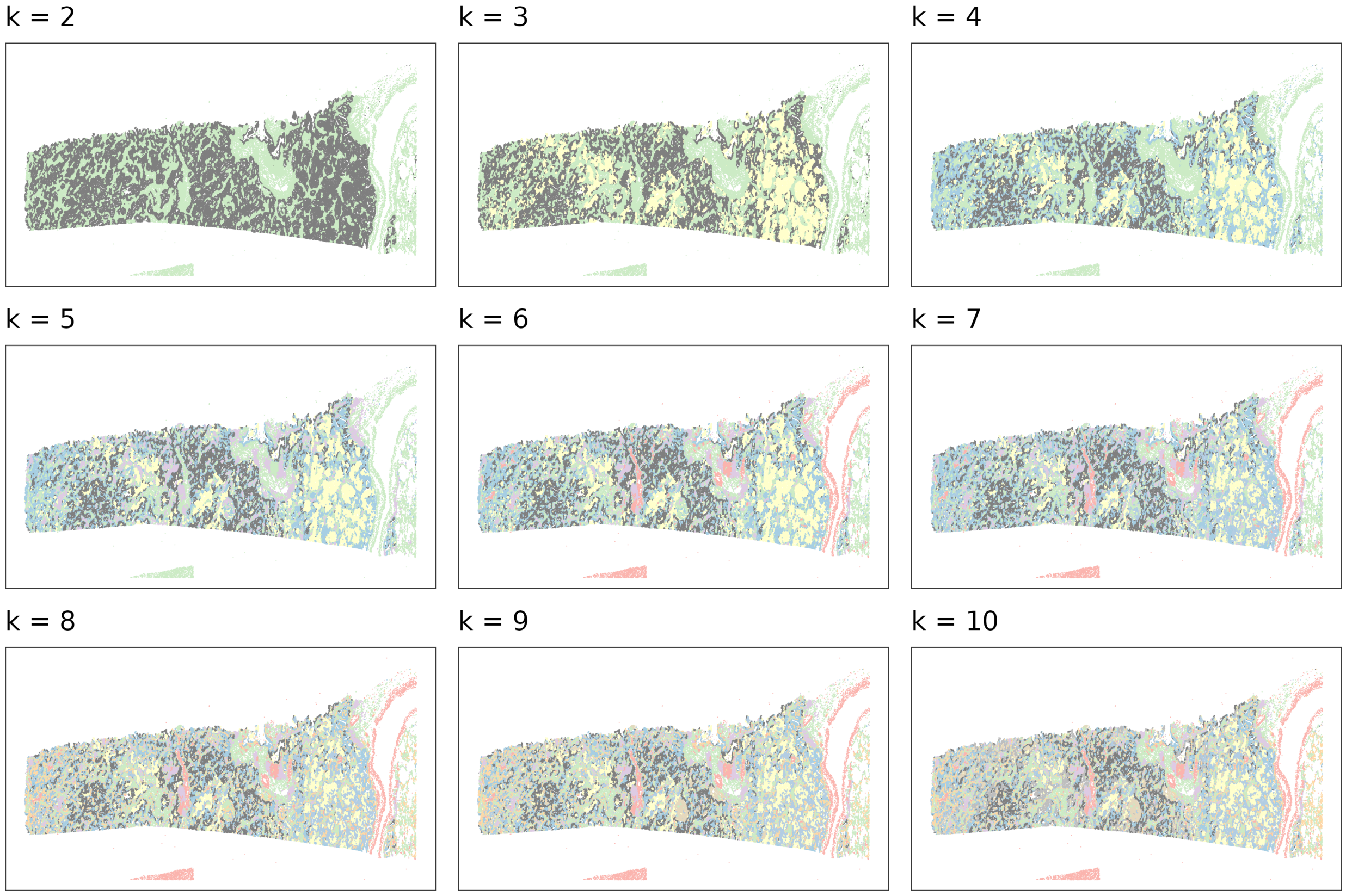

Fig S7: Spatial niches for Xenium lung sample, related to Figure 4. When identifying spatial niches for the Xenium lung sample, we applied k-means to spatially smoothed Φ-Space ST scores and varied the number of clusters from 2 to 10. Partition of the whole tissue section became virtually undistinguishable after number of clusters increased to 8. Hence we chose to present the 8-niche result in the main text.

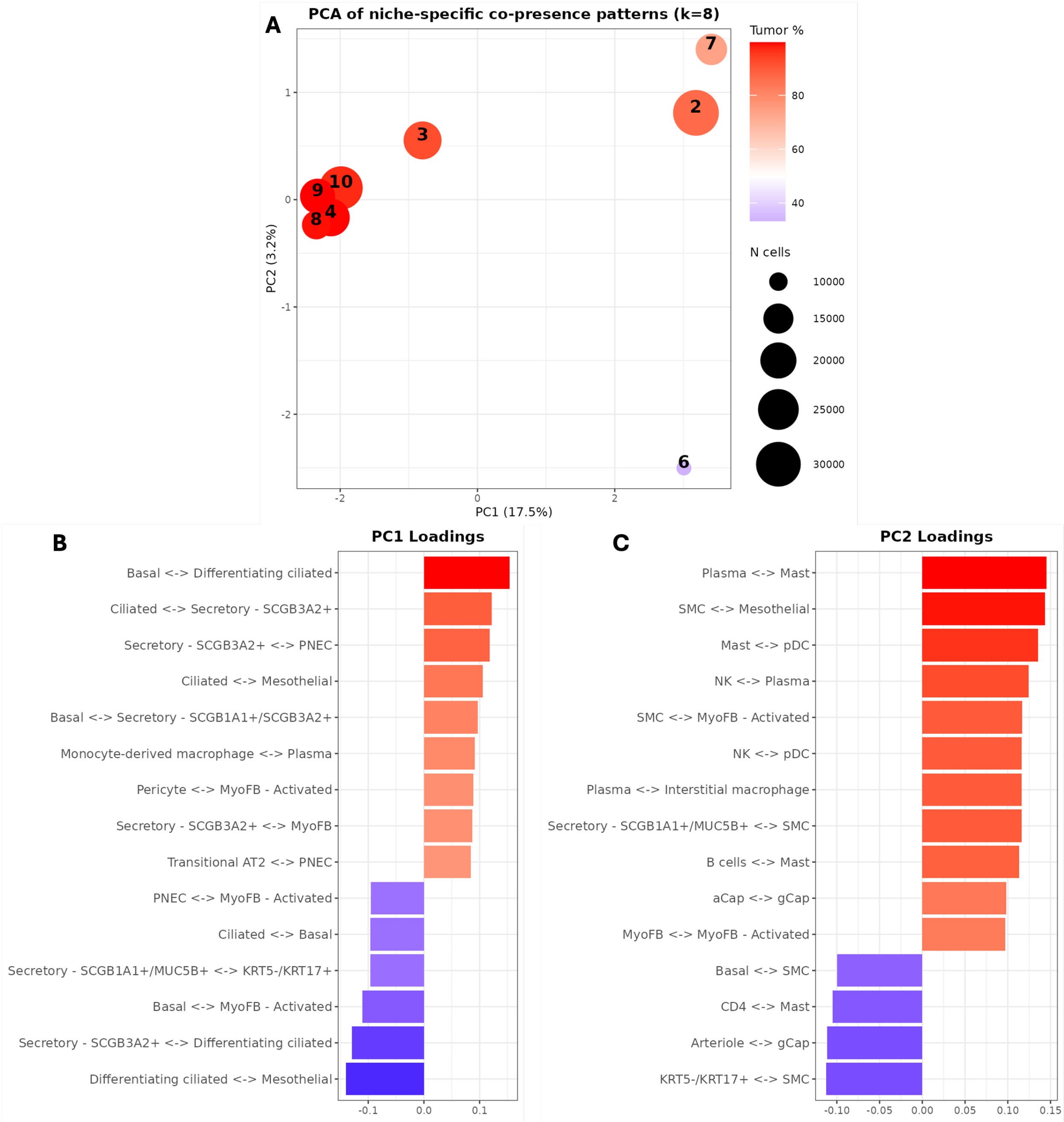

Fig S8: Cell type co-presence analysis for Xenium NSCLC sample, related to Figure 4. A: The first two principal components (PCs) of cell type co-presence matrices derived from individual niches in the Xenium NSCLC sample. We observed tumour high niches (3, 4, 8, 9, 10), intermediate niches (2, 7) and tumour low niche (6) occupying three corners on the PC plot. **B&C:** Top 15 loading values (absolute value) contributing to PC1 and PC2.

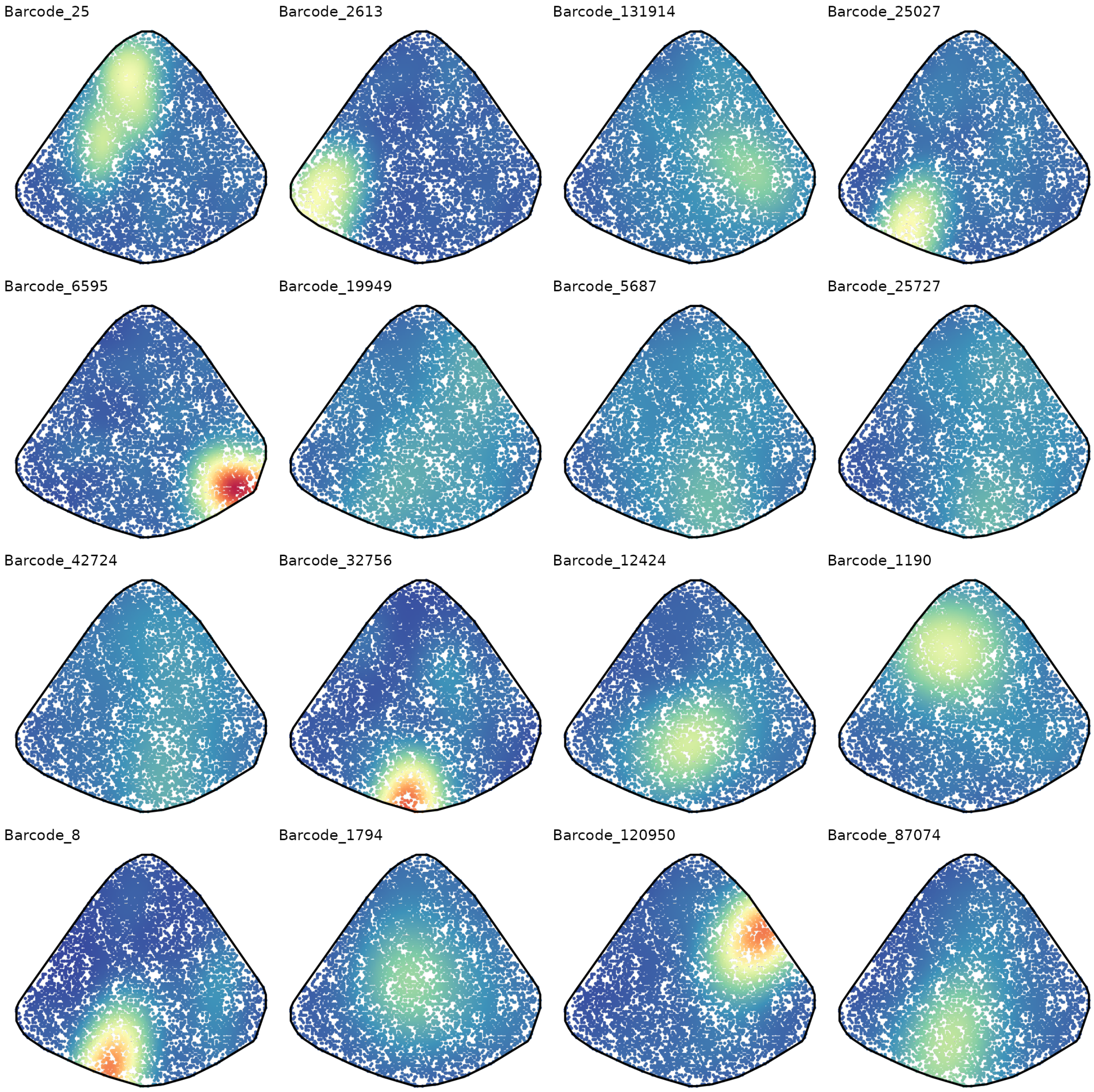

Fig S9: Spatial distributions of most populous AML barcodes, related to Figure 5. Each subfigure showed the spatial distribution of a specific AML barcode, where each spot represented a barcoded Stereo-seq bin. The spatial distribution of each barcode was estimated using a 2-dimensional kernel density estimator[2,3]. The warmer the colour was, the higher the estimated density value.

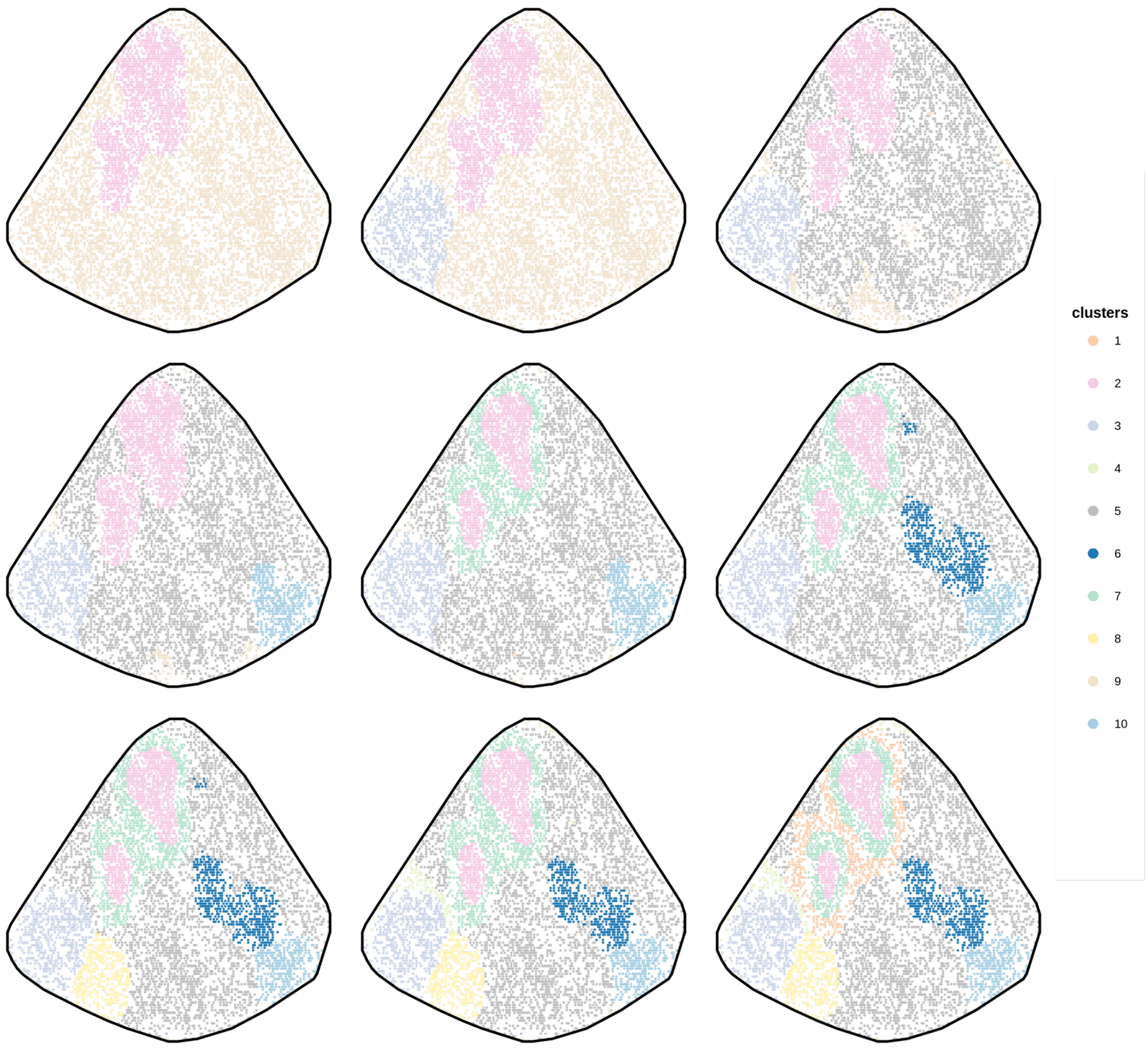

Fig S10: Clustering Stereo-seq bins according to their barcode composition, related to Figure 5. We varied the number of clusters k from 2 to 10 in the meta-clone identification pipeline (see STAR Methods). Each subfigure above represented a specific choice of k.

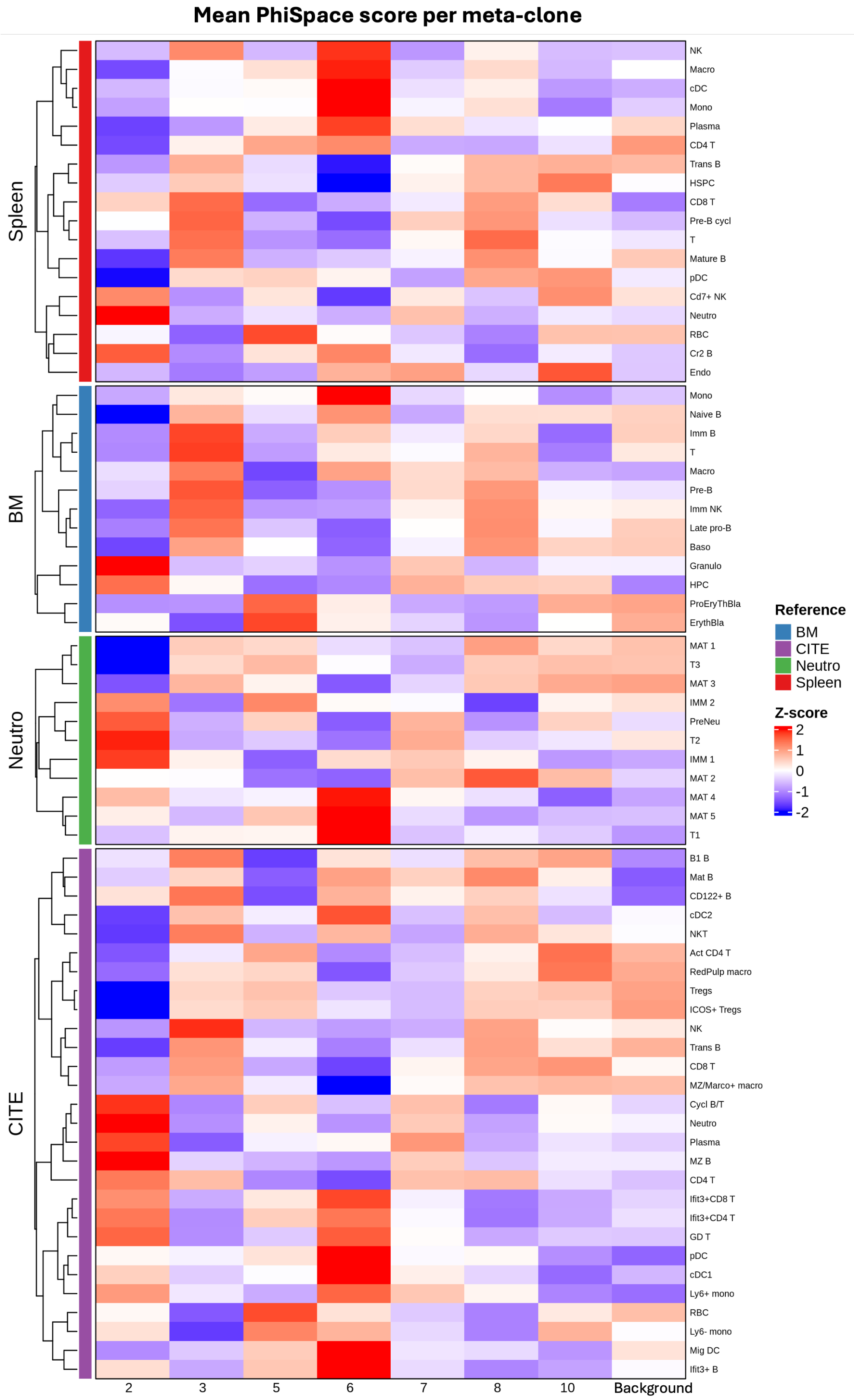

Fig S11: Cell type composition in meta-clones, related to Figure 5. Heatmap of mean Φ-Space scores (z-scaled) for each meta-clone and background non-barcoded bins. Cell types (rows) grouped by reference dataset.

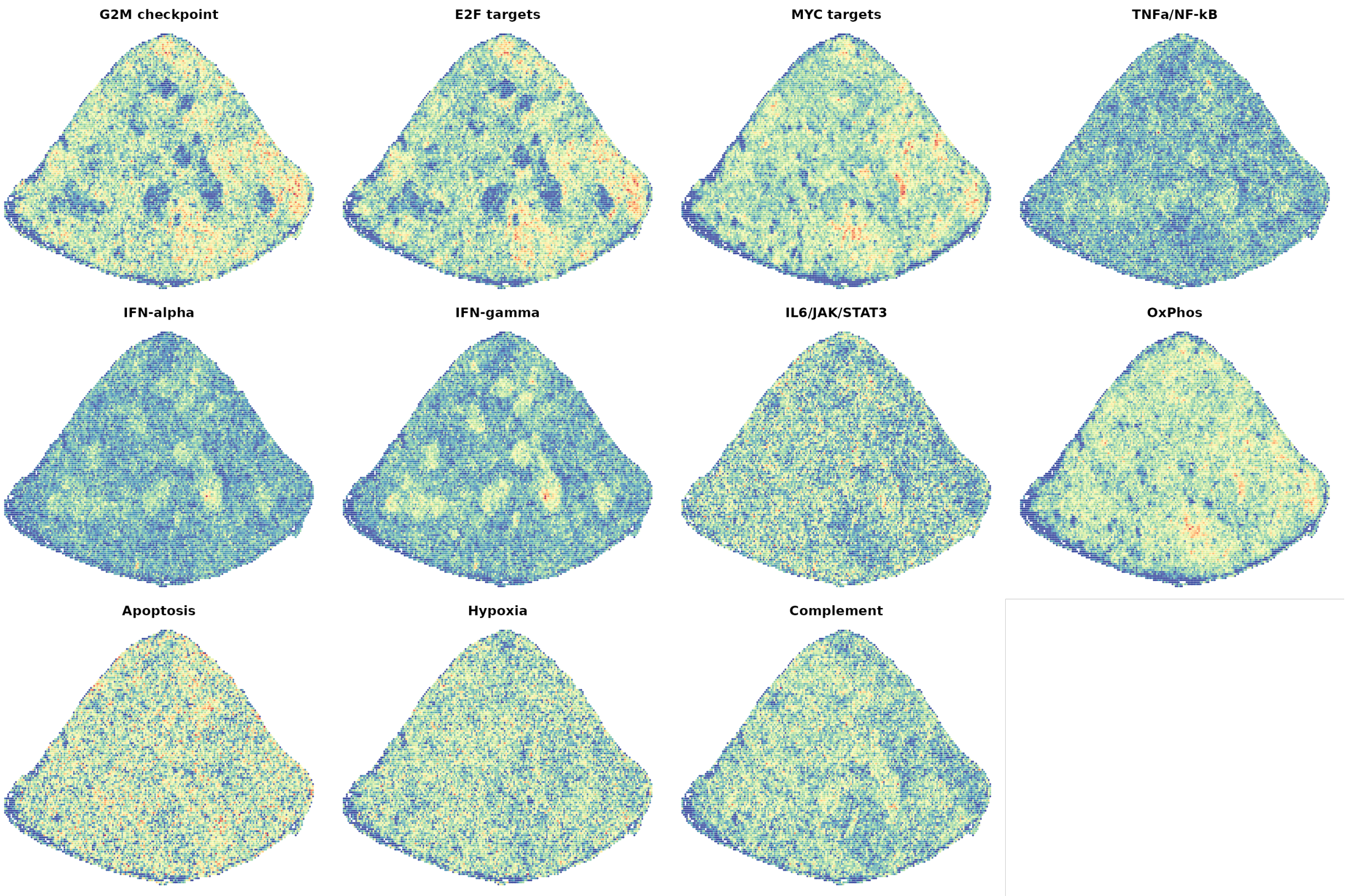

Fig S12: Spatial distribution of AML gene signatures, related to Figure 5. Spatial heatmaps of 11 Hallmark gene expression signatures scored using UCell.

Table S1: Niche-specific enriched cell types in all lung samples in CosMx case study, related to Figure 3.

	immune	lymphoid structure	macrophages	myeloid-enriched stroma	neutrophils	plasmablast-enriched stroma	stroma	tumor interior	tumor-stroma boundary
Lung5_Rep1	Alveolar Mph CCL3+	B cells	Non-classical monocytes	Alveolar Mph CCL3+	Alveolar Mph CCL3+	Plasma cells	Adventitial fibroblasts	AT1	AT1
	B cells	CD4 T cells	Alveolar Mph CCL3+	Non-classical monocytes	EC aerocyte capillary	Adventitial fibroblasts	Smooth muscle	Basal resting	Multiciliated (non-nasal)
	CD8 T cells	EC venous systemic	Monocyte-derived Mph	EC aerocyte capillary	Goblet (nasal)	Club (nasal)	Non-classical monocytes	Multiciliated (non-nasal)	Suprabasal
	EC aerocyte capillary	CD8 T cells	EC aerocyte capillary	NK cells	NK cells	Club (non-nasal)	DC2	Suprabasal	Basal resting
	CD4 T cells	Hillock-like	NK cells	EC general capillary	EC general capillary	Smooth muscle	Alveolar fibroblasts	Hillock-like	Smooth muscle
Lung5_Rep2	CD4 T cells	B cells		Alveolar Mph CCL3+	Alveolar Mph CCL3+	Plasma cells	Adventitial fibroblasts	AT1	AT1
	Suprabasal	CD4 T cells		Non-classical monocytes	EC aerocyte capillary	Club (nasal)	Smooth muscle	Basal resting	Multiciliated (non-nasal)
	NK cells	EC venous systemic		NK cells	NK cells	Adventitial fibroblasts	DC2	Multiciliated (non-nasal)	Basal resting
	Alveolar Mph CCL3+	CD8 T cells		EC aerocyte capillary	Goblet (nasal)	EC general capillary	Lymphatic EC mature	Suprabasal	Suprabasal
	AT1	EC venous pulmonary		EC general capillary	AT1	Alveolar fibroblasts	Alveolar macrophages	Hillock-like	Classical monocytes
Lung5_Rep3	CD4 T cells	B cells	Alveolar Mph CCL3+	Alveolar Mph CCL3+	Alveolar Mph CCL3+	Plasma cells	Adventitial fibroblasts	AT1	Smooth muscle
	Non-classical monocytes	CD4 T cells	EC aerocyte capillary	EC aerocyte capillary	EC aerocyte capillary	Club (nasal)	Smooth muscle	Basal resting	AT1
	Alveolar Mph CCL3+	EC venous systemic	NK cells	Non-classical monocytes	NK cells	AT2	DC2	Multiciliated (non-nasal)	Multiciliated (non-nasal)
	NK cells	pre-TB secretory	CD4 T cells	NK cells	Non-classical monocytes	Adventitial fibroblasts	Non-classical monocytes	Hillock-like	Basal resting
	EC aerocyte capillary	CD8 T cells	Goblet (nasal)	EC general capillary	EC general capillary	Plasmacytoid DCs	Alveolar fibroblasts	Suprabasal	Hillock-like
Lung6	Alveolar Mph CCL3+	Alveolar Mph CCL3+	Alveolar Mph CCL3+	Alveolar Mph CCL3+			Alveolar Mph CCL3+	Hillock-like	CD4 T cells
	Interstitial Mph perivascular	Plasma cells	Interstitial Mph perivascular	Interstitial Mph perivascular			Plasma cells	NK cells	Alveolar Mph CCL3+
	Plasma cells	Interstitial Mph perivascular	Alveolar macrophages	DC2			Interstitial Mph perivascular	Suprabasal	Classical monocytes
	DC2	CD4 T cells	DC2	AT2			CD4 T cells	Basal resting	Lymphatic EC mature
	CD4 T cells	B cells	Monocyte-derived Mph	Plasma cells			Lymphatic EC mature	Goblet (nasal)	Plasma cells
Lung9_Rep1	EC venous pulmonary	CD4 T cells	Monocyte-derived Mph	EC venous pulmonary	Monocyte-derived Mph	Plasma cells	EC venous pulmonary	pre-TB secretory	Adventitial fibroblasts
	Plasma cells	Plasma cells	Non-classical monocytes	EC arterial	EC venous pulmonary	B cells	EC aerocyte capillary	Neuroendocrine	Alveolar fibroblasts
	CD4 T cells	EC venous pulmonary	EC venous pulmonary	Non-classical monocytes	CD4 T cells	Smooth muscle	SMG duct	Deuterosomal	Smooth muscle
	EC aerocyte capillary	EC aerocyte capillary	EC arterial	SMG duct	Basal resting	EC venous pulmonary	EC general capillary	Tuft	Club (non-nasal)
	Alveolar fibroblasts	Lymphatic EC mature	SMG duct	EC aerocyte capillary	AT2	Multiciliated (non-nasal)	Alveolar Mph CCL3+	Hillock-like	EC general capillary
Lung9_Rep2	Alveolar fibroblasts	Alveolar fibroblasts		Alveolar fibroblasts	Non-classical monocytes	Plasma cells	Alveolar fibroblasts	pre-TB secretory	Alveolar fibroblasts
	Plasma cells	Plasma cells		DC2	DC2	Alveolar fibroblasts	Adventitial fibroblasts	Neuroendocrine	Plasma cells
	Non-classical monocytes	Non-classical monocytes		Non-classical monocytes	Alveolar fibroblasts	Non-classical monocytes	DC2	Hillock-like	Adventitial fibroblasts
	Adventitial fibroblasts	Adventitial fibroblasts		Adventitial fibroblasts	Alveolar Mph CCL3+	Adventitial fibroblasts	Smooth muscle	Deuterosomal	EC general capillary
	EC general capillary	DC2		Multiciliated (nasal)	Multiciliated (nasal)	Smooth muscle	Alveolar Mph CCL3+	Tuft	Alveolar macrophages
Lung12	Monocyte-derived Mph	Plasma cells		Plasma cells	CD4 T cells	Plasma cells	Adventitial fibroblasts	Goblet (nasal)	AT1
	Suprabasal	B cells		CD4 T cells	NK cells	B cells	Alveolar fibroblasts	AT1	Club (nasal)
	EC arterial	Monocyte-derived Mph		NK cells	CD8 T cells	EC venous pulmonary	EC general capillary	Classical monocytes	AT2
	NK cells	EC venous pulmonary		Non-classical monocytes	Non-classical monocytes	EC arterial	Alveolar macrophages	Monocyte-derived Mph	Classical monocytes
	CD8 T cells	EC arterial		EC venous systemic	DC2	Lymphatic EC mature	Smooth muscle	Basal resting	Smooth muscle
Lung13	Interstitial Mph perivascular		Monocyte-derived Mph	Monocyte-derived Mph	Club (nasal)		Adventitial fibroblasts	pre-TB secretory	AT2
	Plasma cells		Non-classical monocytes	pre-TB secretory	pre-TB secretory		Suprabasal	Club (nasal)	Goblet (nasal)
	DC2		Club (non-nasal)	Suprabasal	Multiciliated (non-nasal)		EC general capillary	Goblet (nasal)	AT1
	EC general capillary		Alveolar fibroblasts	CD8 T cells	CD4 T cells		Alveolar fibroblasts	EC arterial	Classical monocytes
	CD8 T cells		Pericytes	EC arterial	Suprabasal		Interstitial Mph perivascular	AT1	Club (nasal)

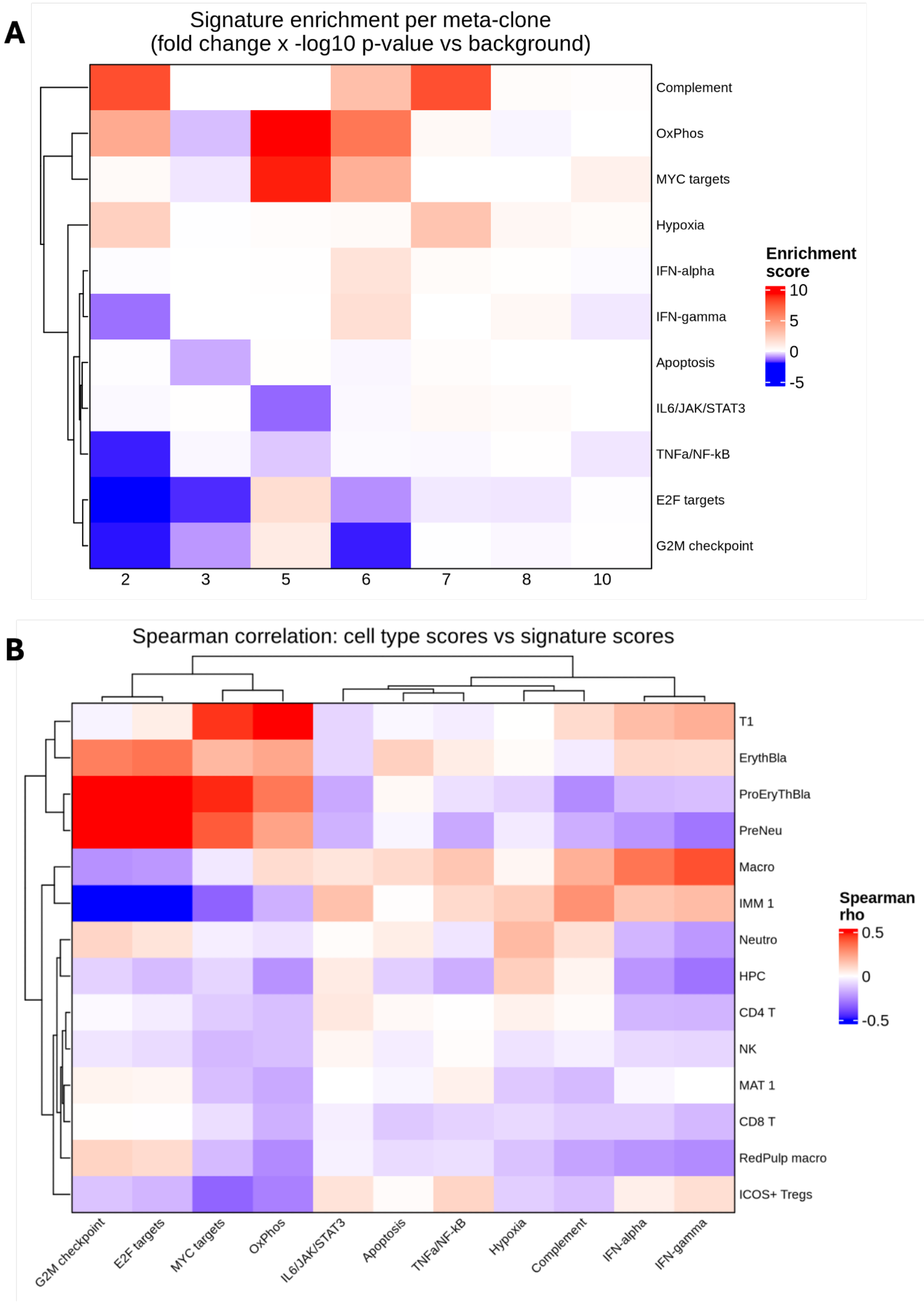

Fig S13: Enrichment of AML gene signatures in meta-clones, related to Figure 5. A: Signature enrichment per meta-clone: enrichment score = fold change $\times -\log_{10}$ p-value from Wilcoxon test vs background. **B:** Spearman correlations between selected cell type scores and signature scores, linking cell type identity to cellular state.

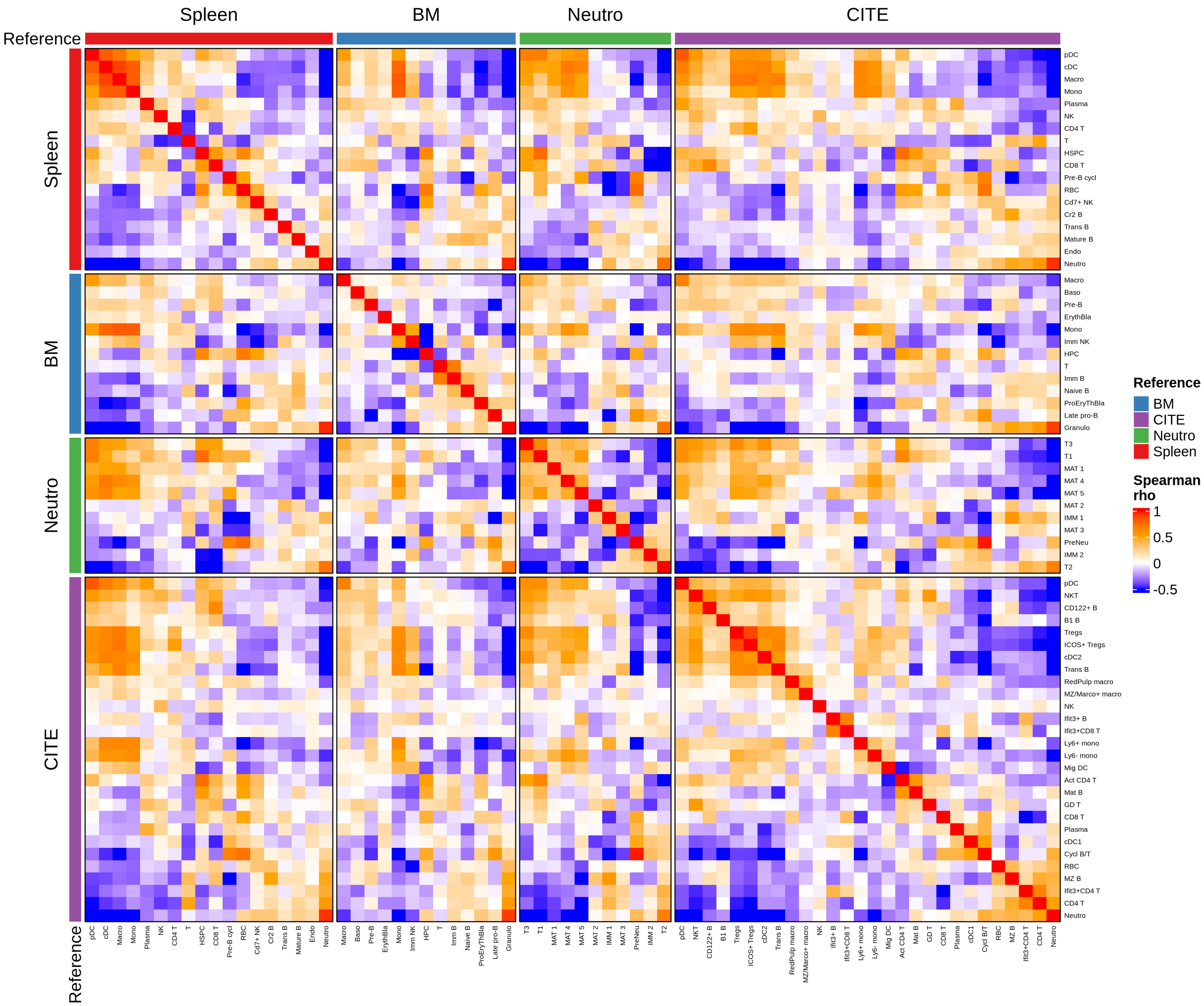

Fig S14: Correlation between cell type scores from multiple references, related to Figure 5. Spearman correlation heatmap of all 70 cell type scores across the 4 references (Spleen, BM, Neutro, CITE), computed on the intermediate scRNA-seq data (5,595 cells).

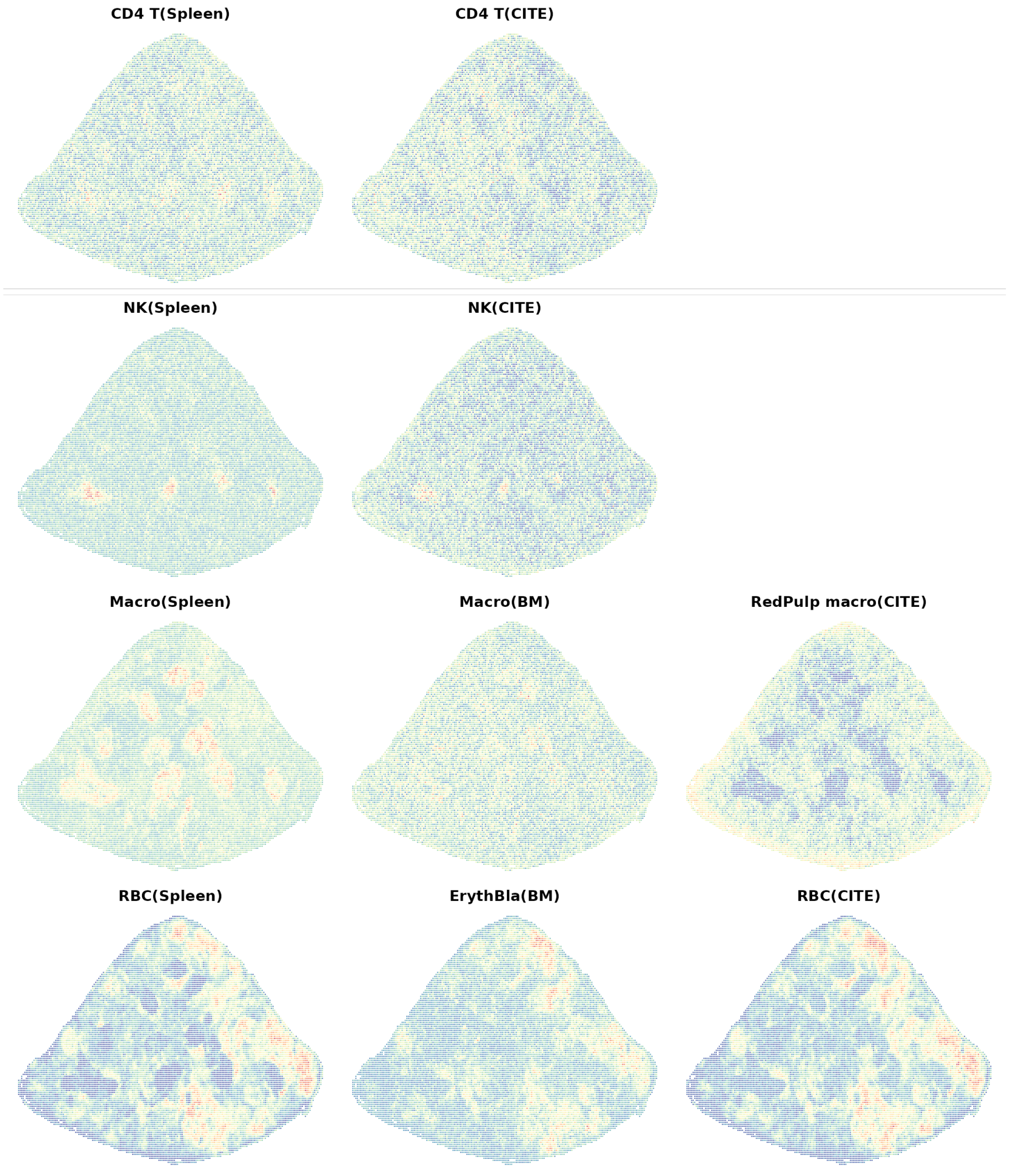

Fig S15: Spatial distribution of overlapping cell type groups, related to Figure 5. Side-by-side spatial heatmaps of Φ-Space scores for 4 representative overlapping cell type groups (CD4 T, NK, Macrophage, Erythrocyte) in the Stereo-seq data. Within each row, the same colour scale is used across references.

Table S2: Cross-reference Spearman correlations of Φ-Space scores, related to Figure 5. Spearman correlations between overlapping cell type scores across references, computed on the intermediate scRNA-seq data (5,595 cells). Selected pairs are shown; see Supplementary Fig S14 for all 29 pairs.

Cell type pair	References	Spearman ρ
Neutrophil	Spleen vs CITE	0.94
pDC	Spleen vs CITE	0.83
Monocyte	Spleen vs BM	0.81
cDC / cDC2	Spleen vs CITE	0.67
Monocyte / Ly6^{+} mono	Spleen vs CITE	0.63
Monocyte / Ly6^{+} mono	BM vs CITE	0.62
Macrophage	Spleen vs BM	0.38
NK	Spleen vs CITE	0.38
Mature B / Mat B	Spleen vs CITE	0.22
CD4 T	Spleen vs CITE	−0.38
cDC1 / cDC2 (control)	CITE vs CITE	−0.42
